# A cellular basis for the hourglass pattern in vertebrate embryogenesis

**DOI:** 10.1038/s41467-026-69828-9

**Published:** 2026-03-10

**Authors:** Amor Damatac, Kristian K. Ullrich, Alexander Klimovich, Markéta Kaucká

**Affiliations:** 1 Max Planck Institute for Evolutionary Biology, Ploen, Germany; 2 Zoological Institute, Christian Albrecht University of Kiel, Kiel, Germany

**Keywords:** Evolutionary developmental biology, Body patterning, Gastrulation

## Abstract

Vertebrate embryogenesis follows a conserved trajectory, exhibiting divergence in early and late stages and conservation during mid-embryogenesis across species. This pattern, known as the developmental hourglass, was first described at the morphological level and later supported by molecular studies, establishing it as a hallmark of vertebrate development. The “waist” of the hourglass, representing the period most resilient to evolutionary change, coincides with the emergence of the body plan, when embryos across species appear most alike. Yet development is not simply an organism-level process; it arises from the coordinated behaviors of individual cell lineages that collectively generate form and function. If the hourglass reflects a fundamental principle of vertebrate development, might it also be rooted in the dynamics of cells themselves? In this Perspective, we revisit the hourglass model through the lens of cellular lineages, asking whether the conservation of mid-embryogenesis is underpinned by universal constraints at the level of individual cells. Could the vertebrate developmental hourglass truly have a cellular basis?

## Abstract (web summary)

The developmental hourglass has long been seen at the level of embryos. By zooming in to single cells, this Perspective shows that mid-embryonic conservation arises from converging cell states constrained by ancient, pleiotropic gene programs, revealing the hourglass as a cellular phenomenon.

## Historical origins of the developmental patterns

The investigation of the developmental patterns can be dated back to the very beginnings of classical embryology. Aristotle, one of the first naturalists to systematically observe animal development, recognized that the form and complexity of an organism are not present at conception but emerge gradually throughout development^1^. He argued that an embryo is not a miniature version of the adult from the start, but acquires its structure, organs, and species-defining features as development proceeds.

In the 19th century, Karl Ernst von Baer conducted detailed comparative investigations of vertebrate embryos and noted that embryos of different species morphologically resemble one another at certain developmental stages^2^. He formulated Baer’s laws of embryology, which describe how embryos share general features early in development before diverging into more specialized forms, a pattern that inspired the *developmental funnel model* of embryogenesis (see Box 1). Later, Ernst Haeckel popularized this view through his well-known comparative embryo drawings and proposed the more radical notion that “ontogeny recapitulates phylogeny”^3^, a view now outdated but influential in shaping how embryologists thought about evolutionary conservation in development. These morphological observations laid the groundwork for the recognition of a conserved mid-embryonic stage, later characterized as the phylotypic period (see Box 2), representing a developmental window of highest morphological similarity among species within a phylum and reflecting a shared body plan^4–6^.

Box 1 Glossary **Asymmetric hourglass:** a variation of the developmental hourglass model in which divergence is not evenly distributed before and after the conserved mid-embryonic period. In this Perspective, the asymmetry pertains to late developmental stages, which exhibit greater divergence than early stages.

**Cell state:** a molecular profile of a cell type at a given developmental stage, representing a discrete point along a cellular trajectory. In the trajectory map, each cell state is represented as a node.

**Cellular trajectory:** a computationally reconstructed path that represents the progression of gene expression changes among cell states over developmental time. See refs. ^44,45^.

**Developmental funnel model:** a model of embryonic development proposing that early stages are the most conserved across species, with divergence increasing progressively over developmental time. In this view, embryos begin from a common, highly constrained starting point and gradually diversify into species-specific forms. See refs. ^2,68–70^.

**Developmental hourglass model:** a model of embryonic development in which embryos of related species diverge in form during early and late stages but converge to a highly similar morphology at a conserved mid-embryonic period (the phylotypic period). See refs. ^6,8,9,13^.

**Molecular hourglass:** a pattern of developmental conservation at the molecular level in which the mid-embryonic period exhibits maximal similarity across species. This mid-embryonic conservation manifests in two ways: (1) high conservation of expression among orthologous genes across species, and (2) predominant expression of evolutionarily ancient, conserved genes. See refs. ^10,11,12,27,36^.

**Neurula:** an early embryonic stage in vertebrate development that marks the beginning of the formation of key vertebrate structures. During this stage, the neural plate forms and folds into the neural tube—the precursor of the central nervous system—while other essential axial features begin to emerge, including the somites, notochord, and node. Neurula occurs around embryonic day 7.5 (E7.5) in mouse and approximately 10 hours post-fertilization (hpf) in zebrafish.

**Pharyngula:** a mid-embryonic stage in vertebrate development during which the foundational body plan is established. At this stage, the embryo exhibits key features of vertebrate morphology, including a tailbud, notochord, dorsal neural tube, segmented musculature, and pharyngeal arches. Major organ systems begin to form, and the general body framework is present, though still undergoing refinement. This stage corresponds to embryonic day 8.5 (E8.5) in mouse and 24 hours post-fertilization (hpf) in zebrafish.

**Pleiotropic genes:** genes that are broadly expressed across multiple cell states and developmental stages, contributing to diverse biological processes and phenotypes. See ref. ^19^.

**Transcriptome Age Index (TAI):** a metric used to quantify the evolutionary age of a transcriptome at a given developmental time or condition. It integrates gene expression data with the evolutionary ages of genes, typically assigned based on gene age inference methods such as genomic phylostratigraphy^71^, to calculate a weighted average “age” of the expressed gene sets. The stage with the lowest TAI is regarded as the phase when gene sets expressed is most evolutionarily oldest. See refs. ^11,72^.

**Transcriptome similarities:** a measure of conservation in gene expression that can be assessed in different contexts: (1) across species, by correlating the expression of orthologous genes across corresponding developmental stages; and (2) within a species, by correlating the transcriptomic profiles of cell states, either within the same developmental stage or across different stages. See refs. ^10,12^.

Box 2 The Phylotypic period The phylotypic period represents a span of embryogenesis during which members of a given phylum exhibit the highest degree of morphological similarity^8,9,60^. The concept was initially formulated as the *phylotypic stage* or *phylotype*, during which the blueprint of the body plan becomes recognizable^5,6,73^. Subsequent work revealed substantial heterochrony and variation in key structures across species^60^. As a result, the phylotypic stage is now understood as a broader developmental interval, the phylotypic period. Within the hourglass framework, this interval corresponds to mid-embryogenesis, situated between divergent early and late developmental stages.

There is a question whether the phylotypic period strictly corresponds to phylum-level boundaries or whether mid-embryonic conservation emerges within more narrowly defined clades^8,9,74^. Within Chordata, for example, comparative analyses suggest that mid-embryonic conservation, spanning from neurula to pharyngula (with the pharyngula traditionally recognized as the most conserved vertebrate stage^11,12,19^), is more consistent within subphylum Vertebrata^19^. This has prompted the view that vertebrates may warrant recognition as a distinct phylum^74^. Recent transcriptomic studies show that non-vertebrate chordates share aspects of mid-embryonic conservation with vertebrates^20,21^, suggesting that features of the vertebrate phylotypic pattern are conserved across the entire phylum.

A major limitation in resolving these debates is that most hourglass analyses rely on a narrow sampling of species^4,10,11,27,31,36^. As a result, the observed mid-embryonic conservation may reflect either a true phylum-level pattern or a clade-specific signal. There is evidence for persistent conservation within a phylum, with conserved developmental stages remaining even among distantly related species. Equivalent mid-embryonic stages can be recovered across diverse chordate/vertebrate groups, indicating the evolutionary conservation of these stages within the group^19^.

On the other hand, transcriptomic comparison across ten animal phyla revealed that mid-embryonic gene-expression profiles diverge across phyla, whereas early and late stages exhibit the opposite trend, resulting in an *inverse hourglass pattern*^4^. These findings suggest that each phylum may have its own characteristic phylotypic period, potentially aiding in the delimitation of phylum-level species groupings. This interpretation has been challenged on methodological grounds, specifically that one representative was sampled per phylum and that pairwise comparisons do not provide sufficient resolution to determine evolutionary relationships^75^.

Echinoderms display an hourglass-like pattern. However, their most conserved developmental phase occurs during gastrulation rather than during the formation of the pentaradial body plan^24^, indicating that developmental conservation and body plan establishment can be evolutionarily decoupled.

Together, these findings and arguments underscore longstanding debates over how higher-level taxa and body plans are defined, and how these definitions shape interpretations of the phylotypic period within the hourglass model.

## From funnel to hourglass

Early comparative embryological investigations largely overlooked the variation in the earliest phases of development, such as cleavage, blastula, and gastrula^7^. Careful reexamination of embryogenesis, including these early stages in the 20th century, revealed a distinct pattern, leading to the formulation of the *developmental hourglass model* (or “egg timer” model)^8,9^. This model describes embryonic development as divergent in the early and late stages, with a conserved “waist” in mid-embryogenesis. Duboule^8^ and Raff^9^ proposed influential explanations for the hourglass pattern. Duboule^8^ linked mid-embryonic conservation to *Hox* gene colinearity and body plan establishment, while Raff^9^ emphasized it as a developmental bottleneck, in which intricate signaling networks constrain evolutionary change.

**Fig. 1:**
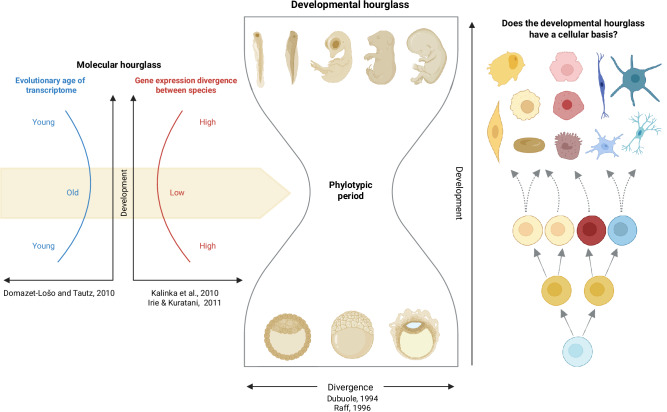
The developmental hourglass model. The concept is supported at the molecular level, where mid-embryonic conservation is characterized by high transcriptome similarity and the expression of evolutionarily older transcriptomes. The figure was created in BioRender. https://BioRender.com/3iztwfg.

A major shift in hourglass research occurred when two landmark transcriptome-level studies revealed key molecular signatures of the developmental hourglass (Fig. 1). Kalinka, et al.^10^ compared the temporal gene expression profiles of various *Drosophila* species across development and found that gene expression conservation is maximal during mid-embryogenesis, while Domazet-Lošo and Tautz^11^ developed a *transcriptome age index* (TAI) and revealed that sets of genes expressed at the mid-embryonic stage are evolutionarily older and more conserved than those expressed early or late.

Despite the distinct analytical approaches, both studies converged on a central finding that transcriptomic conservation and expression oldest genes occur during mid-embryogenesis. In vertebrates, Domazet-Lošo and Tautz^11^ and Irie and Kuratani^12^ provided molecular evidence for this conservation using such alternative approaches, which overlap with early comparative morphological work that proposed various candidate phylotypic stages within the mid-embryonic window^13–15^, although this has been challenged by later morphological comparisons that did not recover an hourglass conservation pattern^16,17^.

Nevertheless, over the past decade, subsequent transcriptomic studies have extended these observations across diverse taxa, including arthropods^10,11,18^, chordates^11,12,19–23^, echinoderms^24–26^, plants^27–30^, fungi^31^, molluscs^32^, nematodes^33–35^, and most recently brown algae^36^. Collectively, these studies establish the concept of the *molecular hourglass* and demonstrate its broad existence across multicellular organisms. The remarkable recurrence of this pattern across independently evolved multicellular lineages suggests that the hourglass reflects a fundamental organizational principle of complex multicellular development.

## From organismal to cellular level: a deeper view of embryogenesis

Transcriptomic studies of the hourglass pattern have primarily examined whole embryos, providing fundamental insights into developmental patterns and their molecular underpinnings, but only at the organismal level, averaging signals across all cells. Yet embryos are built from a single cell that undergoes successive divisions, with individual cells then progressively restricting their potential as they commit to certain specific cell lineages and specialized cell types. The behavior and fate decisions of these lineages in embryonic space and time drive the emergence of complex and conserved structures, while also generating species-specific morphologies. Viewing embryogenesis from the cellular perspective, therefore, offers a unique opportunity to understand how developmental patterns, such as the hourglass, emerge from the individual cells and their distinct gene expression programs (Fig. 1).

If the hourglass pattern represents a core principle of multicellular development, should it not also be evident at the level of individual cells, the fundamental building blocks from which complex organisms arise? The convergent evolution of both multicellularity and embryogenesis across animals, plants, and algae, all of which exhibit developmental hourglass patterns^37–39^, suggests that cellular programs underlying embryonic development may similarly reflect conserved organizational principles.

Can we now take advantage of a higher-resolution perspective and explore conservation and divergence patterns at the level of individual cells? Single-cell RNA sequencing (scRNA-seq) profiles gene expression in thousands of individual cells and, when applied across extended developmental timelines, allows reconstruction of *cellular trajectories* and lineage relationships, providing unprecedented resolution of cellular organization and dynamics along the developmental landscape^40–43^. With extensive temporal coverage datasets available, it has become possible to systematically infer cellular trajectories throughout embryogenesis^44,45^ and examine the molecular hourglass at single-cell resolution.

These temporally resolved datasets offer an opportunity to determine whether all cellular lineages follow a similar hourglass-like pattern with a conserved mid-embryonic phase, or instead display differences in timing and degree of conservation across lineages^33,46^. Ma and Zheng^33^ have provided the first empirical assessment of this question through a systematic analysis of *Caenorhabditis elegans* development using bulk and single-cell transcriptomic data. The authors found that, in addition to the whole-embryo hourglass, specific cellular lineages (e.g., hypodermal cells and neurons) also exhibit hourglass-like dynamics. These findings provide the first insight into the cellular basis of the organismal hourglass model, suggesting that the whole-organism pattern may emerge as the composite of cell-specific trajectories, or that certain lineages contribute more strongly to the hourglass pattern.

In this perspective, we leverage these methodological and conceptual advances and investigate the vertebrate hourglass model at the cellular level. By analysing gene expression dynamics along cellular developmental trajectories in mouse (*Mus musculus*) and zebrafish (*Danio rerio*), we seek to identify conserved and divergent molecular signatures across developmental *cell states* and explore the biological processes that may underlie the cellular-level hourglass pattern.

## Profiling cellular trajectories across vertebrate embryogenesis

**Fig. 2:**
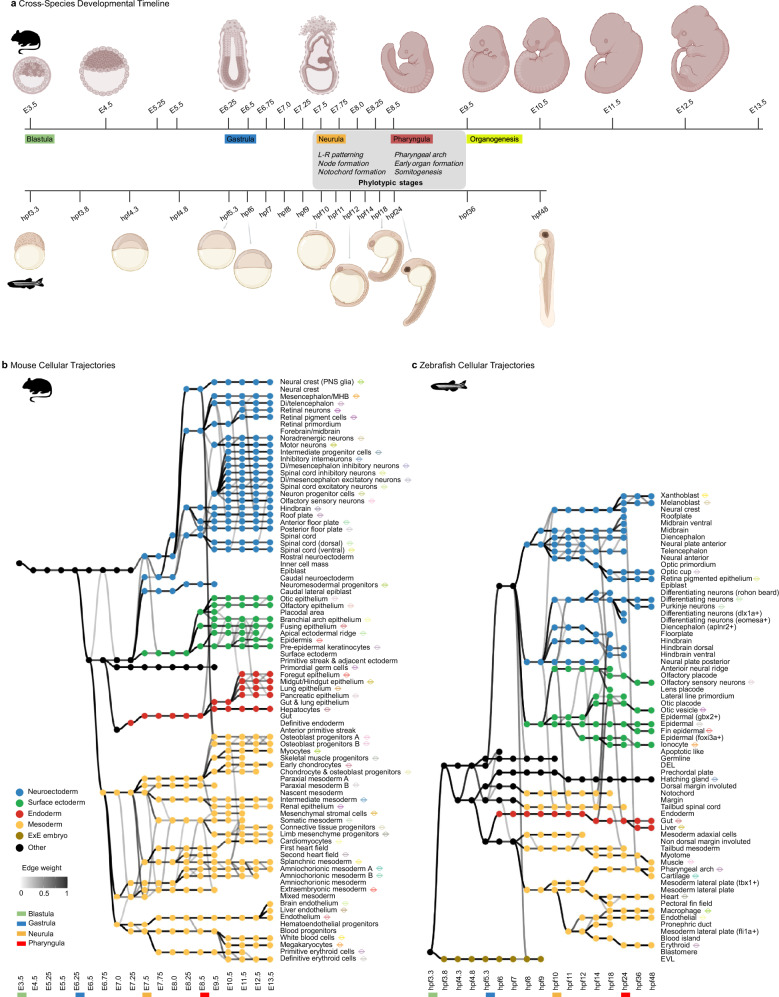
Single-cell transcriptome-based reconstruction of cellular trajectories in mouse and zebrafish embryogenesis. **a** The developmental stages analyzed in this study are aligned based on major developmental landmarks: blastula, gastrula, neurula, pharyngula, and organogenesis. **b**, **c** Cellular trajectory reconstructions of mouse (**a**) and zebrafish (**b**) embryogenesis. Nodes represent distinct cell states, colored by germ layer origin. The intensity of connecting edges reflects the strength of relationships between adjacent states, with only edges exceeding a weight threshold of 0.2 displayed. Markers next to cell type labels denote trajectories that were subjected to downstream analysis for the hourglass pattern. **a** was created in BioRender. https://BioRender.com/3iztwfg. Mouse and zebrafish silhouettes obtained from PhyloPic (https://www.phylopic.org); image credit Soledad Miranda-Rottmann and Ian Quigley, respectively (CC BY 3.0).

To investigate the cellular basis of this molecular hourglass in vertebrates, we utilized available single-cell transcriptome datasets spanning mouse and zebrafish development (Supplementary Note 1 and Supplementary Table 1). In the mouse, we included 19 developmental stages from embryonic day (E) 3.5 to 13.5, and in zebrafish, 17 stages from 3.3 to 48 h post-fertilization (hpf) (Fig. 2a). These datasets capture embryogenesis from the earliest through late developmental phases, including key transitions such as blastula, gastrula, neurula, pharyngula, and organogenesis. We reconstructed cellular trajectories using an adapted framework from Briggs et al.^45^ and Qiu et al.^44^ (Supplementary Note 2), generating 61 trajectories from 84 annotated cell types in mouse and 44 trajectories from 68 annotated cell types in zebrafish. This molecular roadmap captures the transitions of lineally related cell states over time, providing a temporally resolved view of cell state progression and underlying dynamic gene expression changes throughout vertebrate embryogenesis (Fig. 2b, c).

## A cellular basis of the molecular hourglass

### Cell states during neurula exhibit the highest transcriptome similarity

The central prediction of the molecular hourglass model is that gene expression programs should be most conserved during mid-embryonic stages, when the phylotypic period is being established^10^. We tested this prediction at cellular resolution by performing comparative single-cell transcriptomic analyses of mouse and zebrafish development. To initially assess overall *transcriptome similarities* between species and determine whether the molecular hourglass pattern observed in previous bulk, whole embryo transcriptomic studies is recapitulated, we initially pseudobulked the single-cell transcriptome data by stage and computed cross-species expression correlations (Supplementary Note 3).

**Fig. 3:**
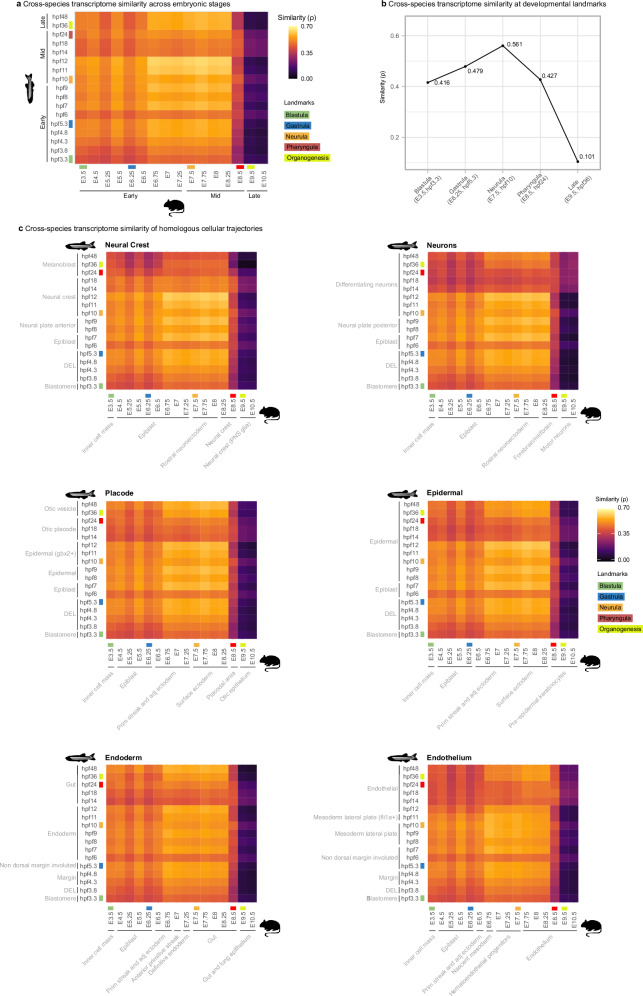
Transcriptome similarity between mouse and zebrafish embryos. **a** Heatmap of pairwise transcriptome similarity across stages of mouse and zebrafish, quantified using Spearman’s correlation coefficient (*ρ*), with higher values indicating greater similarity. **b** Comparative transcriptome similarity of major developmental landmarks between species. **c** Heatmaps of transcriptome similarity of homologous cellular trajectories between mouse and zebrafish. Note that some trajectories terminate in highly specialized cell types (e.g., glia in mouse and melanoblasts in zebrafish), which most likely contribute to the greater divergence observed in late-stage comparisons. Nevertheless, such divergence at terminal stages is biologically expected and reinforces, rather than undermines, the interpretation that similarity decreases later in development in part due to species-specific cellular diversification. Mouse and zebrafish silhouettes obtained from PhyloPic (https://www.phylopic.org); image credit Soledad Miranda-Rottmann and Ian Quigley, respectively (CC BY 3.0).

Because precisely aligning developmental stages between species is challenging^12^, we performed an all-to-all pairwise comparison. This analysis revealed that transcriptome conservation peaks during neurula, whereas early (blastula) and late (organogenesis) stages exhibit greater divergence (Fig. 3a and Supplementary Fig. 1). Notably, high transcriptome similarity is already detectable during gastrulation, a period not examined in earlier studies^12,19^, but in line with recent findings of transcriptional convergence among cell states leading into neurulation^23^. The onset of transcriptomic convergence during gastrulation precedes its peak observed at neurula. Refining this observation by selecting stages corresponding to major developmental events in each species confirmed that transcriptome similarity is highest at neurula (Fig. 3b). These findings support the classic developmental hourglass model and align with previous morphological studies identifying neurula as one of the most conserved stages in vertebrate development^14,15^, and consistent with previous comparative transcriptomic studies indicating that neurula is among the stages with the highest gene expression conservation^12,19–21^.

At the cellular level, we compared the transcriptomes of homologous cell type trajectories, including neural crest, neurons, placodes, epidermis, endoderm, and endothelium (Supplementary Fig. 2 and Supplementary Note 4). Across all homologous trajectories, mid-embryonic cell states, particularly during neurula, consistently exhibited maximal gene expression conservation across species (Fig. 3c). We observed that the overall pattern of transcriptome similarity across all lineages closely mirrors that detected at the organismal (pseudobulk) level, with all trajectories showing peak conservation during neurulation. This correspondence suggests that the whole-embryo hourglass pattern reflects a collective behavior of individual lineages, in which cell states converge toward similar transcriptional states during mid-embryogenesis.

The onset of neurulation marks the emergence of derivatives from all three germ layers, following their specification during gastrulation. At this point, the murine and zebrafish embryos with different early developmental strategies (i.e., holoblastic versus meroblastic cleavage, blastocyst formation versus blastoderm development, and primitive streak gastrulation versus epiboly^47,48^) converge at the transcriptional level. This convergence likely reflects the activation of deeply conserved orthologous gene regulatory networks required for coordinating germ layer derivatives and establishing the body plan. Thus, despite early morphological divergence and differences in cell type composition, vertebrate embryos align their cell state transcriptional programs as they approach neurulation, ensuring robust and conserved body plan formation at the onset of mid-embryogenesis.

**Fig. 4:**
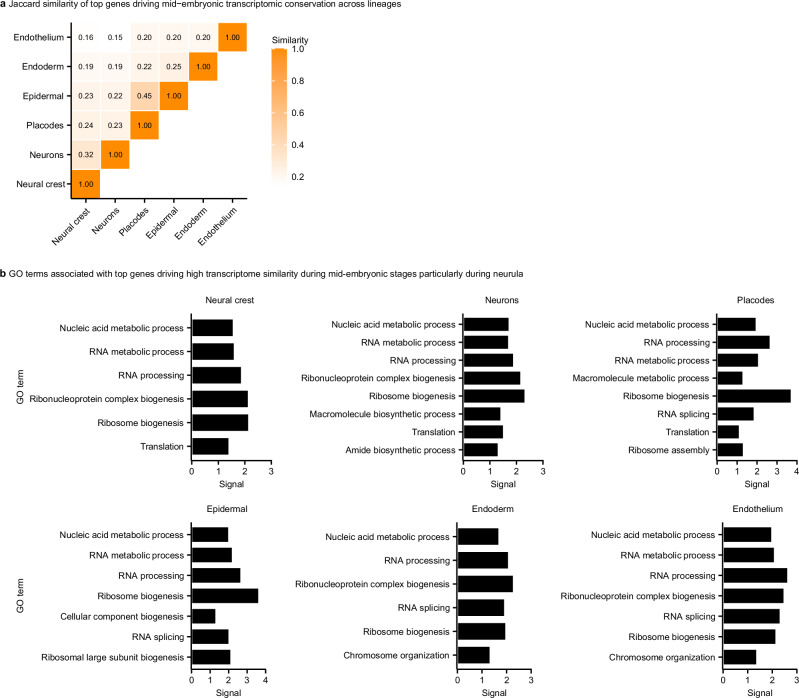
Comparing top genes driving high mid-embryonic transcriptome similarity across trajectories. **a** Jaccard index of gene set similarity among trajectories. A Jaccard index close to 1 indicates a high degree of overlap between the compared gene sets. The low Jaccard values here indicate minimal overlap in the specific genes contributing to high cross-species midembryonic similarity within each trajectory. **b** Enriched Gene Ontology (GO) terms for the top gene sets from each trajectory. Despite the low gene overlap, the gene sets are consistently associated with similar GO categories, highlighting convergence at the level of biological processes rather than individual genes. Importantly, randomly selected orthologous genes did not show the same GO enrichments, serving as a control (data not shown).

Given the striking similarity of the transcriptome similarity patterns across all analyzed trajectories, we asked whether the conserved transcriptome similarity observed during neurulation is jointly driven by a shared set of genes or arises independently within each individual trajectory. To examine this, we identified the top genes contributing to the highest cross-species correlations during mid-embryogenesis for all analyzed trajectories and compared the overlap among these gene sets. The overlap was minimal (Fig. 4a), indicating that although all trajectories display peak cross-species transcriptome similarity at this period, they do so using largely distinct sets of genes. Despite this limited gene-level overlap between non-homologous trajectories, GO enrichment analyses revealed that the trajectory-specific gene sets converge on similar biological functions, most prominently RNA metabolism, RNA processing, and translation (Fig. 4b). Thus, rather than relying on the same genes, different cellular trajectories appear to independently activate functionally conserved molecular processes, which in turn give rise to the similar patterns of cross-species transcriptomic similarities characteristic of mid-embryogenesis.

Moreover, later developmental stages, encompassing organogenesis, show greater divergence than early stages, suggesting an *asymmetric hourglass*. This pattern suggests that conservation begins to attenuate with the onset of organogenesis and an increase in cellular specification. It is also important to note that the cell-type composition of mouse and zebrafish becomes more diverse at these stages, which further contributes to the greater divergence observed in late development. Nevertheless, these observations confirm that the vertebrate developmental hourglass pattern has a cellular basis, with neurula emerging as a particularly critical stage in vertebrate development.

### Cell states during pharyngula express the most conserved genes

The molecular hourglass model encompasses not only patterns of expression conservation of orthologous genes but also the evolutionary age of genes expressed during development^11^. The TAI provides a framework to infer the evolutionary age of transcriptional programs across developmental time, allowing us to examine whether the distribution of gene ages across stages also follows an hourglass-like pattern.

**Fig. 5:**
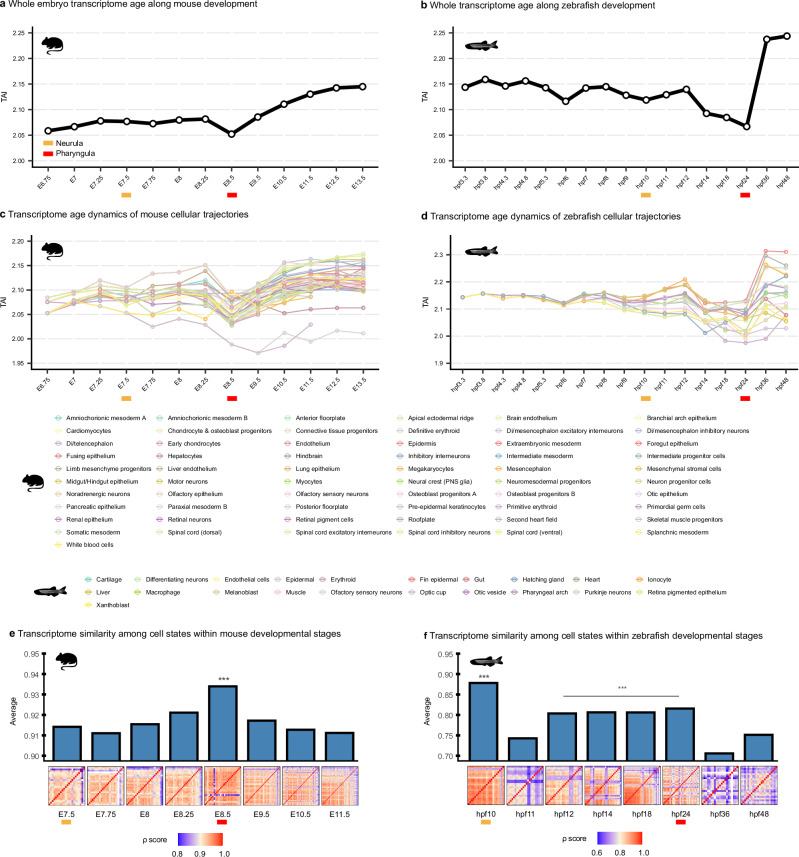
Phylotranscriptomic profiles of mouse and zebrafish embryogenesis. **a**, **b** Transcriptome Age Index (TAI) profiles across embryonic stages in mouse and zebrafish. **c**, **d** TAI profiles of cellular trajectories in mouse (**c**) and zebrafish (**d**). Each line represents the individual trajectories inferred in Fig. 2. **e**, **f** Transcriptome similarity of cell states within each developmental stage, quantified using Spearman’s correlation coefficient (*ρ*). Bars represent the average ρ value per stage, while the heatmap shows pairwise similarity among cell states within each stage, with red tiles indicating greater similarity. *** indicates stages with the highest ratios that are significantly different. Mouse and zebrafish silhouettes obtained from PhyloPic (https://www.phylopic.org); image credit Soledad Miranda-Rottmann and Ian Quigley, respectively (CC BY 3.0).

As previously described^11^, we applied TAI to examine how the evolutionary age of expressed genes varies along vertebrate embryogenesis using single-cell transcriptome data (Supplementary Notes 5, 6 and Supplementary Data 1). At the pseudobulk stage-level, both mouse and zebrafish embryogenesis recapitulate the whole-embryo hourglass signature^11^: a TAI profile in which mid-embryonic stages express the evolutionarily oldest gene sets, while earlier and later stages rely more on recently evolved genes (Fig. 5a, b). Notably, the evolutionarily oldest transcriptomes (lowest TAI) were observed during the pharyngula stages, consistent with their proposed role as a conserved period in vertebrate development^11–13^. In zebrafish, the lowest TAI extends from hpf14 to hpf24, indicating a slightly broader developmental period of expression of ancient conserved genes. An asymmetric hourglass pattern is also particularly evident: late developmental stages express significantly younger transcriptomes than early stages, reflecting the increasing contribution of recently evolved genes to cellular diversification during late development.

Taking advantage of cellular-level resolution data, we next extended the TAI analysis to individual cellular trajectories to determine whether the embryo-level hourglass pattern also holds at the level of cellular trajectories. Most trajectories recapitulate the whole embryo hourglass pattern, with pharyngula-stage cell states consistently expressing the evolutionarily oldest transcriptomes (Fig. 5c, d). The robustness of these patterns was confirmed using specific hourglass pattern tests, including flatline and reductive hourglass tests^29^ (Supplementary Note 7 and Supplementary Figs. 3−10).

While most trajectories followed this general pattern, a small number of trajectories displayed distinct deviations from the pharyngula-stage expression of the evolutionarily oldest gene sets (Supplementary Fig. 11). A consistent deviation from the hourglass profile was observed in blood and hepatic trajectories in both species, such as megakaryocytes, white blood cells, and definitive and primitive erythroid and hepatocytes in the mouse, and liver and erythroid cells in the zebrafish. The extraembryonic hatching gland in zebrafish also deviates from the pseudo-bulk hourglass profile. Notably, these trajectories did not show statistically supported hourglass patterns in the reductive hourglass test, indicating that their pharyngula-stage cell states do not exhibit the characteristic enrichment of evolutionarily oldest genes observed in most other trajectories.

A possible explanation for the blood and hepatic trajectories may lie in their shared association with hematopoiesis, a process that unfolds across distinct anatomical sites during embryogenesis. In vertebrates, hematopoiesis proceeds through primitive and definitive phases, with hematopoietic stem and progenitor cells sequentially emerging in extraembryonic (yolk sac) and intraembryonic niches (including fetal liver in mouse), each shaped by distinct molecular environments^49,50^. In both mouse and zebrafish, the transition to the definitive hematopoietic phase occurs around E10.5 and approximately hpf 26–30, respectively, which may underlie the onset of increasing TAI during these periods^49,51^. This dynamic development during hematopoiesis, involving relocation and associated transcriptional changes across developmental stages, likely results in a prolonged period of cycling through progenitor-like states compared to most other organs, in ways that cause these trajectories to diverge from the canonical hourglass pattern. In the mouse, the hematopoietic transition to the fetal liver, where definitive hematopoietic stem and progenitor cells (HSPCs) migrate, occurs around E11.5, which may explain the onset of increasing TAI at this stage. In zebrafish, although the liver is not a hematopoietic niche, hepatic differentiation occurs largely after hpf24, potentially sustaining older transcriptome activity beyond the pharyngula stage and leading to the deviation from the hourglass pattern^52^.

In summary, these results demonstrate that the molecular hourglass operates at the cellular level, with the whole embryo pattern emerging from the aggregate expression dynamics of multiple cellular trajectories. Most trajectories exhibit their highest expression of evolutionarily old genes during the pharyngula stage, collectively generating the organismal-level hourglass pattern, while a smaller subset contributes lineage-specific variation. These observations indicate that the association between the pharyngula stage and the enrichment of ancient transcriptomes, previously identified in comparative whole-embryo studies, is also reflected across the majority of individual cellular trajectories.

The single-cell TAI analysis also revealed heterogeneity in transcriptome age dynamics among trajectories across development. Beyond the lineages that deviate from the hourglass pattern, there is a general increase in the variability of TAI values among cell states as development progresses (Supplementary Fig. 12). This trend is consistent with expectations and a recent study^33^, as early embryonic cells originate from a shared pool of progenitors and therefore exhibit relatively uniform transcriptome ages.

In contrast, heterogeneity becomes more pronounced at later stages, particularly in zebrafish, where more differentiated cell states display a broader range of TAI values. In mouse, at E12.5- E13.5, the highest TAI values were observed in ectoderm-derived epithelial and neuronal, and endothelial cell types, whereas erythroid, myocyte, hepatocyte, and retinal cell types showed the lowest values (Supplementary Fig. 13). In zebrafish, at hpf36-hpf48, ectoderm-derived epithelial (epidermal) and neuronal, and endothelial cell types also exhibited the highest TAI values, while erythroid, optic cup, and liver cell types showed the lowest. Such variations reflect not only transcriptomic divergence associated with late-stage differentiation but also a conserved cross-species pattern in which neuronal, epithelial, and endothelial cell types express younger transcriptomes, while blood, muscle, and hepatic cell types express older ones.

These findings also suggest that germ-layer-specific transcriptomic signatures diminish as differentiation progresses, and that late-stage variation in TAI is primarily influenced by cell-type-specific processes rather than germ-layer origin, in agreement with recent observations in *C. elegans*^33^. The younger transcriptomes observed in ectoderm-derived neuronal lineages in both species may indicate the recruitment of more recently evolved genes associated with the expansion and functional diversification of the vertebrate neuronal architecture^53^. In contrast, mesoderm-derived muscle cell types may have retained core molecular and functional features from early animal ancestors, reflecting more ancient transcriptional programs^54,55^. A broader examination of transcriptome age differences among diverse late-stage cell types in the future, beyond the examples highlighted here, will be valuable, as such heterogeneity may capture the evolutionary histories and functional identities of distinct cell types, as demonstrated by recent single-cell phylotranscriptomic studies^33,56^.

A key question arises: why do pharyngula cell states simultaneously express the oldest gene sets? The answer becomes clear when examining transcriptome similarity among cell states within developmental stages (Supplementary Note 3). During the pharyngula stage, when transcriptome age becomes the oldest, cell states exhibit remarkably high transcriptome similarity despite representing a more advanced developmental stage with ongoing differentiation and the emergence of distinct cell types (Fig. 5e, f and Supplementary Figs. 14 and 15). This apparent paradox suggests a fundamental principle: the pharyngula represents a developmental phase where diverse cell states converge on shared transcriptional programs dominated by ancient, highly conserved genes. This convergence is not limited to pharyngula but extends to neurula stages, particularly in zebrafish, where the period of high transcriptome similarity among cell states spans the transition from neurulation through early pharyngula.

These findings indicate that during the onset of neurula and most prominently at pharyngula, cell states converge on a shared developmental program characterized by high transcriptome similarity and expression of ancient, evolutionarily conserved genes. Neurulation represents the onset of evolutionary conservation, whereas pharyngula reflects its culmination, aligning with the peak of morphological similarity across vertebrate embryos^13^. This convergence is particularly pronounced at pharyngula, immediately preceding organogenesis and marking the final phase of mid-embryogenesis.

The coincidence of transcriptional convergence among cell states within pharyngula and the expression of the oldest gene sets suggests that this stage represents a developmental bottleneck where cellular programs must align with evolutionarily ancient regulatory networks essential for body plan establishment. This pattern, now evident at the cellular level, explains why mid-embryonic stages previously identified as evolutionarily conserved in whole embryo studies are characterized by the enrichment of evolutionarily ancient genes. Paradoxically, while pharyngula exhibits the highest within-species transcriptome similarity among cell states and expresses the oldest genes, cross-species transcriptome similarity between mouse and zebrafish peaks earlier during neurula. At pharyngula, cell states begin to specify and specialize, likely contributing to the observed decrease in cross-species transcriptome similarity (Fig. 3a, b). Overall, both observations indicate that successful embryogenesis requires cell states to transiently coordinate their expression programs around a core set of evolutionarily conserved genes, ensuring robust body plan formation from neurula through pharyngula.

### Genes expressed during neurula and pharyngula are predominantly pleiotropic

**Fig. 6:**
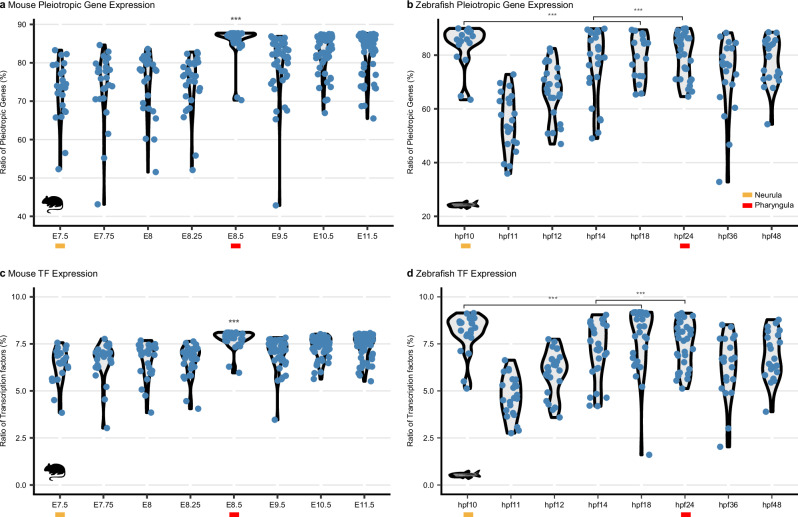
Pleiotropy and gene regulation activities during mid-embryogenesis. **a**, **b** Ratio of pleiotropic genes detected in mouse (**a**) and zebrafish (**b**) mid-embryonic stages. **c**, **d** Ratio of transcription factors (TFs) in mouse (**c**) and zebrafish (**d**) mid-embryonic stages. *** indicates stages with the highest ratios that are significantly different. Mouse and zebrafish silhouettes obtained from PhyloPic (https://www.phylopic.org); image credit Soledad Miranda-Rottmann and Ian Quigley, respectively (CC BY 3.0).

The conservation of mid-embryonic developmental programs has been linked to pleiotropic constraints^19^. We test this hypothesis by identifying the ratio of *pleiotropic genes* in mid-embryogenesis, defined as those broadly expressed across multiple cell states and lineages (Supplementary Note 8). Mid-embryonic cell states, particularly during pharyngula, express significantly higher proportions of pleiotropic genes (Fig. 6a, b), consistent with the previous bulk transcriptome study^19^. A similar trend is observed during neurula, notably in zebrafish, highlighting the broad deployment of pleiotropic genes during mid-embryogenesis.

Because pleiotropic gene expression likely requires tight regulatory control, we next examined the ratio of transcription factors (TFs) expressed across cell states (Supplementary Note 9). Indeed, the proportion of transcription factors expressed by cell states mirrors pleiotropic gene patterns, peaking during pharyngula and extending into neurula in zebrafish (Fig. 6c, d). This coordinated regulation suggests that transcription factors help orchestrate the broad expression of genes governing multiple biological processes.

Together, these observations indicate that the maintenance of conserved mid-embryonic cellular programs, when the bauplan is being established, is shaped by pleiotropic constraints and coordinated regulatory control across cell states. Although the exact mechanisms by which pleiotropic constraints are imposed and regulated remain unclear, the enrichment of pleiotropic genes and TFs supports their proposed role in constraining developmental variability^19,57,58^. Alterations in pleiotropic genes would have widespread effects across multiple cell states, thereby imposing constraints that limit developmental change and contribute to the evolutionary conservation of mid-embryonic programs^19^. In this view, neurula and pharyngula represent critical evolutionary bottlenecks where pleiotropy enforces robustness at the expense of flexibility, providing a mechanistic basis for the mid-embryonic conservation observed in the developmental hourglass.

### Functional enrichments of genes upregulated at the neurula and pharyngula

**Fig. 7:**
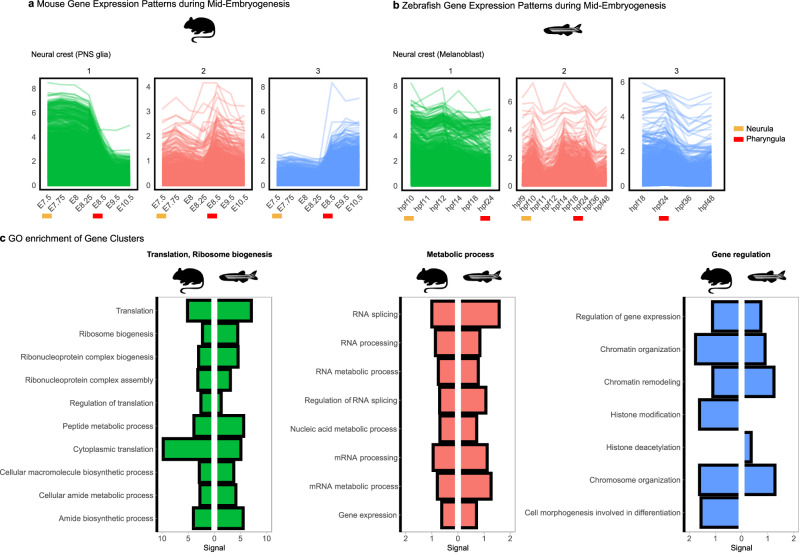
Gene expression dynamics and functional enrichments during mid-embryogenesis. **a**, **b** Distinct gene clusters consistently observed across cellular trajectories in mouse (**a**) and zebrafish (**b**), with representative examples from neural crest-derived PNS glia in mouse and melanoblasts in zebrafish. **c** Enriched Gene Ontology (GO) terms for each gene cluster, identified from genes expressed in at least 50% of analyzed trajectories per species, with a minimum expression value of ≥1. Colors correspond to the gene clusters shown in (**a**, **b**). Mouse and zebrafish silhouettes obtained from PhyloPic (https://www.phylopic.org); image credit Soledad Miranda-Rottmann and Ian Quigley, respectively (CC BY 3.0).

To understand the biological processes underpinning mid-embryonic conservation, we assessed the gene expression dynamics along reconstructed cellular trajectories and grouped the genes based on shared expression trends (Supplementary Note 10 and Supplementary Figs. 16 and 17). In both species, three distinct groups emerged, consistently upregulated during mid-embryogenesis: (1) genes upregulated during neurula (green), (2) genes upregulated in both neurula and pharyngula (red), and (3) genes highly expressed in pharyngula (blue) (Fig. 7a, b). Functional enrichment analysis revealed distinct roles for each group (Fig. 7c).

Neurula-upregulated genes were associated with translation and ribosome biogenesis, reflecting high biosynthetic demands in preparation for extensive cellular differentiation in the subsequent stages. Notably, these enrichments mirror the gene sets that contribute most strongly to cross-species transcriptome similarity at neurulation across trajectories, further suggesting that conserved mid-embryonic similarity at neurula arises from convergence on molecular programs. Genes expressed in both neurula and pharyngula were enriched for metabolic processes, particularly RNA-related functions, highlighting the energetic requirements of differentiation and tissue organization. This observation aligns with previous studies that have identified macromolecular metabolic processes as a key molecular pathway of the phylotypic period^10,59^. Pharyngula-specific genes were linked to transcriptional regulation, consistent with elevated transcription factor ratios at this stage, suggesting increased regulatory control of cell states to coordinate body plan establishment.

Notably, these functional enrichments were observed independently in both species, indicating deeply conserved molecular programs governing the mid-embryonic conservation. These results connect gene expression dynamics with the physiological and regulatory demands of mid-embryogenesis, providing a molecular explanation for the conservation observed at the neurula and pharyngula stages.

### Revisiting the developmental hourglass from a cellular perspective

**Fig. 8:**
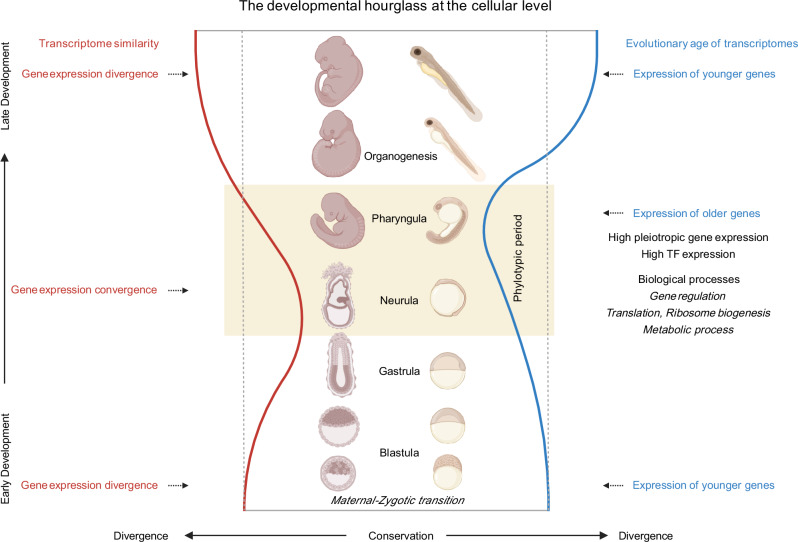
Cellular perspective of the hourglass model. This schematic highlights the hourglass pattern at the resolution of individual cellular trajectories, revealing two peaks of developmental conservation across vertebrate species: at the onset of neurulation (left) and at the onset of the pharyngula stage (right). The asymmetry of this model is also captured: later stages exhibit greater divergence than the early stages due to increased lineage-specific modifications. This asymmetry emphasizes how evolutionary constraints are strongest during mid-embryogenesis, while later stages provide greater developmental flexibility, facilitating species-specific adaptations. The figure was created in BioRender. https://BioRender.com/3iztwfg.

Over the past decade, considerable effort has focused on establishing the existence of the molecular hourglass across multicellular lineages. Here, we offer a new perspective by examining vertebrate development at single-cell resolution, shifting the focus from whole embryos to the dynamics of individual cellular trajectories. By analyzing mouse and zebrafish embryonic development, we show that individual cellular trajectories themselves exhibit a molecular hourglass pattern. These observations suggest that the hourglass reflects a fundamental principle of multicellular development at the cellular level, which may extend to other lineages where the hourglass pattern has been documented. The two distinct molecular signatures identified in this study refine our knowledge of the timing and nature of the evolutionarily and developmentally conserved phylotypic period in vertebrate embryogenesis (Fig. 8).

Our findings extend earlier work that rejects the notion of a single, well-defined phylotypic stage. The phylotypic period is now understood as a mid-embryonic interval during which conserved developmental features are progressively established^8,9,60^. By analysing gene expression at cellular resolution during vertebrate development, we show that both neurula and pharyngula stages contribute to this conserved developmental window. Neurulation marks the initial convergence of cell states and the employment of shared transcriptional programs, whereas the pharyngula stage reflects the final alignment of conserved developmental programs across cell states to establish the maximum similarity of the vertebrate body plan. These two stages represent distinct, unmatched molecular waist of the hourglass: cross-species transcriptome similarity peaks during neurula, while transcriptome age conservation and within-stage transcriptome convergence peak during pharyngula. Both stages, therefore, represent periods of heightened constraint, with each stage contributing to the overall conservation of the extended phylotypic period.

By providing evidence at the cellular level, this study demonstrates that the hourglass pattern exists at levels more granular than the whole embryo. This complements recent transcriptomic studies indicating that the hourglass model also extends to tissue and organ levels of developmental organization. For example, Cardoso-Moreira et al.^22^, though beginning their analyses at early organogenesis, compared gene expression across mammalian organs (cerebrum, cerebellum, heart, kidney, liver, ovary, and testis) across six mammals and found that molecular divergence increases as development progresses, with cross-species transcriptome correlations consistently declining over developmental time. Mukaigasa et al.^61^ showed that spinal cord development follows an hourglass pattern, with neuronal progenitors conserved across vertebrates but diverge as they mature, although their sampling in mouse (starting at E9.5) begins slightly after the vertebrate phylotypic window identified here. Similarly, Onimaru et al.^62^ demonstrated an hourglass-like conservation between limb and fin development, with mid-stage mouse limb buds (at E10.5) and shark fin buds exhibiting the highest transcriptomic similarity. While inclusion of earlier stages would clarify how these tissue-level patterns relate to the cellular-level signatures described here, the observed patterns may also reflect tissue-specific differences and heterochronic shifts in the timing of conservation, particularly in lineage-specific organs that may exhibit their own extended conserved phase in addition to the broader embryonic phylotypic period.

### The asymmetric hourglass: late development as the driver of morphological diversity

Notably, we also reveal a consistently asymmetric hourglass pattern, in which later developmental stages exhibit greater divergence than the early ones in both transcriptomic similarity and transcriptome age in both species (Fig. 8). While the hourglass model has been widely used to describe developmental conservation, its potential asymmetry has not been explicitly emphasized in previous studies. This asymmetric pattern likely results from increasing cell specialization and cell state composition at later stages, ultimately contributing to the phenotypic divergence of vertebrate embryos.

Our results suggest that while early development is relatively unconstrained, late development is shaped by even greater relaxation, facilitating the emergence of morphological diversity and lineage-specific features. This aligns with von Baer´s third law, highlighting that conservation is strongest when establishing foundational body plans, but as development progresses, species-specific modifications become more pronounced^16,63,64^. Although early developmental processes in mouse and zebrafish differ, recognizable developmental landmarks such as cleavage, blastula, and gastrula stages can be aligned across species.

In contrast, late-stage development diverges and is far more challenging to align reliably: after the pharyngula stage, zebrafish proceed through hatching, larval, and juvenile stages, whereas mouse embryos develop through distinct fetal stages in utero. The asymmetric hourglass pattern thus illustrates the dynamic interplay between conservation and divergence: early constraints set the vertebrate-specific developmental bauplan, while flexibility in later stages facilitates evolutionary innovation and morphological diversity.

However, it is important to note that previous whole-embryo transcriptome studies have reported that early divergence is strongest during the maternally dominated zygote and cleavage stages^12,65,66^, which could shift the degree of asymmetry observed here. The single-cell datasets analyzed in this work begin at the blastula stage, after most maternal transcripts have been degraded and zygotic transcription is underway^67^. As a result, the asymmetric hourglass described here reflects post-zygotic developmental dynamics. Incorporating single-cell datasets from earlier developmental stages in future work will be important for determining how maternal contributions influence the cellular hourglass pattern.

### Outlook

Together, we confirm that the developmental hourglass of vertebrate embryogenesis has a cellular basis and uncover its underlying molecular underpinnings. By demonstrating that the hourglass pattern is evident not only in whole-embryo but also within individual cell lineages, we move from a whole-organism view to a reductionist perspective, where the conserved dynamics of development can be traced to the level of specific lineages and cell states. Importantly, the organismal hourglass profile appears to emerge as the sum of its parts, with the majority of cellular lineages following the pattern rather than it being solely a feature of the whole embryo. At the same time, resolving developmental trajectories at cellular resolution reveals cell-level bottlenecks in vertebrate embryogenesis and points to the existence of “phylotypic cell states”.

This perspective raises important conceptual questions. How does resolving the hourglass at cellular resolution transform our view of development? What constraints restrict the evolvability of mid-embryogenesis, and why do cell states converge on conserved transcriptional programs at this stage? Addressing these questions will require connecting the fine-grained information from individual cells and trajectories back into an integrated view of the developing embryo. Doing so will allow us to understand not only how conserved programs are executed at the cellular scale but also how their coordination across tissues ensures robust body plan formation.

Although our perspective has centered on vertebrates, the next task is to test whether similar cellular underpinnings shape the hourglass pattern in other multicellular lineages. With the rapidly increasing availability of single-cell transcriptomic data, research on developmental patterns stands at the threshold of a new era, where conserved programs can be explored across cell states, tissues, and species, offering new insights into how developmental conservation shapes both the unity and diversity of life.

### Reporting summary

Further information on research design is available in the Nature Portfolio Reporting Summary linked to this article.

## Supplementary information

Supplementary Information

Description of Additional Supplementary Files

Supplementary Data 1

Reporting Summary

## Data Availability

The study utilizes publicly available software.
