# Supplementary material for: A cellular basis for the hourglass pattern in vertebrate embryogenesis
Source: Nat Commun. 2026 Mar 10;17:2404. doi: 10.1038/s41467-026-69828-9 (PMC12982620; doi:10.1038/s41467-026-69828-9)
Supplement: Supplementary file 1 — Supplementary Information [file 41467_2026_69828_MOESM1_ESM.pdf]

Supplementary Information
A cellular basis for the hourglass pattern in vertebrate embryogenesis

This PDF file contains Supplementary Notes, Supplementary Table, and Supplementary Figures.

Table of Contents

Supplementary Notes 2
Supplementary Note 1: Single-cell transcriptome datasets 2
Supplementary Note 2: Reconstruction of cellular trajectories 3
Supplementary Note 3: Transcriptome similarity analysis 4
Supplementary Note 4: Identification of homologous cell-type trajectories between mouse and zebrafish 5
Supplementary Note 5: Transcriptome Age Index 6
Supplementary Note 6: Gene age inference 7
Supplementary Note 7: Statistical assessment of the Transcriptome Age Index (TAI) 7
Supplementary Note 8: Detection of temporally pleiotropic genes 8
Supplementary Note 9: Detection of transcription factors 8
Supplementary Note 10: Gene Expression Dynamics and Functional Enrichment Analyses 8
Supplementary References 10
Supplementary Table 1 12
Supplementary Figures 13

Supplementary Notes

Supplementary Note 1: Single-cell transcriptome datasets

In this study, we used the processed datasets from Qiu, et al.[1] with modifications. Single-cell RNA sequencing datasets from *Mus musculus* (mouse) and *Danio rerio* (zebrafish) were acquired from previously published datasets (Supp. Table 1). The mouse datasets covered embryogenesis from 3.5 to 13.5 days post-fertilization (E3.5-E13.5). The zebrafish datasets spanned development from 3.3 to 48 hours post-fertilization (hpf3.3-hpf36). The modifications include the following:

Mouse

In the original study, the authors performed cellular trajectory analysis using two E8.5 (E8.5a and E8.5b) substages, with E8.5b introduced to allow a successful connection between E8.5a and the subsequent E9.5. Upon inspecting the dataset, we observed a one-to-one correspondence in cell state annotations between these two substages. To streamline downstream analyses and ensure a single representative stage, we integrated E8.5a and E8.5b into a unified E8.5 stage while preserving original cell state annotations. To note, the E8.5b dataset in this analysis included cells from sampled embryos at somite stages 7–12, spanning developmental timepoints between E8.5 and E8.75. This period aligns with the pharyngula stage in mouse development. Such integration was successful, as the 30 identical cell states from the two substages integrated into single clusters, preserving the original annotations necessary for the lineage trajectories.

Zebrafish

The original analysis analyzed datasets from stages hp3.3-hpf24. Because this window ends before pharyngula, it is insufficient for evaluating whether individual trajectories exhibit an

hourglass pattern, requiring to include additional stages beyond mid-embryogenesis. To extend the developmental timeline and capture later cell state transitions, we incorporated hpf36 and hpf48 datasets from Sur et al.[2]. These additional stages were integrated into the trajectory analysis following hpf24, allowing for an extended view of developmental progression. Although the addition of hpf36 and hpf48 expanded the developmental coverage, only a subset of trajectories could be confidently extended into the later stages, with most zebrafish trajectories ending at hpf24. Nevertheless, the extended subset provides sufficient coverage to evaluate the cellular hourglass pattern, and the trajectories that terminate earlier were still included in downstream analyses.

Finally, based on prior knowledge from the original study[1], we excluded lineages that did not progress until mouse E8.5 and zebrafish hpf24, as they lacked sufficient temporal resolution for downstream analyses.

Supplementary Note 2: Reconstruction of cellular trajectories

We reconstructed cellular trajectories across mouse and zebrafish embryogenesis using the strategy originally implemented by Briggs, et al.[3] and Qiu, et al.[1]. To capture transcriptional state transitions over development, a k-nearest neighbor (k-NN) heuristic approach was employed to connect cell states between adjacent developmental stages. This method was applied iteratively across all pairs of adjacent stages, producing a continuous molecular roadmap of individual cellular trajectories for each species. Developmental transitions between cell states were quantified using edge scores, which were then used to construct a cell state trajectory tree for each species, illustrating the progression of transcriptional states and lineage transitions throughout embryogenesis.

This trajectory reconstruction approach follows the publicly available scripts of Qiu, et al.[1], which include explicit evaluations of the robustness of edge weights:

(i) edge weights were recomputed across different co-embedding spaces (UMAP vs. PCA), yielding highly correlated results (Pearson r = 0.993);

(ii) the k-nearest neighbor step was repeated using multiple k values (k = 5, 8, 10, 15, 20), with resulting edge weights remaining highly correlated (r = 0.9994–0.9999); and

(iii) 500 rounds of bootstrapping with 80% subsampling were performed, and median proportions were used to obtain stable edge-weight estimates.

We adopted the same workflow in this study and, despite minor modifications (such as the removal of certain cell types and the addition of developmental stages), we recapitulated the same overall trajectory structure. These evaluations demonstrate that the inferred connections are sufficiently robust to represent stable pseudo-lineages across parameter choices. However, we note that trajectory inference remains a graph-based approximation of developmental relationships rather than a reconstruction of actual cell lineages, and therefore carries methodological limitations inherent to such approaches[1].

Supplementary Note 3: Transcriptome similarity analysis

To perform cross-species comparisons, we followed the pipeline established by Irie and Kuratani[4], based on identifying 1:1 orthologs between mouse and zebrafish using a reciprocal best BLAST hit (RBBH) search with CRBHits package[5]. The longest peptide isoform for each gene was used for ortholog detection, and mmseqs2[6] was selected as the sequence search tool.

Transcriptome similarity was quantified using the nonlinear Spearman correlation coefficient (ρ), with higher ρ values indicating greater similarity. For cross-species comparisons, we conducted two analyses: (1) comparing transcriptome similarity between whole embryonic stages in mouse and zebrafish, and (2) comparing transcriptome similarity along the cellular

trajectories of homologous cell types between species. To calculate ρ scores, single-cell transcriptome data were pseudobulked by aggregating counts either across each embryonic stage (analysis 1) or across each cell state (analysis 2), followed by TMM-based log normalization [$\log_2(x + 1)$][7].

Separately for each species, we also assessed transcriptome similarity among cell states within each developmental stage (for example, comparing transcriptome similarity of cell states within mouse E8.5), providing a view of within-stage transcriptome conservation.

Supplementary Note 4: Identification of homologous cell-type trajectories between mouse and zebrafish

To identify homologous cell-type trajectories between mouse and zebrafish, we first referred to the homologous cell type annotations previously established by Qiu, et al.[1]. To further validate these assignments, we applied SAMap (Self-Assembling Manifold Mapping), an algorithm specifically designed for cross-species comparison of single-cell transcriptomic data (Supp. Fig. 2)[8]. SAMap analysis was performed using the publicly available implementation and pipeline provided in the GitHub repository (https://github.com/atarashansky/SAMap/).

Following cross-species mapping, we extracted trajectories corresponding to the identified homologous cell types. Representative trajectories selected for downstream analysis included neural crest, neurons, placodes, epidermis, endoderm, and endothelium. These homologous trajectories enabled direct comparison of transcriptome similarity across cell states.

Supplementary Note 5: Transcriptome Age Index

The Transcriptome Age Index (TAI) was calculated as the weighted average evolutionary age of the transcriptome, where each gene's age was weighted by its expression level[9]. The relative evolutionary age of each gene in mouse and zebrafish was assigned based on an orthogroup-based gene age mapping approach (Supp. Note 6).

Using *myTAI*[10], TAI was calculated based on the original formula[9] as follows:

$$TAI = \frac{\sum_{i=1}^{n} ps_i e_{ic}}{\sum_{i=1}^{n} e_{ic}}$$

where the ps_i represents the phylostratum assignment of the gene i and e_{ic} denotes the expression level of a gene i in a cell (c), and n is the total number of expressed genes in a cell/stage (c).

Expression levels were obtained using log-normalized values. To assess TAI patterns throughout embryogenesis, we applied several single-cell transcriptome transformations. First, we performed a pseudobulk transformation by embryonic stage, where expression matrices were aggregated by stage and log-normalized ($\log_2(x + 1)$) following Trimmed Mean of M-values (TMM) scaling[7]. Next, we applied a pseudobulk transformation by cell state, aggregating expression by cell state and log-normalizing TMM-scaled counts. In mouse, stages E3.5 to E6.5 were excluded from the TAI analysis because these samples were generated using non-UMI sequencing (G&T-seq), making their count values difficult to compare with those from other stages.

Supplementary Note 6: Gene age inference

We inferred the relative evolutionary age of genes in mouse and zebrafish using an orthogroup-based mapping approach implemented with *oggmap*[11] (Supp. Data 1). Genes from both species were assigned to clusters of orthologous groups (COGs) based on eggNOG database[12], which organizes genes into orthogroups based on sequence homology and evolutionary relationships. The evolutionary age of each orthogroup was then determined from the position of its last common ancestor (LCA) in the consensus phylogeny, and this age was assigned to all member genes. This orthogroup-based classification ensures that homologous genes across species share the same evolutionary age, providing a robust framework for cross-species comparisons of developmental gene expression. We used these evolutionary age annotations with single-cell transcriptomic data to calculate the Transcriptome Age Index (TAI), enabling quantitative assessment of molecular conservation and divergence during embryogenesis.

Supplementary Note 7: Statistical assessment of the Transcriptome Age Index (TAI)

We assessed the statistical significance of TAI profiles using the flatline test and the reductive hourglass test as implemented in myTAI[10]. All tests were performed using 10,000 permutations (Supp. Fig. 3-10).

Initially, the flatline test for TAI in mouse cellular trajectories did not show significant support for an hourglass pattern, although the reductive hourglass test did. Suspecting that the discrepancy may be caused by technical noise from the sequencing data, we applied noisyR[13] to filter out genes with noisy expression. After filtering, both tests supported the hourglass pattern in mouse and zebrafish (Supp. Fig. 7-10). For zebrafish trajectories that terminate at 24 hpf, statistical testing could not be performed because the reductive hourglass test requires later developmental stages to assess whether an hourglass pattern is present. Nevertheless, most

trajectories for which testing was possible demonstrate the statistical significance of the hourglass pattern along cellular trajectories, with the pharyngula stage representing the "waist" at which cell states express the most evolutionarily ancient gene sets.

Supplementary Note 8: Detection of temporally pleiotropic genes

To identify temporally pleiotropic genes along mid-embryogenesis, we analyzed the developmental window spanning neurulation to early organogenesis (E7.5–E11.5 in mouse; 10–48 hpf in zebrafish). A gene was classified as pleiotropic if it was expressed in more than 50% of the cell states within this interval. For each developmental stage, we calculated the pleiotropic ratio by dividing the number of pleiotropic genes detected in each cell state by the total number of genes detected at that stage.

Supplementary Note 9: Detection of transcription factors

To quantify transcription factor (TF) expression during mid-embryogenesis, we used the TF annotations for mouse and zebrafish from Qiu, et al.[1] and assessed their expression across cell states from neurulation to early organogenesis (E7.5–E11.5 in mouse; 10–48 hpf in zebrafish). For each developmental stage, the TF ratio was calculated by dividing the number of expressed TFs in each cell state by the total number of genes detected at that stage.

Supplementary Note 10: Gene Expression Dynamics and Functional Enrichment Analyses

To investigate the biological processes associated with the upregulated genes during mid-embryogenesis, we first grouped genes with similar expression patterns using k-means

clustering[14]. Clustering was performed on selected cellular trajectories that were identified based on homologous cell states between mouse and zebrafish, determined through SAMap[8] (Supp. Fig. 16-17) and prior annotations from Qiu et al.[1]. We identified gene clusters that were consistently present across trajectories and extracted genes that showed persistent upregulation. Only genes that were upregulated in at least 50% of the analyzed trajectories for each species were included, and we applied a minimum expression threshold of ≥1. The resulting gene sets for mouse and zebrafish were independently subjected to Gene Ontology (GO) enrichment analysis using STRING-db (https://string-db.org)[15].

Supplementary References

1 Qiu, C. X. *et al.* Systematic reconstruction of cellular trajectories across mouse embryogenesis. *Nat Genet* **54**, 328-+, doi:10.1038/s41588-022-01018-x (2022).

2 Sur, A. *et al.* Single-cell analysis of shared signatures and transcriptional diversity during zebrafish development. *Dev Cell* **58**, doi:10.1016/j.devcel.2023.11.001 (2023).

3 Briggs, J. A. *et al.* The dynamics of gene expression in vertebrate embryogenesis at single-cell resolution. *Science* **360**, 980-+, doi:ARTN eaar5780
10.1126/science.aar5780 (2018).

4 Irie, N. & Kuratani, S. Comparative transcriptome analysis reveals vertebrate phylotypic period during organogenesis. *Nat Commun* **2**, doi:ARTN 248
10.1038/ncomms1248 (2011).

5 Ullrich, K. K. CRBHits: from conditional reciprocal best hits to codon alignments and Ka/Ks in R. *The Journal of Open Source Software* **5** (2020).

6 Steinegger, M. & Söding, J. MMseqs2 enables sensitive protein sequence searching for the analysis of massive data sets. *Nat Biotechnol* **35**, 1026-1028, doi:10.1038/nbt.3988 (2017).

7 Chen, Y. S., Chen, L. Z., Lun, A. T. L., Baldoni, P. L. & Smyth, G. K. edgeR v4: powerful differential analysis of sequencing data with expanded functionality and improved support for small counts and larger datasets. *Nucleic Acids Res* **53**, doi:ARTN gkaf018
10.1093/nar/gkaf018 (2025).

8 Tarashansky, A. J. *et al.* Mapping single-cell atlases throughout Metazoa unravels cell type evolution. *Elife* **10**, doi:ARTN e66747
10.7554/eLife.66747 (2021).

9 Domazet-Loso, T. & Tautz, D. A phylogenetically based transcriptome age index mirrors ontogenetic divergence patterns. *Nature* **468**, 815-U107, doi:10.1038/nature09632 (2010).

10 Drost, H. G., Gabel, A., Liu, J. L., Quint, M. & Grosse, I. myTAI: evolutionary transcriptomics with R. *Bioinformatics* **34**, 1589-1590, doi:10.1093/bioinformatics/btx835 (2018).

11 Ullrich, K. K. & Glytnasi, N. E. oggmap: a Python package to extract gene ages per orthogroup and link them with single-cell RNA data. *Bioinformatics* **39**, doi:ARTN btad657
10.1093/bioinformatics/btad657 (2023).

12 Hernández-Plaza, A. *et al.* eggNOG 6.0: enabling comparative genomics across 12 535 organisms. *Nucleic Acids Res* **51**, D389-D394, doi:10.1093/nar/gkac1022 (2023).

13 Moutsopoulos, I. *et al.*: enhancing biological signal in sequencing datasets by characterizing random technical noise. *Nucleic Acids Res* **49**, doi:ARTN e83
10.1093/nar/gkab433 (2021).

14 Hartigan, J. A. & Wong, M. A. A K-Means Clustering Algorithm. *Journal of the Royal Statistical Society Series C: Applied Statistics* **28**, 100-108, doi:10.2307/2346830 (2018).

15 von Mering, C. *et al.* STRING: a database of predicted functional associations between proteins. *Nucleic Acids Res* **31**, 258-261, doi:10.1093/nar/gkg034 (2003).

16 Mohammed, H. *et al.* Single-Cell Landscape of Transcriptional Heterogeneity and Cell Fate Decisions during Mouse Early Gastrulation. *Cell Rep* **20**, 1215-1228, doi:10.1016/j.celrep.2017.07.009 (2017).

17 Cheng, S. L. *et al.* Single-Cell RNA-Seq Reveals Cellular Heterogeneity of Pluripotency Transition and X Chromosome Dynamics during Early Mouse Development. *Cell Rep* **26**, 2593-+, doi:10.1016/j.celrep.2019.02.031 (2019).

18 Pijuan-Sala, B. *et al.* A single-cell molecular map of mouse gastrulation and early organogenesis. *Nature* **566**, 490-+, doi:10.1038/s41586-019-0933-9 (2019).

19 Cao, J. Y. *et al.* The single-cell transcriptional landscape of mammalian organogenesis. *Nature* **566**, 496-+, doi:10.1038/s41586-019-0969-x (2019).

20 Farrell, J. A. *et al.* Single-cell reconstruction of developmental trajectories during zebrafish embryogenesis. *Science* **360**, 979-+, doi:ARTN eaar3131 10.1126/science.aar3131 (2018).

21 Wagner, D. E. *et al.* Single-cell mapping of gene expression landscapes and lineage in the zebrafish embryo. *Science* **360**, 981-+, doi:10.1126/science.aar4362 (2018).

Supp. Table 1. References for previously published single-cell RNA sequencing datasets from *Mus musculus* (mouse) and *Danio rerio* (zebrafish) used in this study.

Stage	Reference	Data Link
Mouse		
E3.5, E4.5, E5.5, E6.5	Mohammed et al. (2017)[16]	https://tome.gs.washington.edu/
E5.25, E5.5, E6.25, E6.5,	Cheng et al. (2019)[17]	
E6.5-E8.5	Pijuan-Sala et al. (2019)[18]	
E8.5	Qiu et al. (2022)[1]	
E9.5-E13.5	Cao et al. (2019)[19]	
Zebrafish		
hpf3.3-hpf12	Farrell et al. (2018)[20]	https://tome.gs.washington.edu/
hpf6, hpf8, hpf10, hpf14-hpf24	Wagner et al. (2018)[21]	
hpf36, hpf48	Sur et al. (2023)[2]	https://daniocell.nichd.nih.gov/ (metadata information) https://www.ncbi.nlm.nih.gov/geo/query/acc.cgi?acc=GSE223922

Supplementary Figures

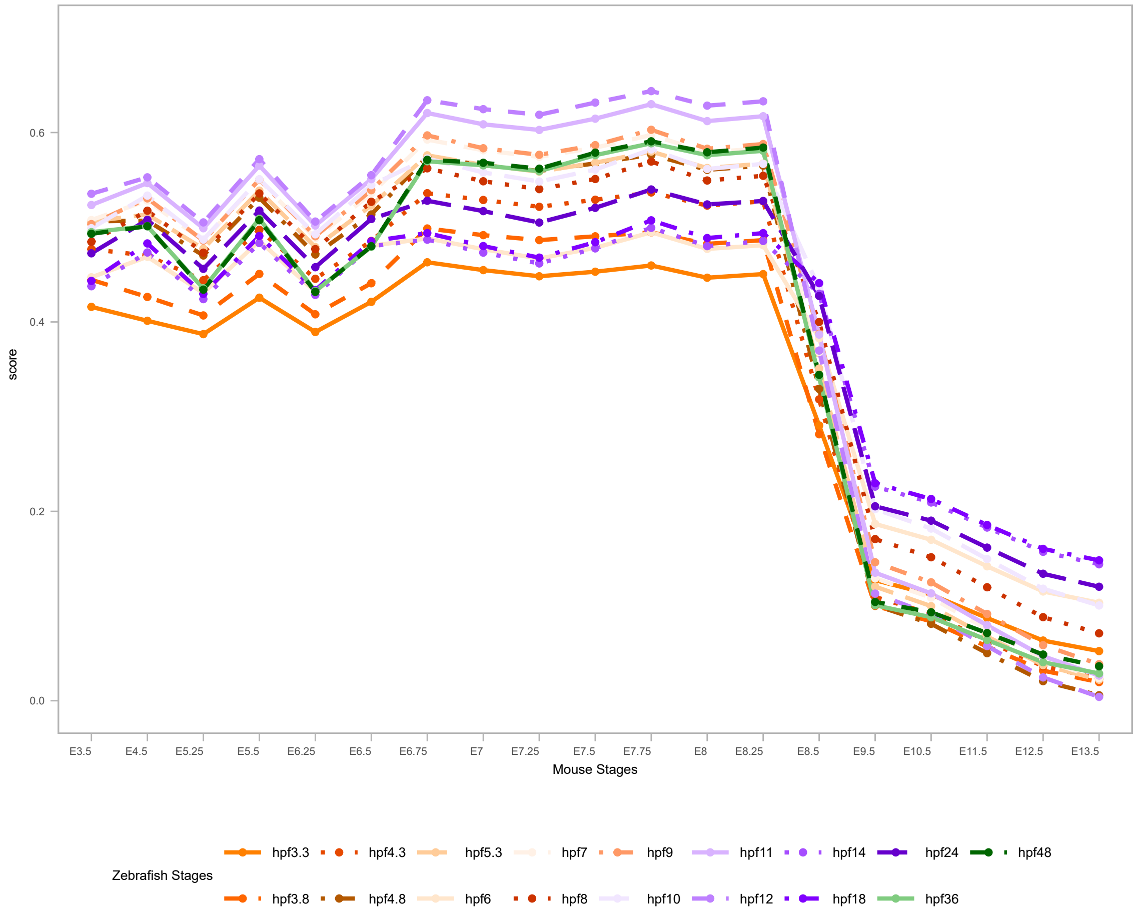

Supp. Fig. 1. Transcriptome similarity between mouse and zebrafish embryos. Transcriptome similarity was quantified using Spearman's correlation coefficient (ρ). Each line represents a zebrafish developmental stage, projected against its similarity to mouse stages (x-axis). The results reveal an hourglass-like pattern, with the highest similarities observed during mid-embryogenesis, while early vs. early and late vs. late stage comparisons show lower similarity.

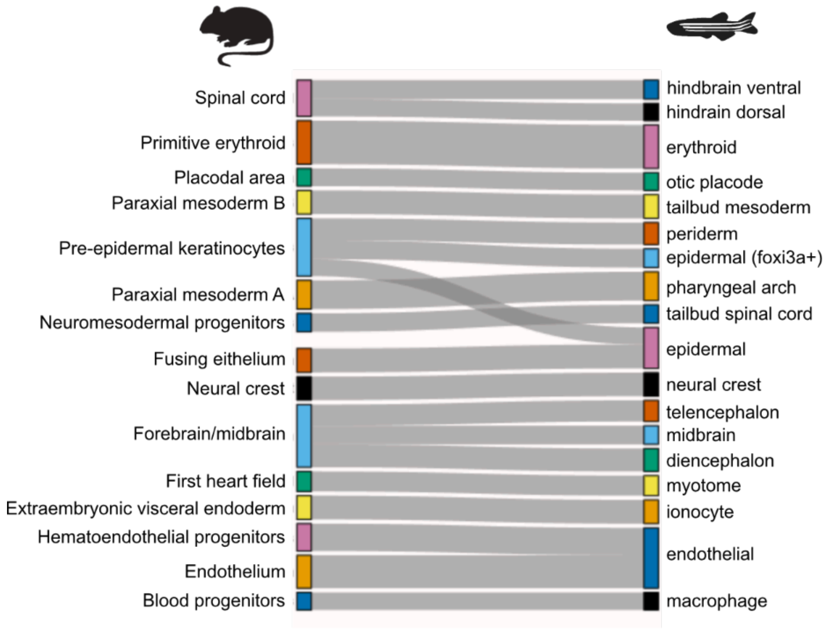

Supp. Fig. 2. Sankey plot illustrating homologous cell states between mouse and zebrafish, as identified through SAMap analysis. The width of the connections represents the degree of similarity between matched cell states. Mouse and zebrafish silhouettes obtained from PhyloPic (https://www.phylopic.org); image credit Soledad Miranda-Rottmann and Ian Quigley, respectively (CC BY 3.0).

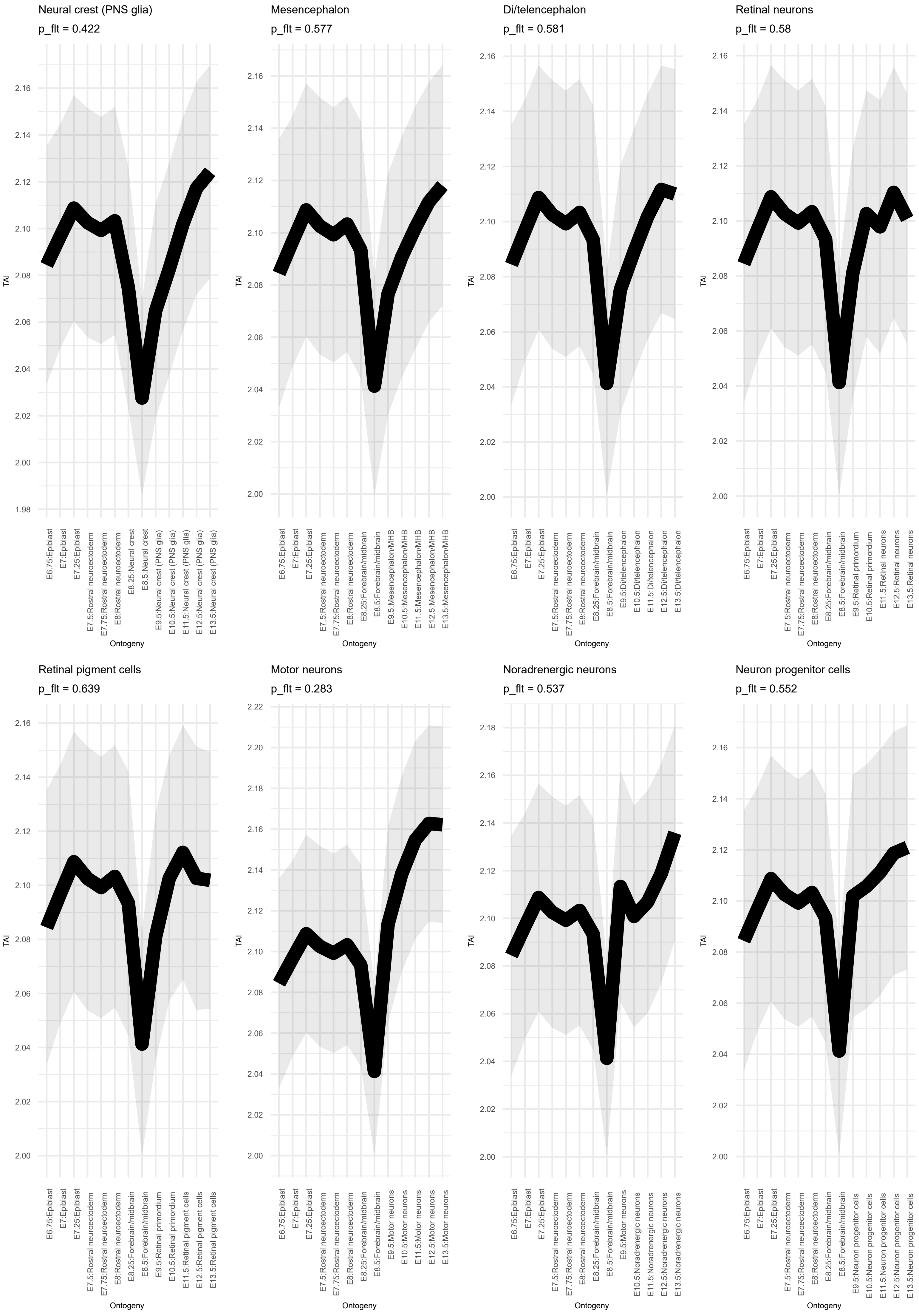
Neural crest (PNS glia)
p_flt = 0.422
Mesencephalon
p_flt = 0.577
Di/telencephalon
p_flt = 0.581
Retinal neurons
p_flt = 0.58
Retinal pigment cells
p_flt = 0.639
Motor neurons
p_flt = 0.283
Noradrenergic neurons
p_flt = 0.537
Neuron progenitor cells
p_flt = 0.552
TAI
Ontogeny

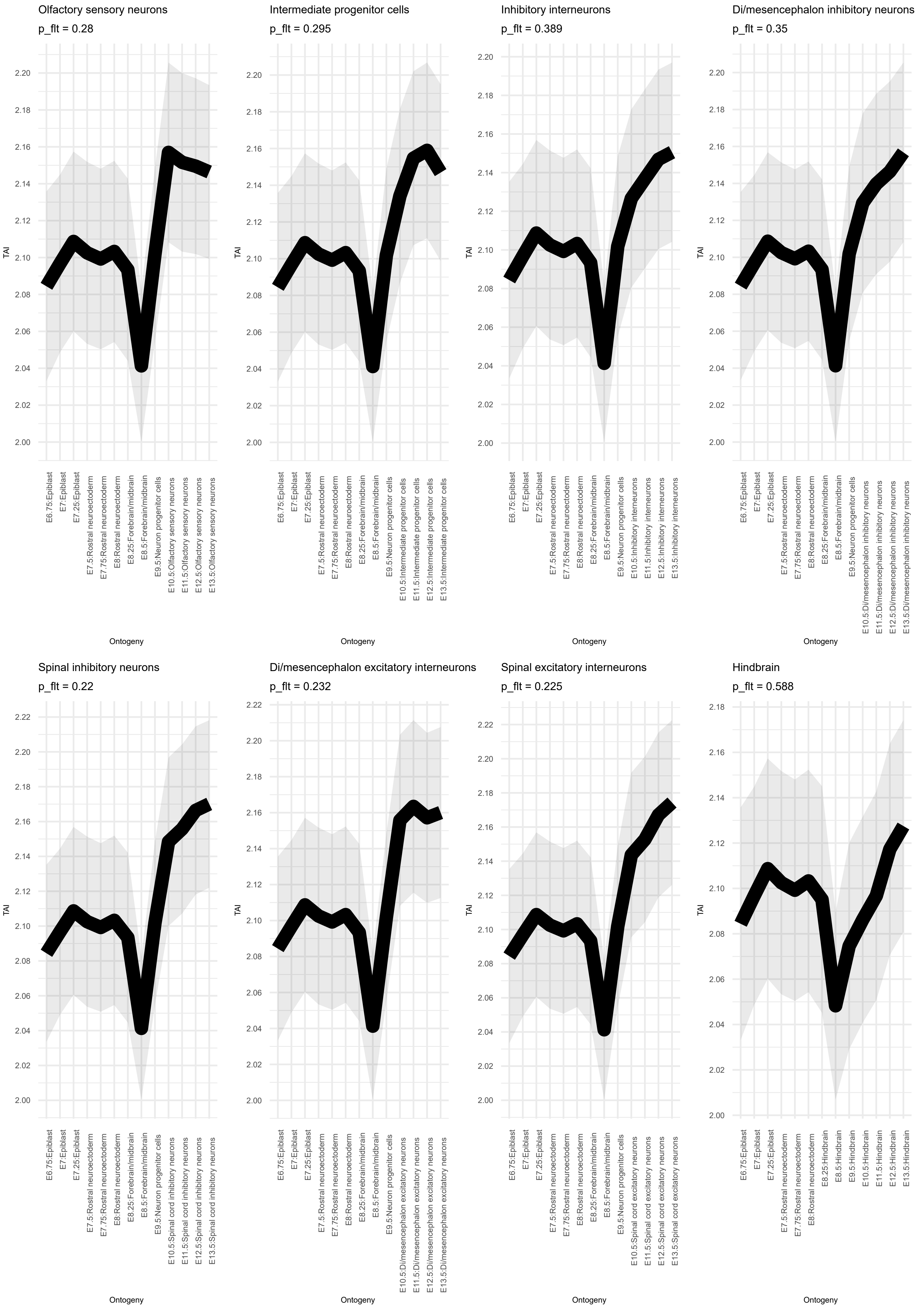
Olfactory sensory neurons
p_flt = 0.28
TAI
2.20 2.18 2.16 2.14 2.12 2.10 2.08 2.06 2.04 2.02 2.00
E6.75:Epiblast
E7:Epiblast
E7.25:Epiblast
E7.5:Rostral neuroectoderm
E7.75:Rostral neuroectoderm
E8:Rostral neuroectoderm
E8.25:Forebrain/midbrain
E8.5:Forebrain/midbrain
E9.5:Neuron progenitor cells
E10.5:Olfactory sensory neurons
E11.5:Olfactory sensory neurons
E12.5:Olfactory sensory neurons
E13.5:Olfactory sensory neurons
Ontogeny
Intermediate progenitor cells
p_flt = 0.295
TAI
2.20 2.18 2.16 2.14 2.12 2.10 2.08 2.06 2.04 2.02 2.00
E6.75:Epiblast
E7:Epiblast
E7.25:Epiblast
E7.5:Rostral neuroectoderm
E7.75:Rostral neuroectoderm
E8:Rostral neuroectoderm
E8.25:Forebrain/midbrain
E8.5:Forebrain/midbrain
E9.5:Neuron progenitor cells
E10.5:Intermediate progenitor cells
E11.5:Intermediate progenitor cells
E12.5:Intermediate progenitor cells
E13.5:Intermediate progenitor cells
Ontogeny
Inhibitory interneurons
p_flt = 0.389
TAI
2.20 2.18 2.16 2.14 2.12 2.10 2.08 2.06 2.04 2.02 2.00
E6.75:Epiblast
E7:Epiblast
E7.25:Epiblast
E7.5:Rostral neuroectoderm
E7.75:Rostral neuroectoderm
E8:Rostral neuroectoderm
E8.25:Forebrain/midbrain
E8.5:Forebrain/midbrain
E9.5:Neuron progenitor cells
E10.5:Inhibitory interneurons
E11.5:Inhibitory interneurons
E12.5:Inhibitory interneurons
E13.5:Inhibitory interneurons
Ontogeny
Di/mesencephalon inhibitory neurons
p_flt = 0.35
TAI
2.20 2.18 2.16 2.14 2.12 2.10 2.08 2.06 2.04 2.02 2.00
E6.75:Epiblast
E7:Epiblast
E7.25:Epiblast
E7.5:Rostral neuroectoderm
E7.75:Rostral neuroectoderm
E8:Rostral neuroectoderm
E8.25:Forebrain/midbrain
E8.5:Forebrain/midbrain
E9.5:Neuron progenitor cells
E10.5:Di/mesencephalon inhibitory neurons
E11.5:Di/mesencephalon inhibitory neurons
E12.5:Di/mesencephalon inhibitory neurons
E13.5:Di/mesencephalon inhibitory neurons
Ontogeny
Spinal inhibitory neurons
p_flt = 0.22
TAI
2.22 2.20 2.18 2.16 2.14 2.12 2.10 2.08 2.06 2.04 2.02 2.00
E6.75:Epiblast
E7:Epiblast
E7.25:Epiblast
E7.5:Rostral neuroectoderm
E7.75:Rostral neuroectoderm
E8:Rostral neuroectoderm
E8.25:Forebrain/midbrain
E8.5:Forebrain/midbrain
E9.5:Neuron progenitor cells
E10.5:Spinal cord inhibitory neurons
E11.5:Spinal cord inhibitory neurons
E12.5:Spinal cord inhibitory neurons
E13.5:Spinal cord inhibitory neurons
Ontogeny
Di/mesencephalon excitatory interneurons
p_flt = 0.232
TAI
2.22 2.20 2.18 2.16 2.14 2.12 2.10 2.08 2.06 2.04 2.02 2.00
E6.75:Epiblast
E7:Epiblast
E7.25:Epiblast
E7.5:Rostral neuroectoderm
E7.75:Rostral neuroectoderm
E8:Rostral neuroectoderm
E8.25:Forebrain/midbrain
E8.5:Forebrain/midbrain
E9.5:Neuron progenitor cells
E10.5:Di/mesencephalon excitatory neurons
E11.5:Di/mesencephalon excitatory neurons
E12.5:Di/mesencephalon excitatory neurons
E13.5:Di/mesencephalon excitatory neurons
Ontogeny
Spinal excitatory interneurons
p_flt = 0.225
TAI
2.22 2.20 2.18 2.16 2.14 2.12 2.10 2.08 2.06 2.04 2.02 2.00
E6.75:Epiblast
E7:Epiblast
E7.25:Epiblast
E7.5:Rostral neuroectoderm
E7.75:Rostral neuroectoderm
E8:Rostral neuroectoderm
E8.25:Forebrain/midbrain
E8.5:Forebrain/midbrain
E9.5:Neuron progenitor cells
E10.5:Spinal cord excitatory neurons
E11.5:Spinal cord excitatory neurons
E12.5:Spinal cord excitatory neurons
E13.5:Spinal cord excitatory neurons
Ontogeny
Hindbrain
p_flt = 0.588
TAI
2.18 2.16 2.14 2.12 2.10 2.08 2.06 2.04 2.02 2.00
E6.75:Epiblast
E7:Epiblast
E7.25:Epiblast
E7.5:Rostral neuroectoderm
E7.75:Rostral neuroectoderm
E8:Rostral neuroectoderm
E8.25:Hindbrain
E8.5:Hindbrain
E9.5:Hindbrain
E10.5:Hindbrain
E11.5:Hindbrain
E12.5:Hindbrain
E13.5:Hindbrain
Ontogeny

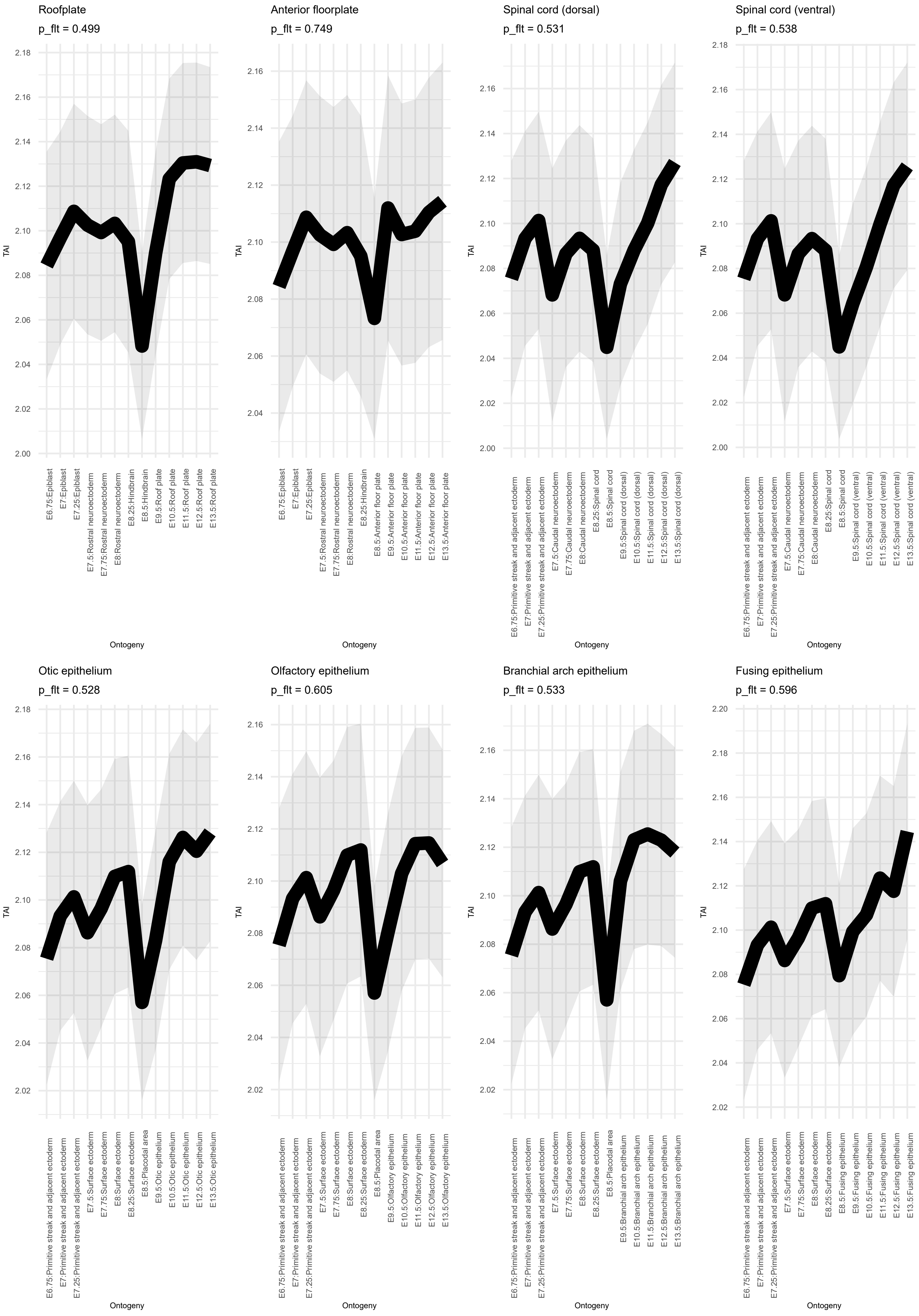
Roofplate
p_flt = 0.499
TAI
2.00 2.02 2.04 2.06 2.08 2.10 2.12 2.14 2.16 2.18
E6.75:Epiblast
E7:Epiblast
E7.25:Epiblast
E7.5:Rostral neuroectoderm
E7.75:Rostral neuroectoderm
E8:Rostral neuroectoderm
E8.25:Hindbrain
E8.5:Hindbrain
E9.5:Roof plate
E10.5:Roof plate
E11.5:Roof plate
E12.5:Roof plate
E13.5:Roof plate
Ontogeny
Anterior floorplate
p_flt = 0.749
TAI
2.04 2.06 2.08 2.10 2.12 2.14 2.16
E6.75:Epiblast
E7:Epiblast
E7.25:Epiblast
E7.5:Rostral neuroectoderm
E7.75:Rostral neuroectoderm
E8:Rostral neuroectoderm
E8.25:Hindbrain
E8.5:Anterior floor plate
E9.5:Anterior floor plate
E10.5:Anterior floor plate
E11.5:Anterior floor plate
E12.5:Anterior floor plate
E13.5:Anterior floor plate
Ontogeny
Spinal cord (dorsal)
p_flt = 0.531
TAI
2.00 2.02 2.04 2.06 2.08 2.10 2.12 2.14 2.16
E6.75:Primitive streak and adjacent ectoderm
E7:Primitive streak and adjacent ectoderm
E7.25:Primitive streak and adjacent ectoderm
E7.5:Caudal neuroectoderm
E7.75:Caudal neuroectoderm
E8:Caudal neuroectoderm
E8.25:Spinal cord
E8.5:Spinal cord
E9.5:Spinal cord (dorsal)
E10.5:Spinal cord (dorsal)
E11.5:Spinal cord (dorsal)
E12.5:Spinal cord (dorsal)
E13.5:Spinal cord (dorsal)
Ontogeny
Spinal cord (ventral)
p_flt = 0.538
TAI
2.00 2.02 2.04 2.06 2.08 2.10 2.12 2.14 2.16 2.18
E6.75:Primitive streak and adjacent ectoderm
E7:Primitive streak and adjacent ectoderm
E7.25:Primitive streak and adjacent ectoderm
E7.5:Caudal neuroectoderm
E7.75:Caudal neuroectoderm
E8:Caudal neuroectoderm
E8.25:Spinal cord
E8.5:Spinal cord
E9.5:Spinal cord (ventral)
E10.5:Spinal cord (ventral)
E11.5:Spinal cord (ventral)
E12.5:Spinal cord (ventral)
E13.5:Spinal cord (ventral)
Ontogeny
Otic epithelium
p_flt = 0.528
TAI
2.02 2.04 2.06 2.08 2.10 2.12 2.14 2.16 2.18
E6.75:Primitive streak and adjacent ectoderm
E7:Primitive streak and adjacent ectoderm
E7.25:Primitive streak and adjacent ectoderm
E7.5:Surface ectoderm
E7.75:Surface ectoderm
E8:Surface ectoderm
E8.25:Surface ectoderm
E8.5:Placodal area
E9.5:Otic epithelium
E10.5:Otic epithelium
E11.5:Otic epithelium
E12.5:Otic epithelium
E13.5:Otic epithelium
Ontogeny
Olfactory epithelium
p_flt = 0.605
TAI
2.02 2.04 2.06 2.08 2.10 2.12 2.14 2.16
E6.75:Primitive streak and adjacent ectoderm
E7:Primitive streak and adjacent ectoderm
E7.25:Primitive streak and adjacent ectoderm
E7.5:Surface ectoderm
E7.75:Surface ectoderm
E8:Surface ectoderm
E8.25:Surface ectoderm
E8.5:Placodal area
E9.5:Olfactory epithelium
E10.5:Olfactory epithelium
E11.5:Olfactory epithelium
E12.5:Olfactory epithelium
E13.5:Olfactory epithelium
Ontogeny
Branchial arch epithelium
p_flt = 0.533
TAI
2.02 2.04 2.06 2.08 2.10 2.12 2.14 2.16
E6.75:Primitive streak and adjacent ectoderm
E7:Primitive streak and adjacent ectoderm
E7.25:Primitive streak and adjacent ectoderm
E7.5:Surface ectoderm
E7.75:Surface ectoderm
E8:Surface ectoderm
E8.25:Surface ectoderm
E8.5:Placodal area
E9.5:Branchial arch epithelium
E10.5:Branchial arch epithelium
E11.5:Branchial arch epithelium
E12.5:Branchial arch epithelium
E13.5:Branchial arch epithelium
Ontogeny
Fusing epithelium
p_flt = 0.596
TAI
2.02 2.04 2.06 2.08 2.10 2.12 2.14 2.16 2.18 2.20
E6.75:Primitive streak and adjacent ectoderm
E7:Primitive streak and adjacent ectoderm
E7.25:Primitive streak and adjacent ectoderm
E7.5:Surface ectoderm
E7.75:Surface ectoderm
E8:Surface ectoderm
E8.25:Surface ectoderm
E8.5:Fusing epithelium
E9.5:Fusing epithelium
E10.5:Fusing epithelium
E11.5:Fusing epithelium
E12.5:Fusing epithelium
E13.5:Fusing epithelium
Ontogeny

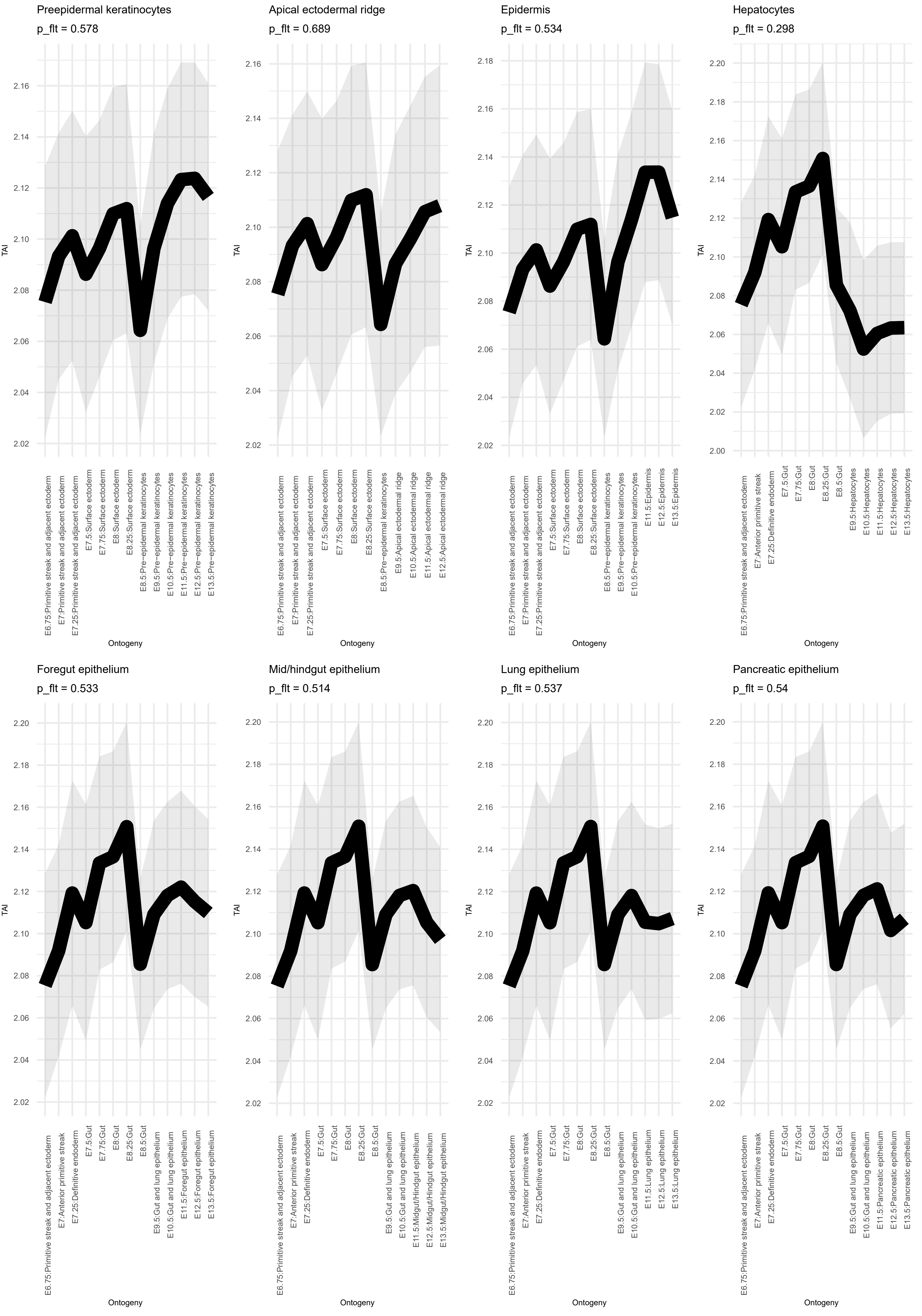

Preepidermal keratinocytes
p_flt = 0.578
2.02
2.04
2.06
2.08
2.10
2.12
2.14
2.16
TAI
E6.75:Primitive streak and adjacent ectoderm
E7:Primitive streak and adjacent ectoderm
E7.25:Primitive streak and adjacent ectoderm
E7.5:Surface ectoderm
E7.75:Surface ectoderm
E8:Surface ectoderm
E8.25:Surface ectoderm
E8.5:Pre−epidermal keratinocytes
E9.5:Pre−epidermal keratinocytes
E10.5:Pre−epidermal keratinocytes
E11.5:Pre−epidermal keratinocytes
E12.5:Pre−epidermal keratinocytes
E13.5:Pre−epidermal keratinocytes
Ontogeny
Apical ectodermal ridge
p_flt = 0.689
2.02
2.04
2.06
2.08
2.10
2.12
2.14
2.16
TAI
E6.75:Primitive streak and adjacent ectoderm
E7:Primitive streak and adjacent ectoderm
E7.25:Primitive streak and adjacent ectoderm
E7.5:Surface ectoderm
E7.75:Surface ectoderm
E8:Surface ectoderm
E8.25:Surface ectoderm
E8.5:Pre−epidermal keratinocytes
E9.5:Apical ectodermal ridge
E10.5:Apical ectodermal ridge
E11.5:Apical ectodermal ridge
E12.5:Apical ectodermal ridge
Ontogeny
Epidermis
p_flt = 0.534
2.02
2.04
2.06
2.08
2.10
2.12
2.14
2.16
2.18
TAI
E6.75:Primitive streak and adjacent ectoderm
E7:Primitive streak and adjacent ectoderm
E7.25:Primitive streak and adjacent ectoderm
E7.5:Surface ectoderm
E7.75:Surface ectoderm
E8:Surface ectoderm
E8.25:Surface ectoderm
E8.5:Pre−epidermal keratinocytes
E9.5:Pre−epidermal keratinocytes
E10.5:Pre−epidermal keratinocytes
E11.5:Epidermis
E12.5:Epidermis
E13.5:Epidermis
Ontogeny
Hepatocytes
p_flt = 0.298
2.00
2.02
2.04
2.06
2.08
2.10
2.12
2.14
2.16
2.18
2.20
TAI
E6.75:Primitive streak and adjacent ectoderm
E7:Anterior primitive streak
E7.25:Definitive endoderm
E7.5:Gut
E7.75:Gut
E8:Gut
E8.25:Gut
E8.5:Gut
E9.5:Hepatocytes
E10.5:Hepatocytes
E11.5:Hepatocytes
E12.5:Hepatocytes
E13.5:Hepatocytes
Ontogeny
Foregut epithelium
p_flt = 0.533
2.02
2.04
2.06
2.08
2.10
2.12
2.14
2.16
2.18
2.20
TAI
E6.75:Primitive streak and adjacent ectoderm
E7:Anterior primitive streak
E7.25:Definitive endoderm
E7.5:Gut
E7.75:Gut
E8:Gut
E8.25:Gut
E8.5:Gut
E9.5:Gut and lung epithelium
E10.5:Gut and lung epithelium
E11.5:Foregut epithelium
E12.5:Foregut epithelium
E13.5:Foregut epithelium
Ontogeny
Mid/hindgut epithelium
p_flt = 0.514
2.02
2.04
2.06
2.08
2.10
2.12
2.14
2.16
2.18
2.20
TAI
E6.75:Primitive streak and adjacent ectoderm
E7:Anterior primitive streak
E7.25:Definitive endoderm
E7.5:Gut
E7.75:Gut
E8:Gut
E8.25:Gut
E8.5:Gut
E9.5:Gut and lung epithelium
E10.5:Gut and lung epithelium
E11.5:Midgut/Hindgut epithelium
E12.5:Midgut/Hindgut epithelium
E13.5:Midgut/Hindgut epithelium
Ontogeny
Lung epithelium
p_flt = 0.537
2.02
2.04
2.06
2.08
2.10
2.12
2.14
2.16
2.18
2.20
TAI
E6.75:Primitive streak and adjacent ectoderm
E7:Anterior primitive streak
E7.25:Definitive endoderm
E7.5:Gut
E7.75:Gut
E8:Gut
E8.25:Gut
E8.5:Gut
E9.5:Gut and lung epithelium
E10.5:Gut and lung epithelium
E11.5:Lung epithelium
E12.5:Lung epithelium
E13.5:Lung epithelium
Ontogeny
Pancreatic epithelium
p_flt = 0.54
2.02
2.04
2.06
2.08
2.10
2.12
2.14
2.16
2.18
2.20
TAI
E6.75:Primitive streak and adjacent ectoderm
E7:Anterior primitive streak
E7.25:Definitive endoderm
E7.5:Gut
E7.75:Gut
E8:Gut
E8.25:Gut
E8.5:Gut
E9.5:Gut and lung epithelium
E10.5:Gut and lung epithelium
E11.5:Pancreatic epithelium
E12.5:Pancreatic epithelium
E13.5:Pancreatic epithelium
Ontogeny

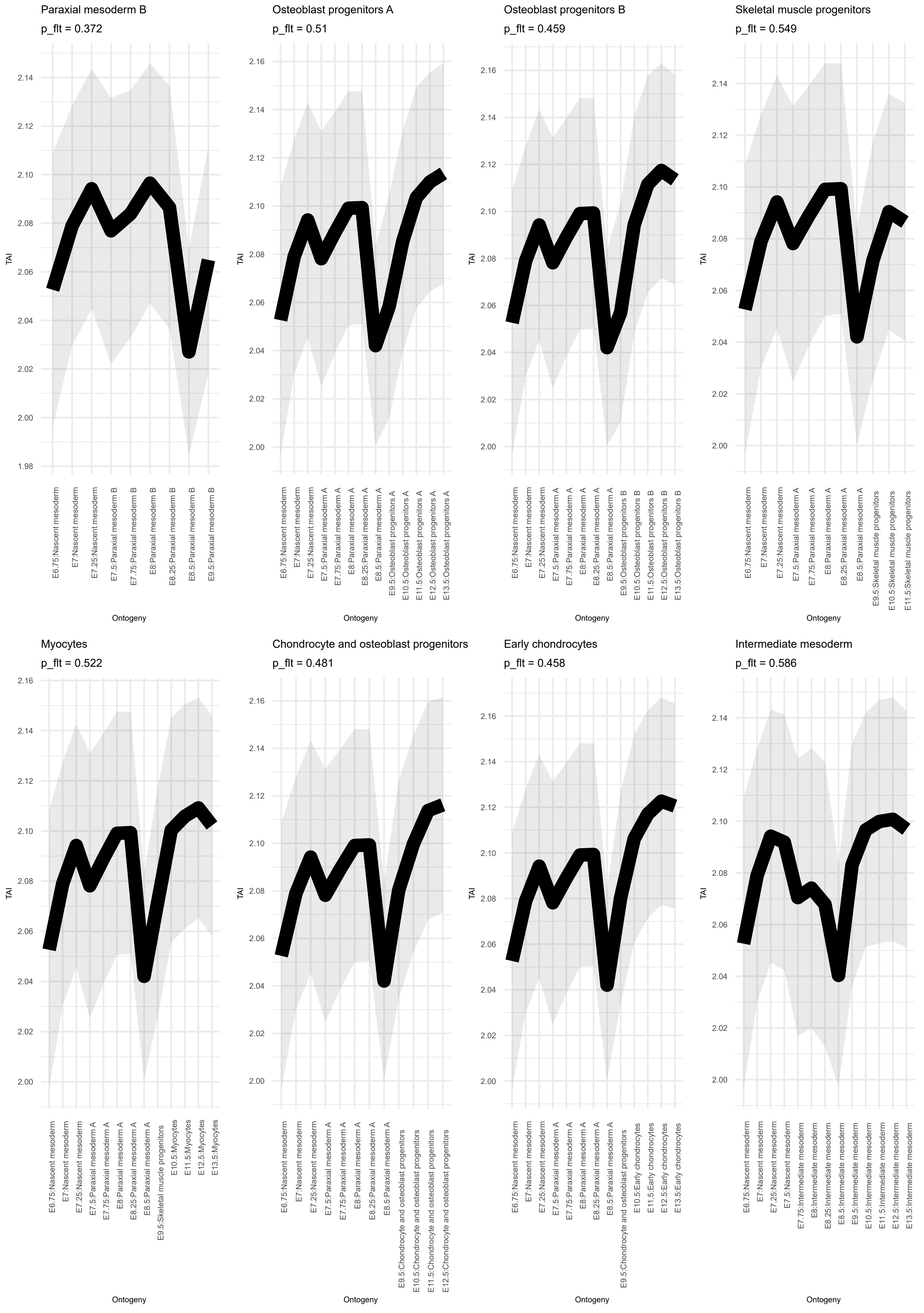
Paraxial mesoderm B
p_flt = 0.372
TAI
Ontogeny
E6.75:Nascent mesoderm
E7:Nascent mesoderm
E7.25:Nascent mesoderm
E7.5:Paraxial mesoderm B
E7.75:Paraxial mesoderm B
E8:Paraxial mesoderm B
E8.25:Paraxial mesoderm B
E8.5:Paraxial mesoderm B
E9.5:Paraxial mesoderm B
Osteoblast progenitors A
p_flt = 0.51
E7.5:Paraxial mesoderm A
E7.75:Paraxial mesoderm A
E8:Paraxial mesoderm A
E8.25:Paraxial mesoderm A
E8.5:Paraxial mesoderm A
E9.5:Osteoblast progenitors A
E10.5:Osteoblast progenitors A
E11.5:Osteoblast progenitors A
E12.5:Osteoblast progenitors A
E13.5:Osteoblast progenitors A
Osteoblast progenitors B
p_flt = 0.459
E9.5:Osteoblast progenitors B
E10.5:Osteoblast progenitors B
E11.5:Osteoblast progenitors B
E12.5:Osteoblast progenitors B
E13.5:Osteoblast progenitors B
Skeletal muscle progenitors
p_flt = 0.549
E9.5:Skeletal muscle progenitors
E10.5:Skeletal muscle progenitors
E11.5:Skeletal muscle progenitors
Myocytes
p_flt = 0.522
E10.5:Myocytes
E11.5:Myocytes
E12.5:Myocytes
E13.5:Myocytes
Chondrocyte and osteoblast progenitors
p_flt = 0.481
E9.5:Chondrocyte and osteoblast progenitors
E10.5:Chondrocyte and osteoblast progenitors
E11.5:Chondrocyte and osteoblast progenitors
E12.5:Chondrocyte and osteoblast progenitors
Early chondrocytes
p_flt = 0.458
E10.5:Early chondrocytes
E11.5:Early chondrocytes
E12.5:Early chondrocytes
E13.5:Early chondrocytes
Intermediate mesoderm
p_flt = 0.586
E7.75:Intermediate mesoderm
E8:Intermediate mesoderm
E8.25:Intermediate mesoderm
E8.5:Intermediate mesoderm
E9.5:Intermediate mesoderm
E10.5:Intermediate mesoderm
E11.5:Intermediate mesoderm
E12.5:Intermediate mesoderm
E13.5:Intermediate mesoderm

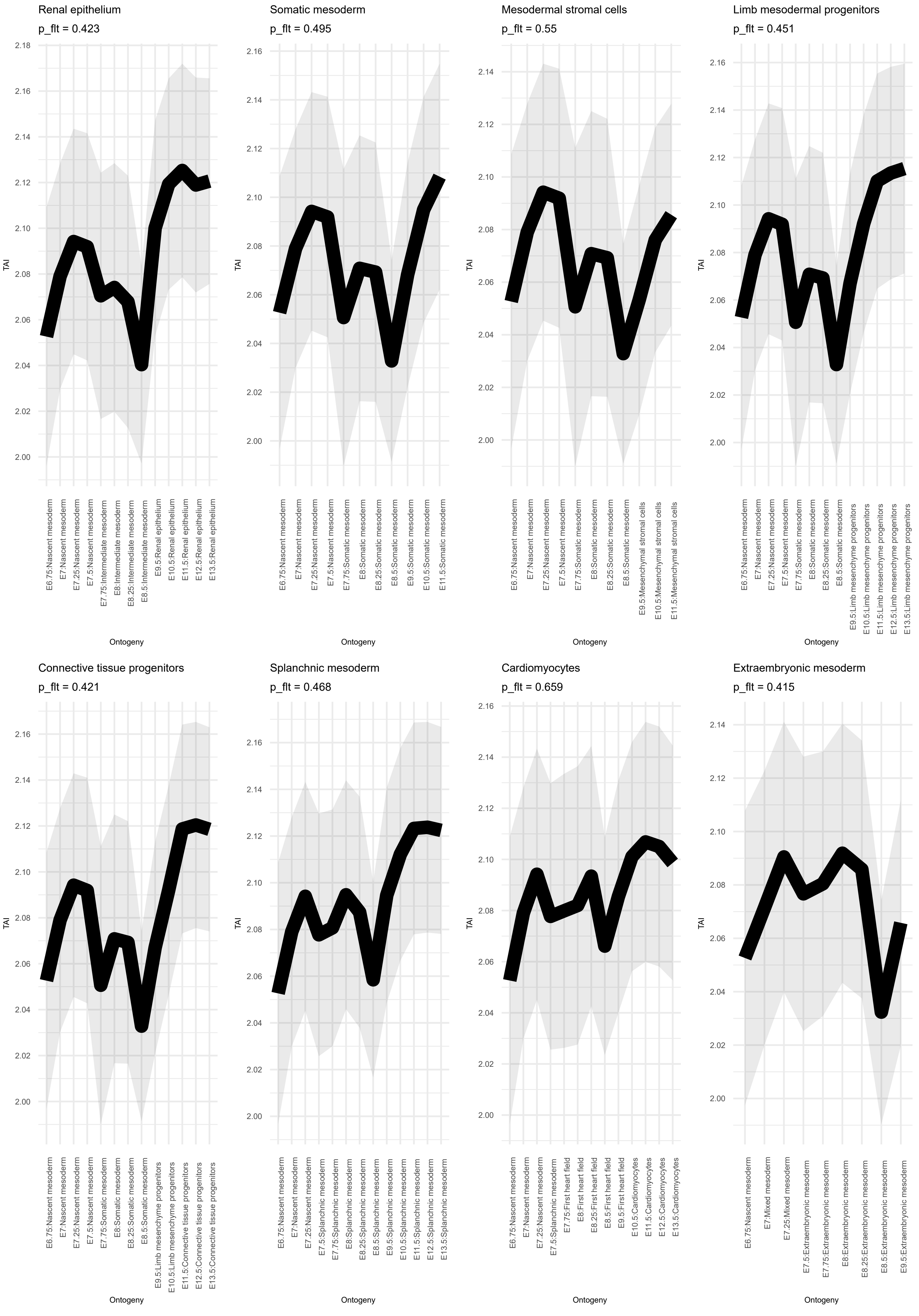

Renal epithelium
p_flt = 0.423
TAI
E6.75:Nascent mesoderm
E7:Nascent mesoderm
E7.25:Nascent mesoderm
E7.5:Nascent mesoderm
E7.75:Intermediate mesoderm
E8:Intermediate mesoderm
E8.25:Intermediate mesoderm
E8.5:Intermediate mesoderm
E9.5:Renal epithelium
E10.5:Renal epithelium
E11.5:Renal epithelium
E12.5:Renal epithelium
E13.5:Renal epithelium
Ontogeny
Somatic mesoderm
p_flt = 0.495
TAI
E6.75:Nascent mesoderm
E7:Nascent mesoderm
E7.25:Nascent mesoderm
E7.5:Nascent mesoderm
E7.75:Somatic mesoderm
E8:Somatic mesoderm
E8.25:Somatic mesoderm
E8.5:Somatic mesoderm
E9.5:Somatic mesoderm
E10.5:Somatic mesoderm
E11.5:Somatic mesoderm
Ontogeny
Mesodermal stromal cells
p_flt = 0.55
TAI
E6.75:Nascent mesoderm
E7:Nascent mesoderm
E7.25:Nascent mesoderm
E7.5:Nascent mesoderm
E7.75:Somatic mesoderm
E8:Somatic mesoderm
E8.25:Somatic mesoderm
E8.5:Somatic mesoderm
E9.5:Mesenchymal stromal cells
E10.5:Mesenchymal stromal cells
E11.5:Mesenchymal stromal cells
Ontogeny
Limb mesodermal progenitors
p_flt = 0.451
TAI
E6.75:Nascent mesoderm
E7:Nascent mesoderm
E7.25:Nascent mesoderm
E7.5:Nascent mesoderm
E7.75:Somatic mesoderm
E8:Somatic mesoderm
E8.25:Somatic mesoderm
E8.5:Somatic mesoderm
E9.5:Limb mesenchyme progenitors
E10.5:Limb mesenchyme progenitors
E11.5:Limb mesenchyme progenitors
E12.5:Limb mesenchyme progenitors
E13.5:Limb mesenchyme progenitors
Ontogeny
Connective tissue progenitors
p_flt = 0.421
TAI
E6.75:Nascent mesoderm
E7:Nascent mesoderm
E7.25:Nascent mesoderm
E7.5:Nascent mesoderm
E7.75:Somatic mesoderm
E8:Somatic mesoderm
E8.25:Somatic mesoderm
E8.5:Somatic mesoderm
E9.5:Limb mesenchyme progenitors
E10.5:Limb mesenchyme progenitors
E11.5:Connective tissue progenitors
E12.5:Connective tissue progenitors
E13.5:Connective tissue progenitors
Ontogeny
Splanchnic mesoderm
p_flt = 0.468
TAI
E6.75:Nascent mesoderm
E7:Nascent mesoderm
E7.25:Nascent mesoderm
E7.5:Splanchnic mesoderm
E7.75:Splanchnic mesoderm
E8:Splanchnic mesoderm
E8.25:Splanchnic mesoderm
E8.5:Splanchnic mesoderm
E9.5:Splanchnic mesoderm
E10.5:Splanchnic mesoderm
E11.5:Splanchnic mesoderm
E12.5:Splanchnic mesoderm
E13.5:Splanchnic mesoderm
Ontogeny
Cardiomyocytes
p_flt = 0.659
TAI
E6.75:Nascent mesoderm
E7:Nascent mesoderm
E7.25:Nascent mesoderm
E7.5:Splanchnic mesoderm
E7.75:First heart field
E8:First heart field
E8.25:First heart field
E8.5:First heart field
E9.5:First heart field
E10.5:Cardiomyocytes
E11.5:Cardiomyocytes
E12.5:Cardiomyocytes
E13.5:Cardiomyocytes
Ontogeny
Extraembryonic mesoderm
p_flt = 0.415
TAI
E6.75:Nascent mesoderm
E7:Mixed mesoderm
E7.25:Mixed mesoderm
E7.5:Extraembryonic mesoderm
E7.75:Extraembryonic mesoderm
E8:Extraembryonic mesoderm
E8.25:Extraembryonic mesoderm
E8.5:Extraembryonic mesoderm
E9.5:Extraembryonic mesoderm
Ontogeny

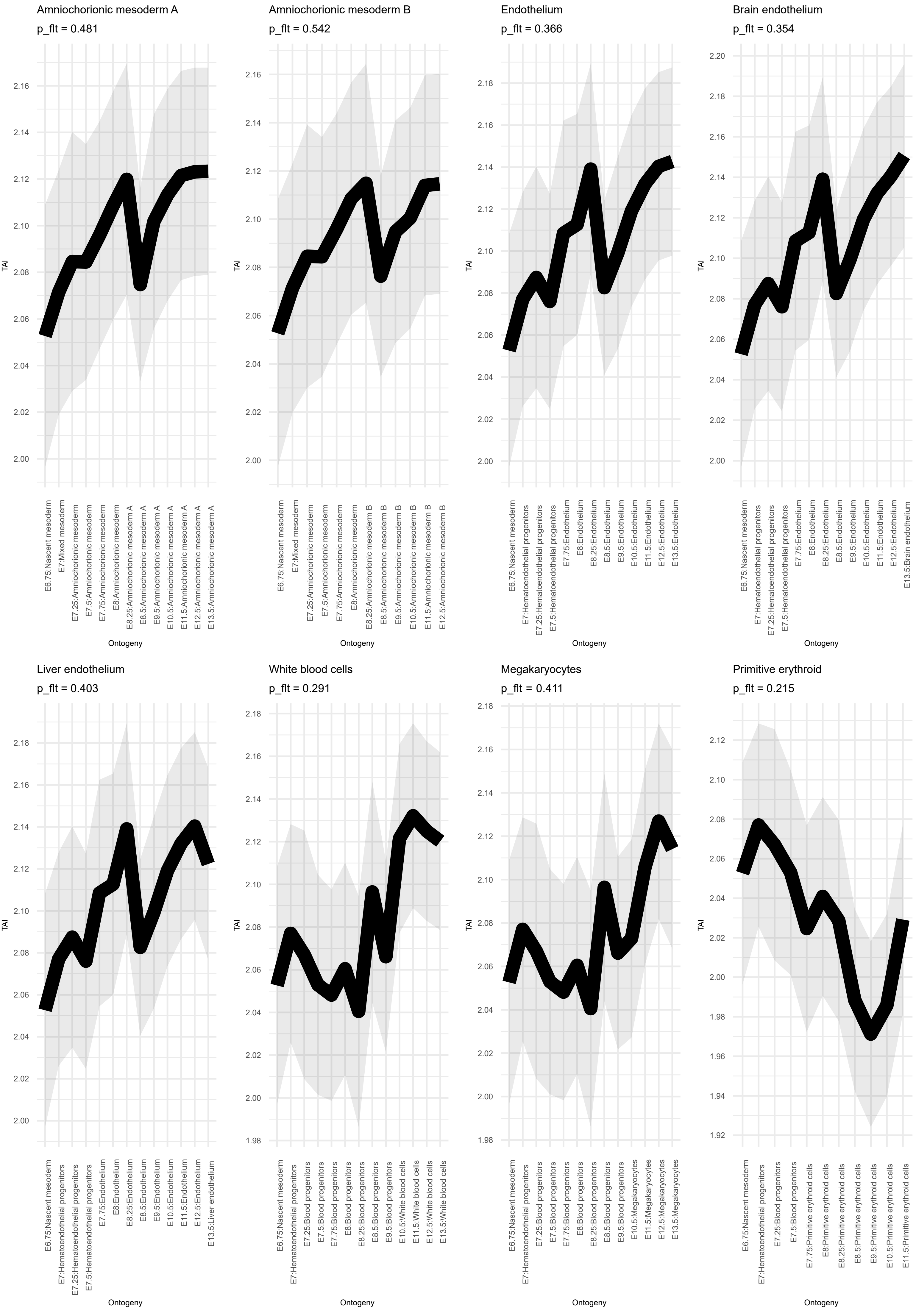

Amniochorionic mesoderm A
p_flt = 0.481
Amniochorionic mesoderm B
p_flt = 0.542
Endothelium
p_flt = 0.366
Brain endothelium
p_flt = 0.354
Liver endothelium
p_flt = 0.403
White blood cells
p_flt = 0.291
Megakaryocytes
p_flt = 0.411
Primitive erythroid
p_flt = 0.215
TAI
Ontogeny
E6.75:Nascent mesoderm
E7:Mixed mesoderm
E7.25:Amniochorionic mesoderm
E7.5:Amniochorionic mesoderm
E7.75:Amniochorionic mesoderm
E8:Amniochorionic mesoderm
E8.25:Amniochorionic mesoderm A
E8.5:Amniochorionic mesoderm A
E9.5:Amniochorionic mesoderm A
E10.5:Amniochorionic mesoderm A
E11.5:Amniochorionic mesoderm A
E12.5:Amniochorionic mesoderm A
E13.5:Amniochorionic mesoderm A
E8.25:Amniochorionic mesoderm B
E8.5:Amniochorionic mesoderm B
E9.5:Amniochorionic mesoderm B
E10.5:Amniochorionic mesoderm B
E11.5:Amniochorionic mesoderm B
E12.5:Amniochorionic mesoderm B
E7:Hematoendothelial progenitors
E7.25:Hematoendothelial progenitors
E7.5:Hematoendothelial progenitors
E7.75:Endothelium
E8:Endothelium
E8.25:Endothelium
E8.5:Endothelium
E9.5:Endothelium
E10.5:Endothelium
E11.5:Endothelium
E12.5:Endothelium
E13.5:Endothelium
E13.5:Brain endothelium
E13.5:Liver endothelium
E7.25:Blood progenitors
E7.5:Blood progenitors
E7.75:Blood progenitors
E8:Blood progenitors
E8.25:Blood progenitors
E8.5:Blood progenitors
E9.5:Blood progenitors
E10.5:White blood cells
E11.5:White blood cells
E12.5:White blood cells
E13.5:White blood cells
E10.5:Megakaryocytes
E11.5:Megakaryocytes
E12.5:Megakaryocytes
E13.5:Megakaryocytes
E7.75:Primitive erythroid cells
E8:Primitive erythroid cells
E8.25:Primitive erythroid cells
E8.5:Primitive erythroid cells
E9.5:Primitive erythroid cells
E10.5:Primitive erythroid cells
E11.5:Primitive erythroid cells

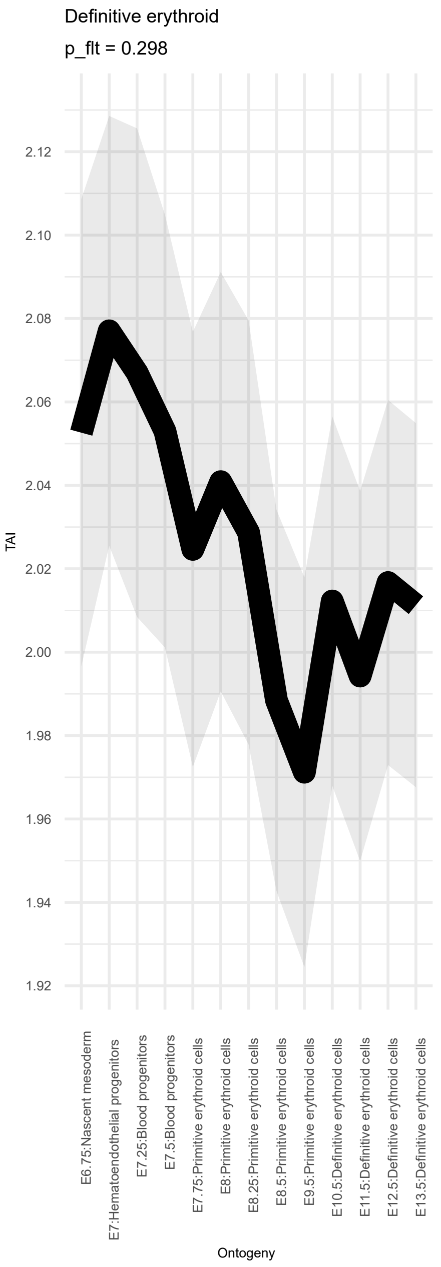

Supp. Fig. 3. Flatline tests for the TAI profile of individual mouse cellular trajectories. Significance was assessed using 10,000 permutations, with a p-value ≤ 0.05 considered significant.

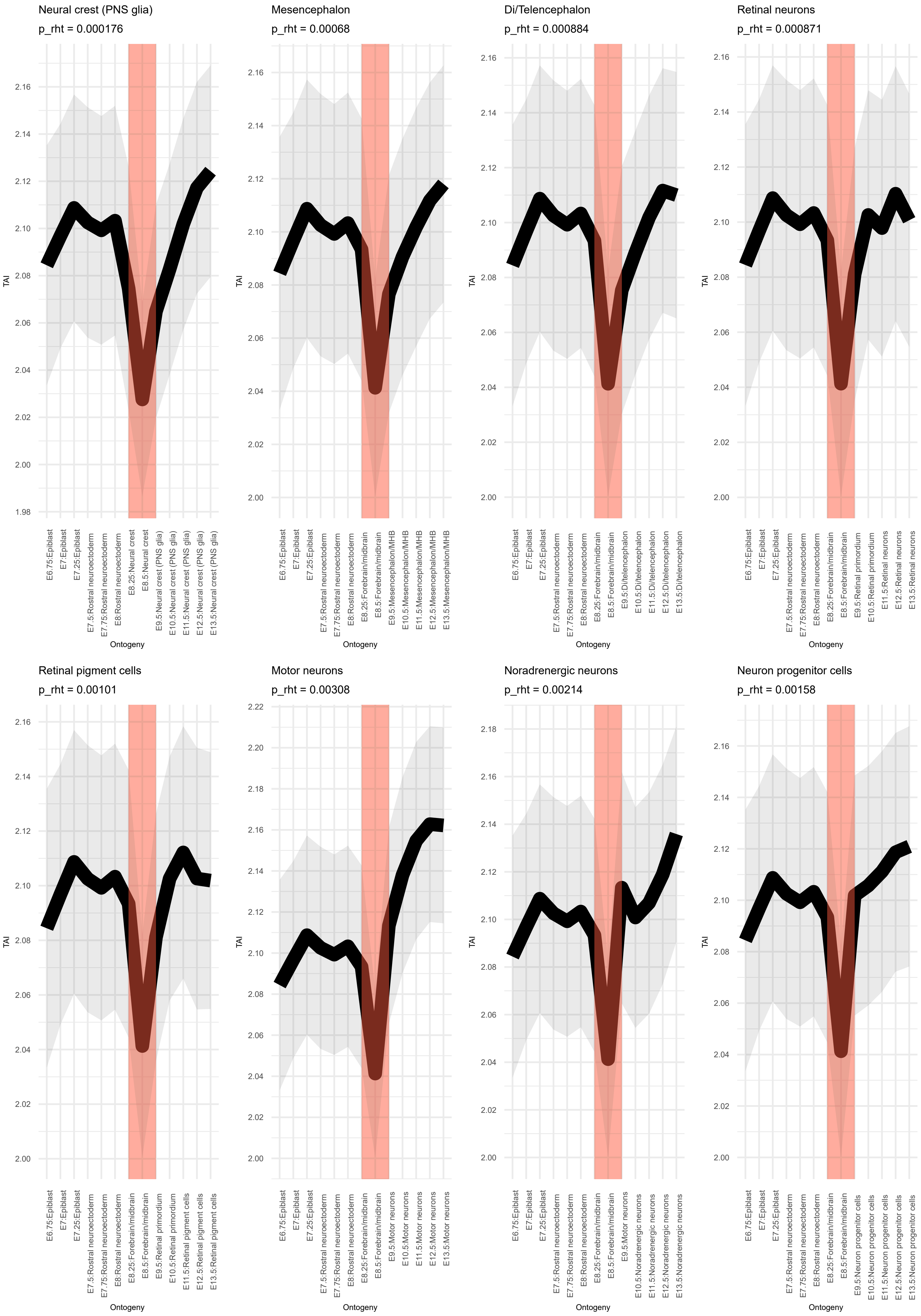
Neural crest (PNS glia)
p_rht = 0.000176
Mesencephalon
p_rht = 0.00068
Di/Telencephalon
p_rht = 0.000884
Retinal neurons
p_rht = 0.000871
Retinal pigment cells
p_rht = 0.00101
Motor neurons
p_rht = 0.00308
Noradrenergic neurons
p_rht = 0.00214
Neuron progenitor cells
p_rht = 0.00158
TAI
Ontogeny

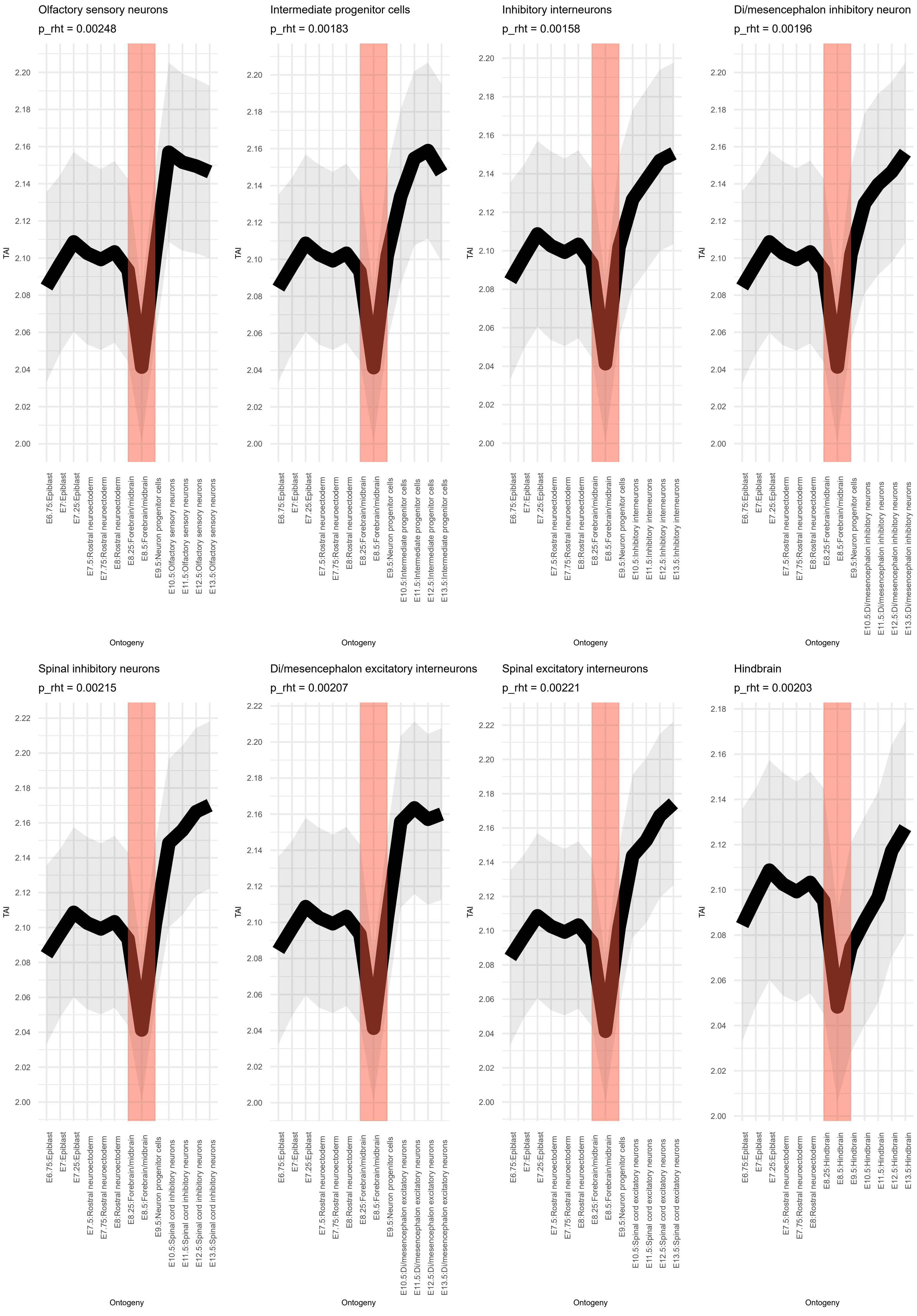
Olfactory sensory neurons
p_rht = 0.00248
Intermediate progenitor cells
p_rht = 0.00183
Inhibitory interneurons
p_rht = 0.00158
Di/mesencephalon inhibitory neuron
p_rht = 0.00196
Spinal inhibitory neurons
p_rht = 0.00215
Di/mesencephalon excitatory interneurons
p_rht = 0.00207
Spinal excitatory interneurons
p_rht = 0.00221
Hindbrain
p_rht = 0.00203
TAI
Ontogeny
E6.75:Epiblast
E7:Epiblast
E7.25:Epiblast
E7.5:Rostral neuroectoderm
E7.75:Rostral neuroectoderm
E8:Rostral neuroectoderm
E8.25:Forebrain/midbrain
E8.5:Forebrain/midbrain
E9.5:Neuron progenitor cells
E10.5:Olfactory sensory neurons
E11.5:Olfactory sensory neurons
E12.5:Olfactory sensory neurons
E13.5:Olfactory sensory neurons
E10.5:Intermediate progenitor cells
E11.5:Intermediate progenitor cells
E12.5:Intermediate progenitor cells
E13.5:Intermediate progenitor cells
E10.5:Inhibitory interneurons
E11.5:Inhibitory interneurons
E12.5:Inhibitory interneurons
E13.5:Inhibitory interneurons
E10.5:Di/mesencephalon inhibitory neurons
E11.5:Di/mesencephalon inhibitory neurons
E12.5:Di/mesencephalon inhibitory neurons
E13.5:Di/mesencephalon inhibitory neurons
E10.5:Spinal cord inhibitory neurons
E11.5:Spinal cord inhibitory neurons
E12.5:Spinal cord inhibitory neurons
E13.5:Spinal cord inhibitory neurons
E10.5:Di/mesencephalon excitatory neurons
E11.5:Di/mesencephalon excitatory neurons
E12.5:Di/mesencephalon excitatory neurons
E13.5:Di/mesencephalon excitatory neurons
E10.5:Spinal cord excitatory neurons
E11.5:Spinal cord excitatory neurons
E12.5:Spinal cord excitatory neurons
E13.5:Spinal cord excitatory neurons
E8.25:Hindbrain
E8.5:Hindbrain
E9.5:Hindbrain
E10.5:Hindbrain
E11.5:Hindbrain
E12.5:Hindbrain
E13.5:Hindbrain

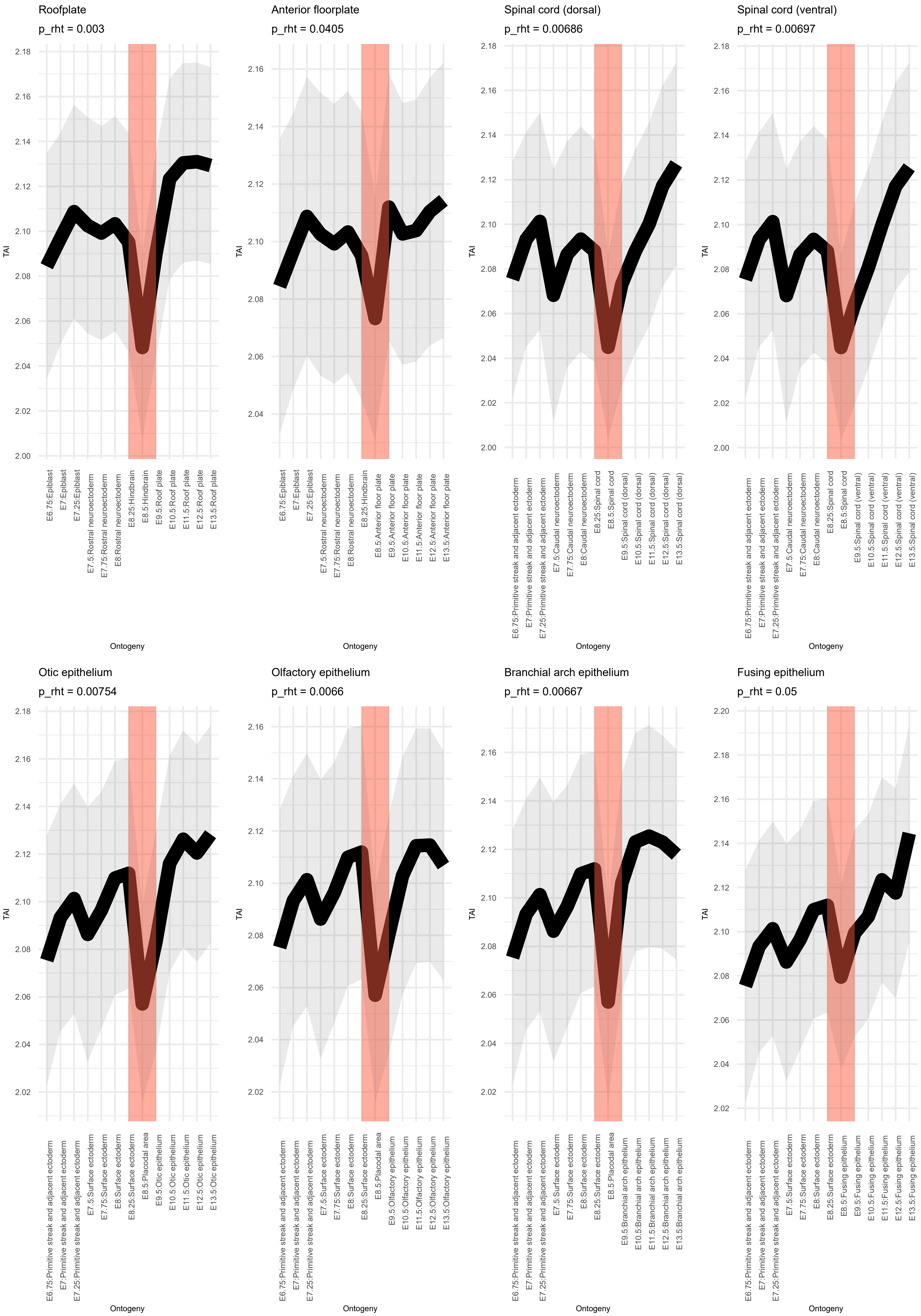

Roofplate
p_rht = 0.003
Anterior floorplate
p_rht = 0.0405
Spinal cord (dorsal)
p_rht = 0.00686
Spinal cord (ventral)
p_rht = 0.00697
Otic epithelium
p_rht = 0.00754
Olfactory epithelium
p_rht = 0.0066
Branchial arch epithelium
p_rht = 0.00667
Fusing epithelium
p_rht = 0.05
TAI
Ontogeny

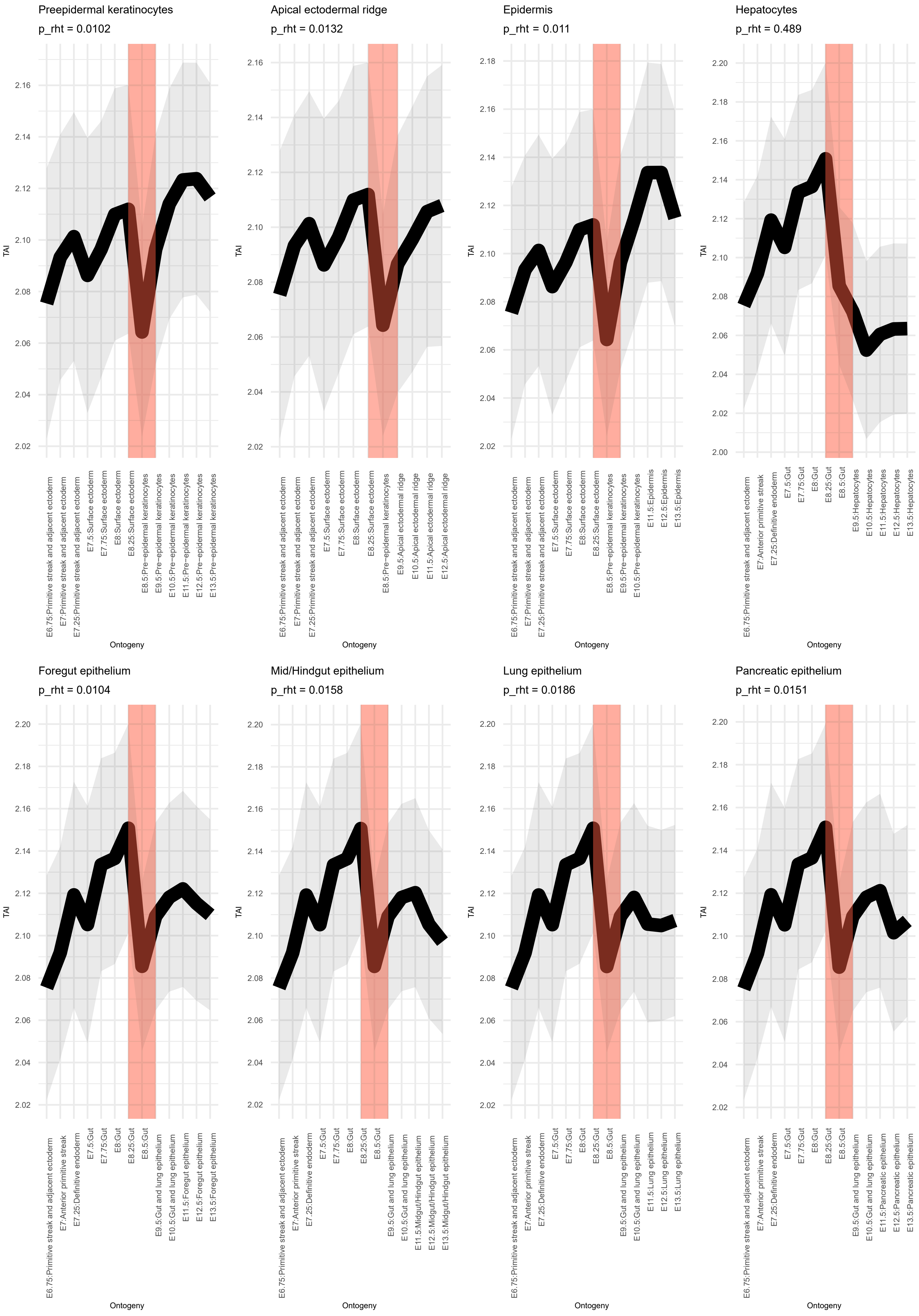
Preepidermal keratinocytes
p_rht = 0.0102
Apical ectodermal ridge
p_rht = 0.0132
Epidermis
p_rht = 0.011
Hepatocytes
p_rht = 0.489
Foregut epithelium
p_rht = 0.0104
Mid/Hindgut epithelium
p_rht = 0.0158
Lung epithelium
p_rht = 0.0186
Pancreatic epithelium
p_rht = 0.0151
TAI
Ontogeny
E6.75:Primitive streak and adjacent ectoderm
E7:Primitive streak and adjacent ectoderm
E7.25:Primitive streak and adjacent ectoderm
E7.5:Surface ectoderm
E7.75:Surface ectoderm
E8:Surface ectoderm
E8.25:Surface ectoderm
E8.5:Pre−epidermal keratinocytes
E9.5:Pre−epidermal keratinocytes
E10.5:Pre−epidermal keratinocytes
E11.5:Pre−epidermal keratinocytes
E12.5:Pre−epidermal keratinocytes
E13.5:Pre−epidermal keratinocytes
E9.5:Apical ectodermal ridge
E10.5:Apical ectodermal ridge
E11.5:Apical ectodermal ridge
E12.5:Apical ectodermal ridge
E11.5:Epidermis
E12.5:Epidermis
E13.5:Epidermis
E7:Anterior primitive streak
E7.25:Definitive endoderm
E7.5:Gut
E7.75:Gut
E8:Gut
E8.25:Gut
E8.5:Gut
E9.5:Hepatocytes
E10.5:Hepatocytes
E11.5:Hepatocytes
E12.5:Hepatocytes
E13.5:Hepatocytes
E9.5:Gut and lung epithelium
E10.5:Gut and lung epithelium
E11.5:Foregut epithelium
E12.5:Foregut epithelium
E13.5:Foregut epithelium
E11.5:Midgut/Hindgut epithelium
E12.5:Midgut/Hindgut epithelium
E13.5:Midgut/Hindgut epithelium
E11.5:Lung epithelium
E12.5:Lung epithelium
E13.5:Lung epithelium
E11.5:Pancreatic epithelium
E12.5:Pancreatic epithelium
E13.5:Pancreatic epithelium

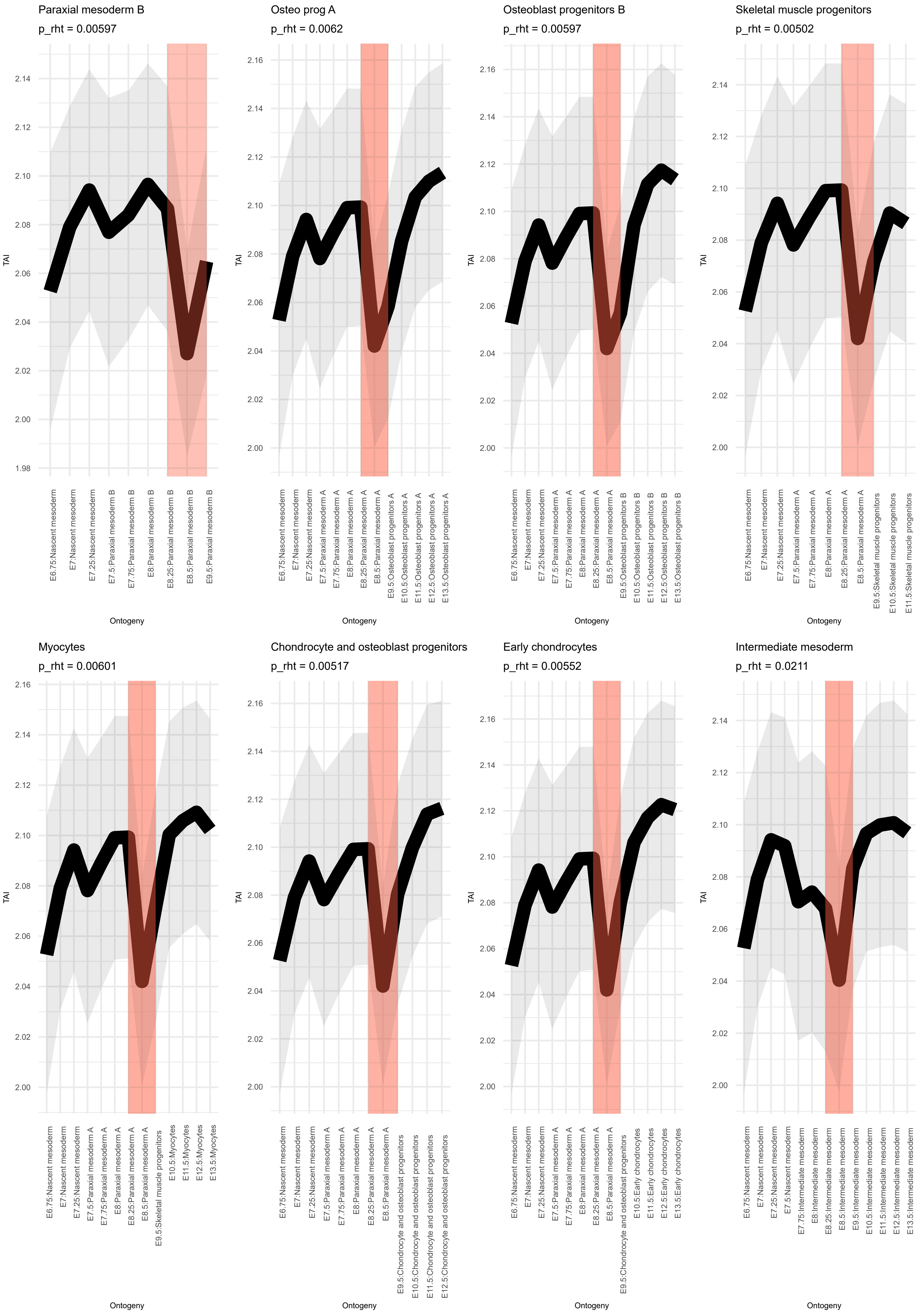

Paraxial mesoderm B
p_rht = 0.00597
Osteo prog A
p_rht = 0.0062
Osteoblast progenitors B
p_rht = 0.00597
Skeletal muscle progenitors
p_rht = 0.00502
Myocytes
p_rht = 0.00601
Chondrocyte and osteoblast progenitors
p_rht = 0.00517
Early chondrocytes
p_rht = 0.00552
Intermediate mesoderm
p_rht = 0.0211
TAI
Ontogeny

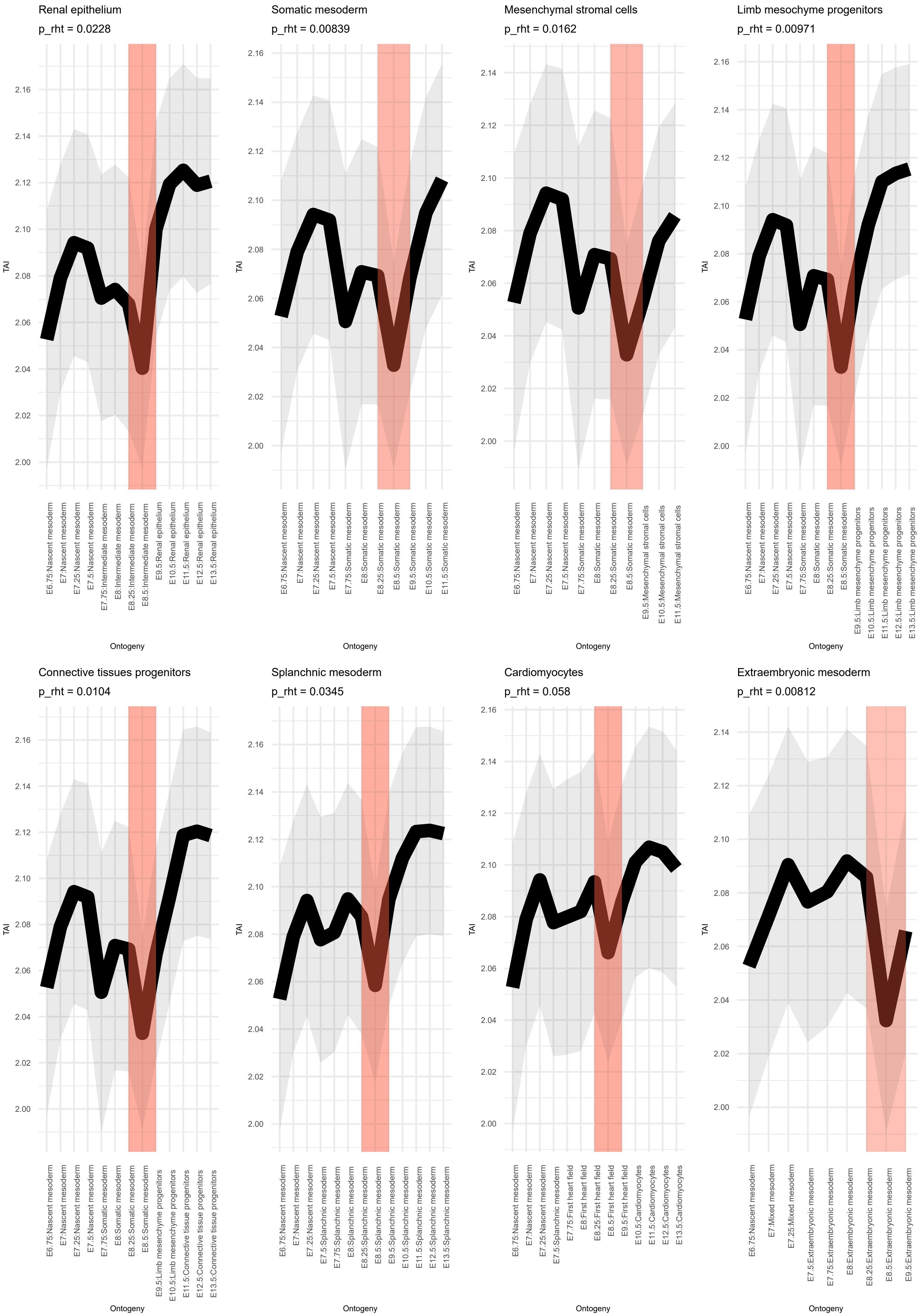
Renal epithelium
p_rht = 0.0228
TAI
Ontogeny
E6.75:Nascent mesoderm
E7:Nascent mesoderm
E7.25:Nascent mesoderm
E7.5:Nascent mesoderm
E7.75:Intermediate mesoderm
E8:Intermediate mesoderm
E8.25:Intermediate mesoderm
E8.5:Intermediate mesoderm
E9.5:Renal epithelium
E10.5:Renal epithelium
E11.5:Renal epithelium
E12.5:Renal epithelium
E13.5:Renal epithelium
Somatic mesoderm
p_rht = 0.00839
TAI
Ontogeny
E6.75:Nascent mesoderm
E7:Nascent mesoderm
E7.25:Nascent mesoderm
E7.5:Nascent mesoderm
E7.75:Somatic mesoderm
E8:Somatic mesoderm
E8.25:Somatic mesoderm
E8.5:Somatic mesoderm
E9.5:Somatic mesoderm
E10.5:Somatic mesoderm
E11.5:Somatic mesoderm
Mesenchymal stromal cells
p_rht = 0.0162
TAI
Ontogeny
E6.75:Nascent mesoderm
E7:Nascent mesoderm
E7.25:Nascent mesoderm
E7.5:Nascent mesoderm
E7.75:Somatic mesoderm
E8:Somatic mesoderm
E8.25:Somatic mesoderm
E8.5:Somatic mesoderm
E9.5:Mesenchymal stromal cells
E10.5:Mesenchymal stromal cells
E11.5:Mesenchymal stromal cells
Limb mesochyme progenitors
p_rht = 0.00971
TAI
Ontogeny
E6.75:Nascent mesoderm
E7:Nascent mesoderm
E7.25:Nascent mesoderm
E7.5:Nascent mesoderm
E7.75:Somatic mesoderm
E8:Somatic mesoderm
E8.25:Somatic mesoderm
E8.5:Somatic mesoderm
E9.5:Limb mesenchyme progenitors
E10.5:Limb mesenchyme progenitors
E11.5:Limb mesenchyme progenitors
E12.5:Limb mesenchyme progenitors
E13.5:Limb mesenchyme progenitors
Connective tissues progenitors
p_rht = 0.0104
TAI
Ontogeny
E6.75:Nascent mesoderm
E7:Nascent mesoderm
E7.25:Nascent mesoderm
E7.5:Nascent mesoderm
E7.75:Somatic mesoderm
E8:Somatic mesoderm
E8.25:Somatic mesoderm
E8.5:Somatic mesoderm
E9.5:Limb mesenchyme progenitors
E10.5:Limb mesenchyme progenitors
E11.5:Connective tissue progenitors
E12.5:Connective tissue progenitors
E13.5:Connective tissue progenitors
Splanchnic mesoderm
p_rht = 0.0345
TAI
Ontogeny
E6.75:Nascent mesoderm
E7:Nascent mesoderm
E7.25:Nascent mesoderm
E7.5:Splanchnic mesoderm
E7.75:Splanchnic mesoderm
E8:Splanchnic mesoderm
E8.25:Splanchnic mesoderm
E8.5:Splanchnic mesoderm
E9.5:Splanchnic mesoderm
E10.5:Splanchnic mesoderm
E11.5:Splanchnic mesoderm
E12.5:Splanchnic mesoderm
E13.5:Splanchnic mesoderm
Cardiomyocytes
p_rht = 0.058
TAI
Ontogeny
E6.75:Nascent mesoderm
E7:Nascent mesoderm
E7.25:Nascent mesoderm
E7.5:Splanchnic mesoderm
E7.75:First heart field
E8:First heart field
E8.25:First heart field
E8.5:First heart field
E9.5:First heart field
E10.5:Cardiomyocytes
E11.5:Cardiomyocytes
E12.5:Cardiomyocytes
E13.5:Cardiomyocytes
Extraembryonic mesoderm
p_rht = 0.00812
TAI
Ontogeny
E6.75:Nascent mesoderm
E7:Mixed mesoderm
E7.25:Mixed mesoderm
E7.5:Extraembryonic mesoderm
E7.75:Extraembryonic mesoderm
E8:Extraembryonic mesoderm
E8.25:Extraembryonic mesoderm
E8.5:Extraembryonic mesoderm
E9.5:Extraembryonic mesoderm

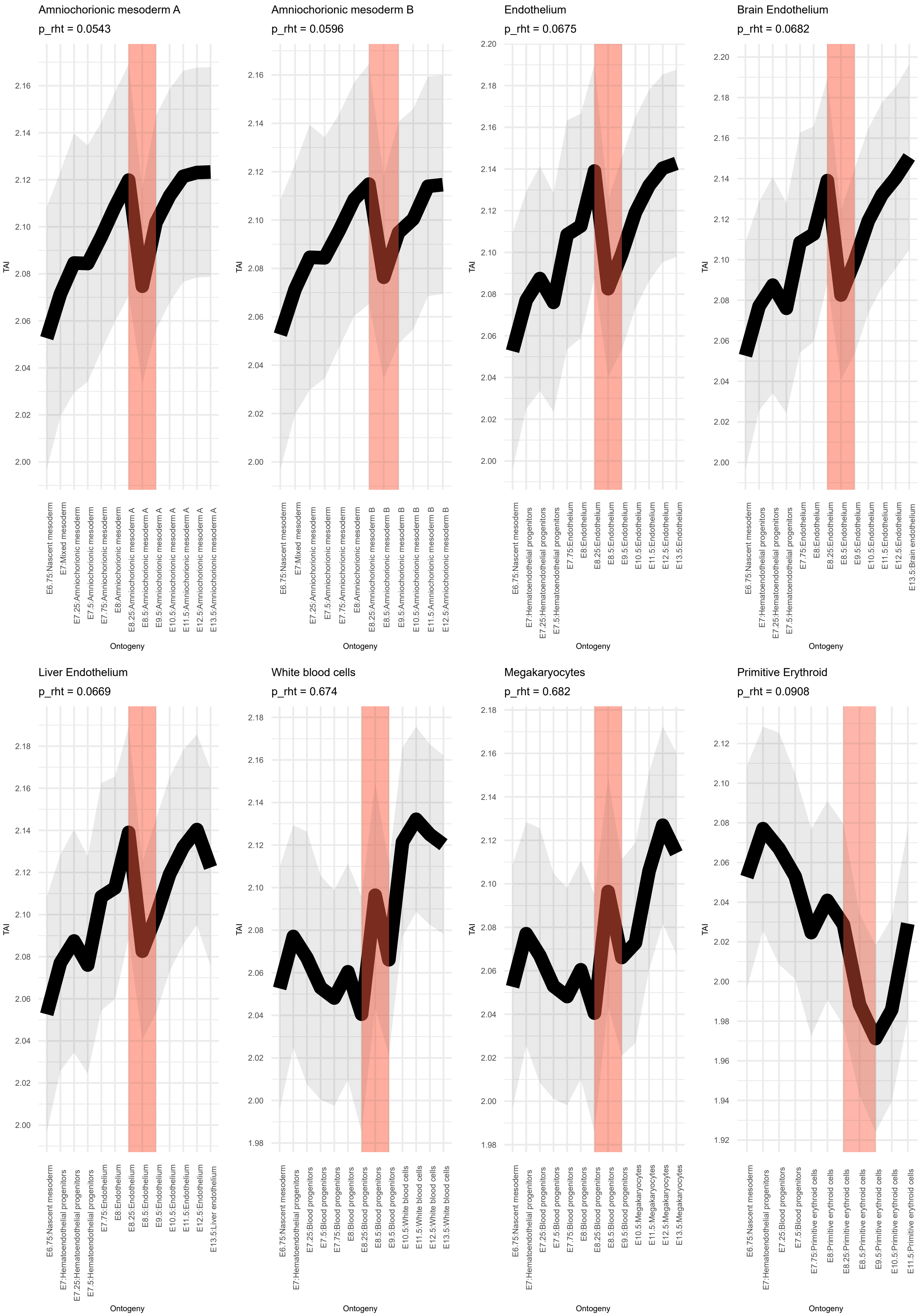
Amniochorionic mesoderm A
p_rht = 0.0543
Amniochorionic mesoderm B
p_rht = 0.0596
Endothelium
p_rht = 0.0675
Brain Endothelium
p_rht = 0.0682
Liver Endothelium
p_rht = 0.0669
White blood cells
p_rht = 0.674
Megakaryocytes
p_rht = 0.682
Primitive Erythroid
p_rht = 0.0908
TAI
Ontogeny
E6.75:Nascent mesoderm
E7:Mixed mesoderm
E7.25:Amniochorionic mesoderm
E7.5:Amniochorionic mesoderm
E7.75:Amniochorionic mesoderm
E8:Amniochorionic mesoderm
E8.25:Amniochorionic mesoderm A
E8.5:Amniochorionic mesoderm A
E9.5:Amniochorionic mesoderm A
E10.5:Amniochorionic mesoderm A
E11.5:Amniochorionic mesoderm A
E12.5:Amniochorionic mesoderm A
E13.5:Amniochorionic mesoderm A
E8.25:Amniochorionic mesoderm B
E8.5:Amniochorionic mesoderm B
E9.5:Amniochorionic mesoderm B
E10.5:Amniochorionic mesoderm B
E11.5:Amniochorionic mesoderm B
E12.5:Amniochorionic mesoderm B
E7:Hematoendothelial progenitors
E7.25:Hematoendothelial progenitors
E7.5:Hematoendothelial progenitors
E7.75:Endothelium
E8:Endothelium
E8.25:Endothelium
E8.5:Endothelium
E9.5:Endothelium
E10.5:Endothelium
E11.5:Endothelium
E12.5:Endothelium
E13.5:Endothelium
E13.5:Brain endothelium
E13.5:Liver endothelium
E7.25:Blood progenitors
E7.5:Blood progenitors
E7.75:Blood progenitors
E8:Blood progenitors
E8.25:Blood progenitors
E8.5:Blood progenitors
E9.5:Blood progenitors
E10.5:White blood cells
E11.5:White blood cells
E12.5:White blood cells
E13.5:White blood cells
E10.5:Megakaryocytes
E11.5:Megakaryocytes
E12.5:Megakaryocytes
E13.5:Megakaryocytes
E7.75:Primitive erythroid cells
E8:Primitive erythroid cells
E8.25:Primitive erythroid cells
E8.5:Primitive erythroid cells
E9.5:Primitive erythroid cells
E10.5:Primitive erythroid cells
E11.5:Primitive erythroid cells

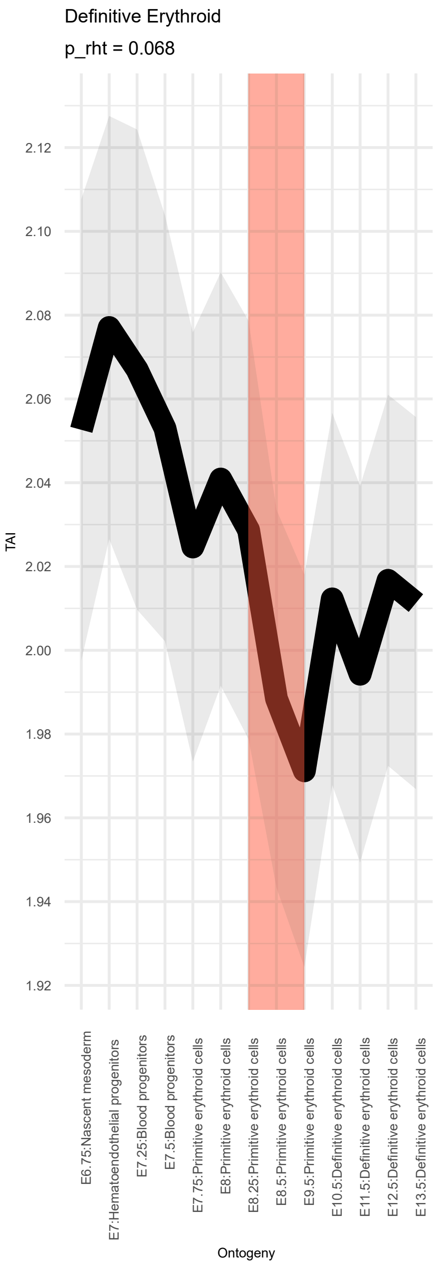

Supp. Fig. 4. Reductive hourglass tests for the TAI profile of individual mouse cellular trajectories. Significance was assessed using 10,000 permutations, with a p-value ≤ 0.05 considered significant. The hourglass pattern was tested by defining early stages as E6.75–E8.25, mid as E8.5, and late as E9.5–E13.5.

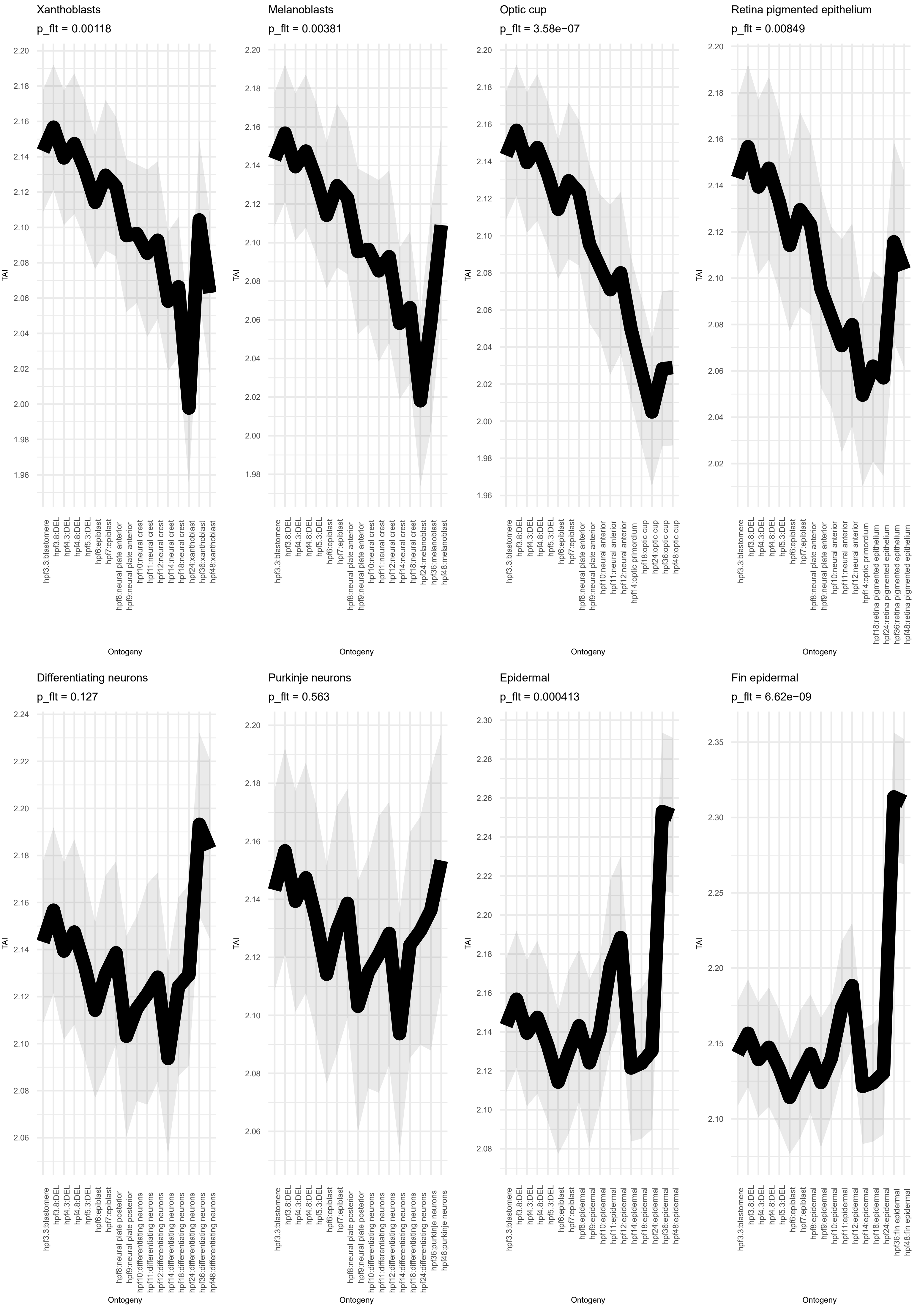

Xanthoblasts
p_flt = 0.00118
TAI
Ontogeny
hpf3.3:blastomere
hpf3.8:DEL
hpf4.3:DEL
hpf4.8:DEL
hpf5.3:DEL
hpf6:epiblast
hpf7:epiblast
hpf8:neural plate anterior
hpf9:neural plate anterior
hpf10:neural crest
hpf11:neural crest
hpf12:neural crest
hpf14:neural crest
hpf18:neural crest
hpf24:xanthoblast
hpf36:xanthoblast
hpf48:xanthoblast
Melanoblasts
p_flt = 0.00381
TAI
Ontogeny
hpf24:melanoblast
hpf36:melanoblast
hpf48:melanoblast
Optic cup
p_flt = 3.58e−07
TAI
Ontogeny
hpf10:neural anterior
hpf11:neural anterior
hpf12:neural anterior
hpf14:optic primordium
hpf18:optic cup
hpf24:optic cup
hpf36:optic cup
hpf48:optic cup
Retina pigmented epithelium
p_flt = 0.00849
TAI
Ontogeny
hpf18:retina pigmented epithelium
hpf24:retina pigmented epithelium
hpf36:retina pigmented epithelium
hpf48:retina pigmented epithelium
Differentiating neurons
p_flt = 0.127
TAI
Ontogeny
hpf8:neural plate posterior
hpf9:neural plate posterior
hpf10:differentiating neurons
hpf11:differentiating neurons
hpf12:differentiating neurons
hpf14:differentiating neurons
hpf18:differentiating neurons
hpf24:differentiating neurons
hpf36:differentiating neurons
hpf48:differentiating neurons
Purkinje neurons
p_flt = 0.563
TAI
Ontogeny
hpf36:purkinje neurons
hpf48:purkinje neurons
Epidermal
p_flt = 0.000413
TAI
Ontogeny
hpf8:epidermal
hpf9:epidermal
hpf10:epidermal
hpf11:epidermal
hpf12:epidermal
hpf14:epidermal
hpf18:epidermal
hpf24:epidermal
hpf36:epidermal
hpf48:epidermal
Fin epidermal
p_flt = 6.62e−09
TAI
Ontogeny
hpf36:fin epidermal
hpf48:fin epidermal

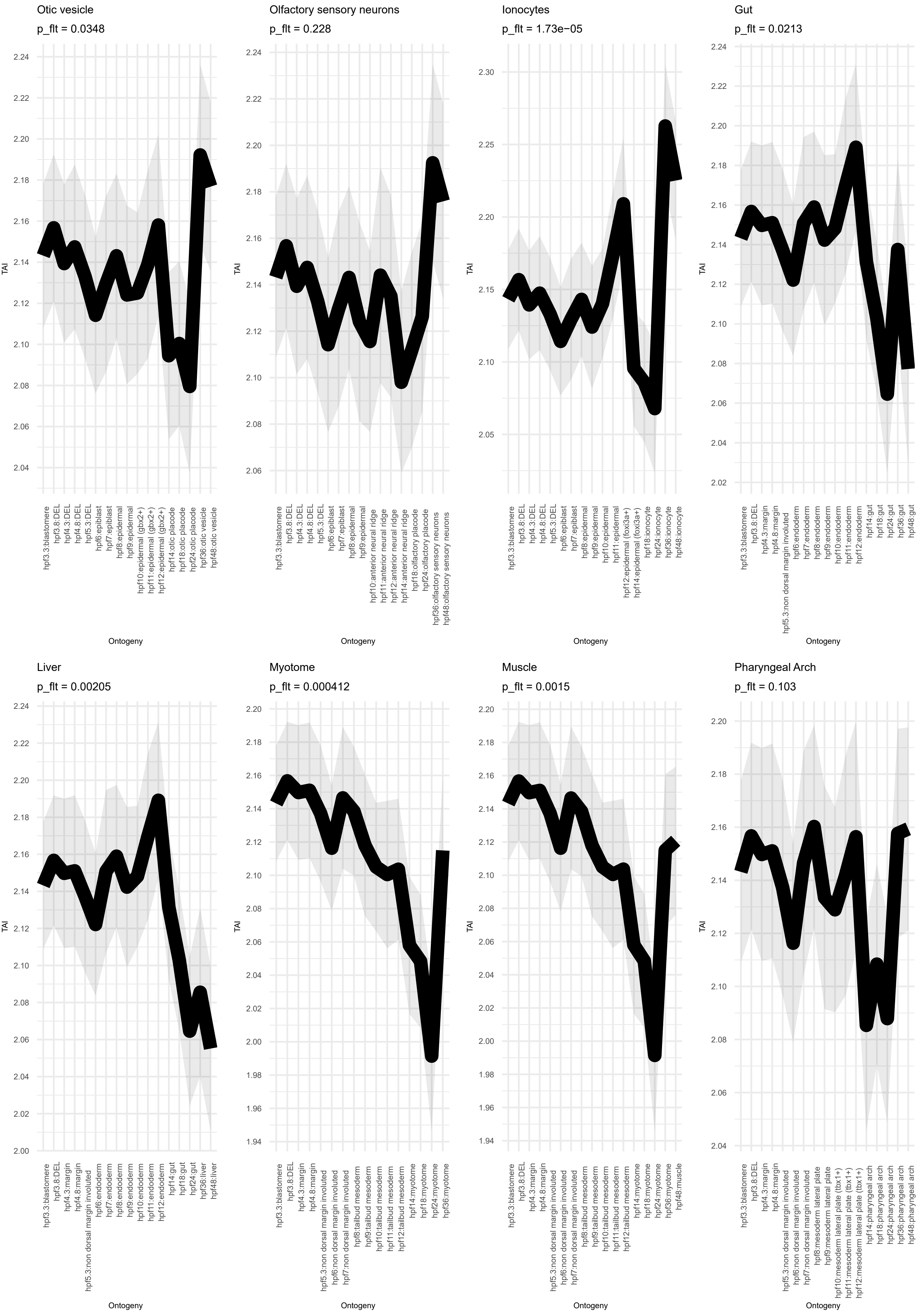

Otic vesicle
p_flt = 0.0348
TAI
hpf3.3:blastomere
hpf3.8:DEL
hpf4.3:DEL
hpf4.8:DEL
hpf5.3:DEL
hpf6:epiblast
hpf7:epiblast
hpf8:epidermal
hpf9:epidermal
hpf10:epidermal (gbx2+)
hpf11:epidermal (gbx2+)
hpf12:epidermal (gbx2+)
hpf14:otic placode
hpf18:otic placode
hpf24:otic placode
hpf36:otic vesicle
hpf48:otic vesicle
Ontogeny
Olfactory sensory neurons
p_flt = 0.228
TAI
hpf3.3:blastomere
hpf3.8:DEL
hpf4.3:DEL
hpf4.8:DEL
hpf5.3:DEL
hpf6:epiblast
hpf7:epiblast
hpf8:epidermal
hpf9:epidermal
hpf10:anterior neural ridge
hpf11:anterior neural ridge
hpf12:anterior neural ridge
hpf14:anterior neural ridge
hpf18:olfactory placode
hpf24:olfactory placode
hpf36:olfactory sensory neurons
hpf48:olfactory sensory neurons
Ontogeny
Ionocytes
p_flt = 1.73e−05
TAI
hpf3.3:blastomere
hpf3.8:DEL
hpf4.3:DEL
hpf4.8:DEL
hpf5.3:DEL
hpf6:epiblast
hpf7:epiblast
hpf8:epidermal
hpf9:epidermal
hpf10:epidermal
hpf11:epidermal
hpf12:epidermal (foxi3a+)
hpf14:epidermal (foxi3a+)
hpf18:ionocyte
hpf24:ionocyte
hpf36:ionocyte
hpf48:ionocyte
Ontogeny
Gut
p_flt = 0.0213
TAI
hpf3.3:blastomere
hpf3.8:DEL
hpf4.3:margin
hpf4.8:margin
hpf5.3:non dorsal margin involuted
hpf6:endoderm
hpf7:endoderm
hpf8:endoderm
hpf9:endoderm
hpf10:endoderm
hpf11:endoderm
hpf12:endoderm
hpf14:gut
hpf18:gut
hpf24:gut
hpf36:gut
hpf48:gut
Ontogeny
Liver
p_flt = 0.00205
TAI
hpf3.3:blastomere
hpf3.8:DEL
hpf4.3:margin
hpf4.8:margin
hpf5.3:non dorsal margin involuted
hpf6:endoderm
hpf7:endoderm
hpf8:endoderm
hpf9:endoderm
hpf10:endoderm
hpf11:endoderm
hpf12:endoderm
hpf14:gut
hpf18:gut
hpf24:gut
hpf36:liver
hpf48:liver
Ontogeny
Myotome
p_flt = 0.000412
TAI
hpf3.3:blastomere
hpf3.8:DEL
hpf4.3:margin
hpf4.8:margin
hpf5.3:non dorsal margin involuted
hpf6:non dorsal margin involuted
hpf7:non dorsal margin involuted
hpf8:tailbud mesoderm
hpf9:tailbud mesoderm
hpf10:tailbud mesoderm
hpf11:tailbud mesoderm
hpf12:tailbud mesoderm
hpf14:myotome
hpf18:myotome
hpf24:myotome
hpf36:myotome
Ontogeny
Muscle
p_flt = 0.0015
TAI
hpf3.3:blastomere
hpf3.8:DEL
hpf4.3:margin
hpf4.8:margin
hpf5.3:non dorsal margin involuted
hpf6:non dorsal margin involuted
hpf7:non dorsal margin involuted
hpf8:tailbud mesoderm
hpf9:tailbud mesoderm
hpf10:tailbud mesoderm
hpf11:tailbud mesoderm
hpf12:tailbud mesoderm
hpf14:myotome
hpf18:myotome
hpf24:myotome
hpf36:myotome
hpf48:muscle
Ontogeny
Pharyngeal Arch
p_flt = 0.103
TAI
hpf3.3:blastomere
hpf3.8:DEL
hpf4.3:margin
hpf4.8:margin
hpf5.3:non dorsal margin involuted
hpf6:non dorsal margin involuted
hpf7:non dorsal margin involuted
hpf8:mesoderm lateral plate
hpf9:mesoderm lateral plate
hpf10:mesoderm lateral plate (tbx1+)
hpf11:mesoderm lateral plate (tbx1+)
hpf12:mesoderm lateral plate (tbx1+)
hpf14:pharyngeal arch
hpf18:pharyngeal arch
hpf24:pharyngeal arch
hpf36:pharyngeal arch
hpf48:pharyngeal arch
Ontogeny

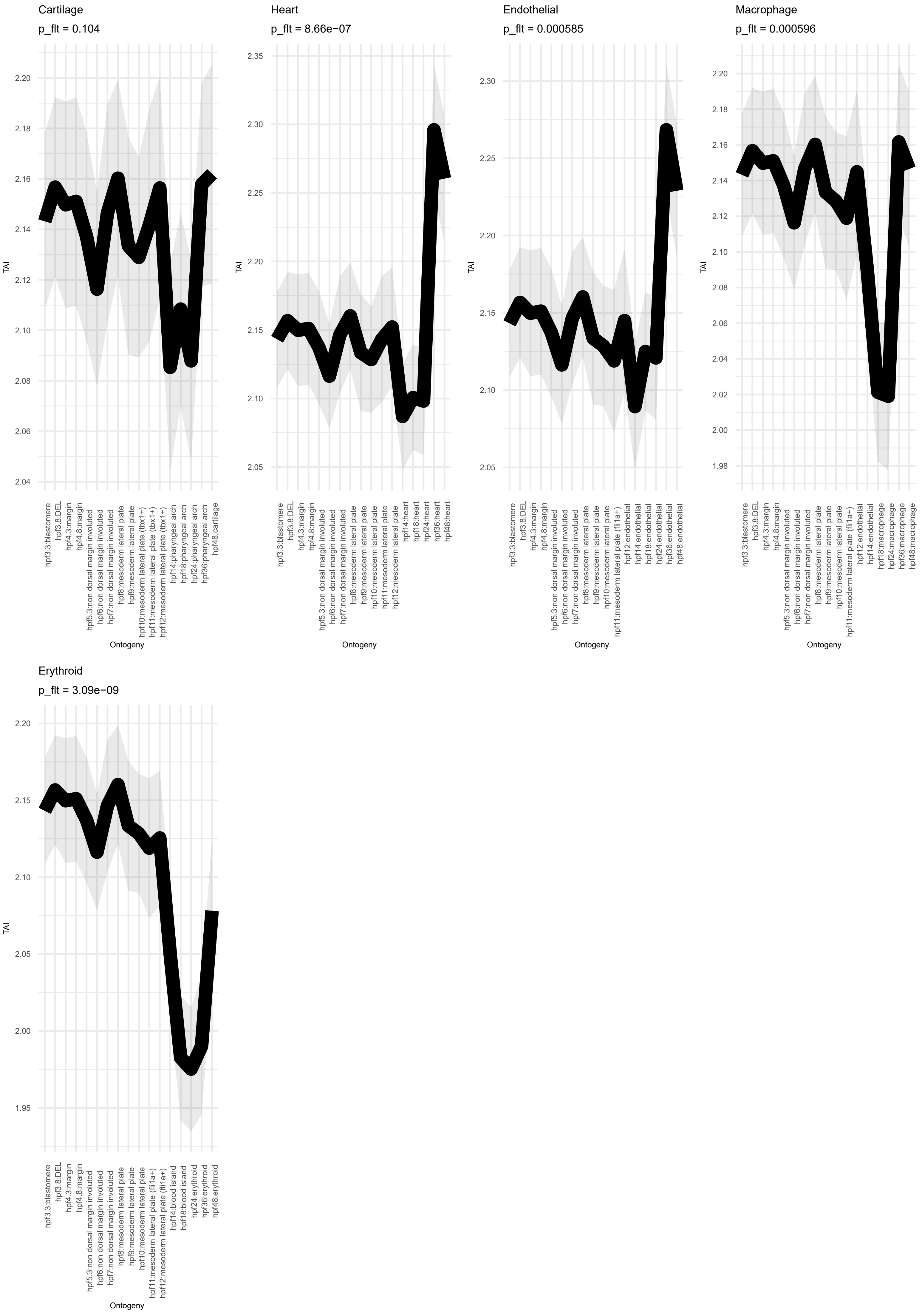

Cartilage
p_flt = 0.104
TAI
2.04
2.06
2.08
2.10
2.12
2.14
2.16
2.18
2.20
hpf3.3:blastomere
hpf3.8:DEL
hpf4.3:margin
hpf4.8:margin
hpf5.3:non dorsal margin involuted
hpf6:non dorsal margin involuted
hpf7:non dorsal margin involuted
hpf8:mesoderm lateral plate
hpf9:mesoderm lateral plate
hpf10:mesoderm lateral plate (tbx1+)
hpf11:mesoderm lateral plate (tbx1+)
hpf12:mesoderm lateral plate (tbx1+)
hpf14:pharyngeal arch
hpf18:pharyngeal arch
hpf24:pharyngeal arch
hpf36:pharyngeal arch
hpf48:cartilage
Ontogeny
Heart
p_flt = 8.66e−07
TAI
2.05
2.10
2.15
2.20
2.25
2.30
2.35
hpf3.3:blastomere
hpf3.8:DEL
hpf4.3:margin
hpf4.8:margin
hpf5.3:non dorsal margin involuted
hpf6:non dorsal margin involuted
hpf7:non dorsal margin involuted
hpf8:mesoderm lateral plate
hpf9:mesoderm lateral plate
hpf10:mesoderm lateral plate
hpf11:mesoderm lateral plate
hpf12:mesoderm lateral plate
hpf14:heart
hpf18:heart
hpf24:heart
hpf36:heart
hpf48:heart
Ontogeny
Endothelial
p_flt = 0.000585
TAI
2.05
2.10
2.15
2.20
2.25
2.30
hpf3.3:blastomere
hpf3.8:DEL
hpf4.3:margin
hpf4.8:margin
hpf5.3:non dorsal margin involuted
hpf6:non dorsal margin involuted
hpf7:non dorsal margin involuted
hpf8:mesoderm lateral plate
hpf9:mesoderm lateral plate
hpf10:mesoderm lateral plate
hpf11:mesoderm lateral plate (fli1a+)
hpf12:endothelial
hpf14:endothelial
hpf18:endothelial
hpf24:endothelial
hpf36:endothelial
hpf48:endothelial
Ontogeny
Macrophage
p_flt = 0.000596
TAI
1.98
2.00
2.02
2.04
2.06
2.08
2.10
2.12
2.14
2.16
2.18
2.20
hpf3.3:blastomere
hpf3.8:DEL
hpf4.3:margin
hpf4.8:margin
hpf5.3:non dorsal margin involuted
hpf6:non dorsal margin involuted
hpf7:non dorsal margin involuted
hpf8:mesoderm lateral plate
hpf9:mesoderm lateral plate
hpf10:mesoderm lateral plate
hpf11:mesoderm lateral plate (fli1a+)
hpf12:endothelial
hpf14:endothelial
hpf18:macrophage
hpf24:macrophage
hpf36:macrophage
hpf48:macrophage
Ontogeny
Erythroid
p_flt = 3.09e−09
TAI
1.95
2.00
2.05
2.10
2.15
2.20
hpf3.3:blastomere
hpf3.8:DEL
hpf4.3:margin
hpf4.8:margin
hpf5.3:non dorsal margin involuted
hpf6:non dorsal margin involuted
hpf7:non dorsal margin involuted
hpf8:mesoderm lateral plate
hpf9:mesoderm lateral plate
hpf10:mesoderm lateral plate
hpf11:mesoderm lateral plate (fli1a+)
hpf12:mesoderm lateral plate (fli1a+)
hpf14:blood island
hpf18:blood island
hpf24:erythroid
hpf36:erythroid
hpf48:erythroid
Ontogeny

Supp. Fig. 5. Flatline tests for the TAI profile of individual zebrafish cellular trajectories. Significance was assessed using 10,000 permutations, with a p-value ≤ 0.05 considered significant.

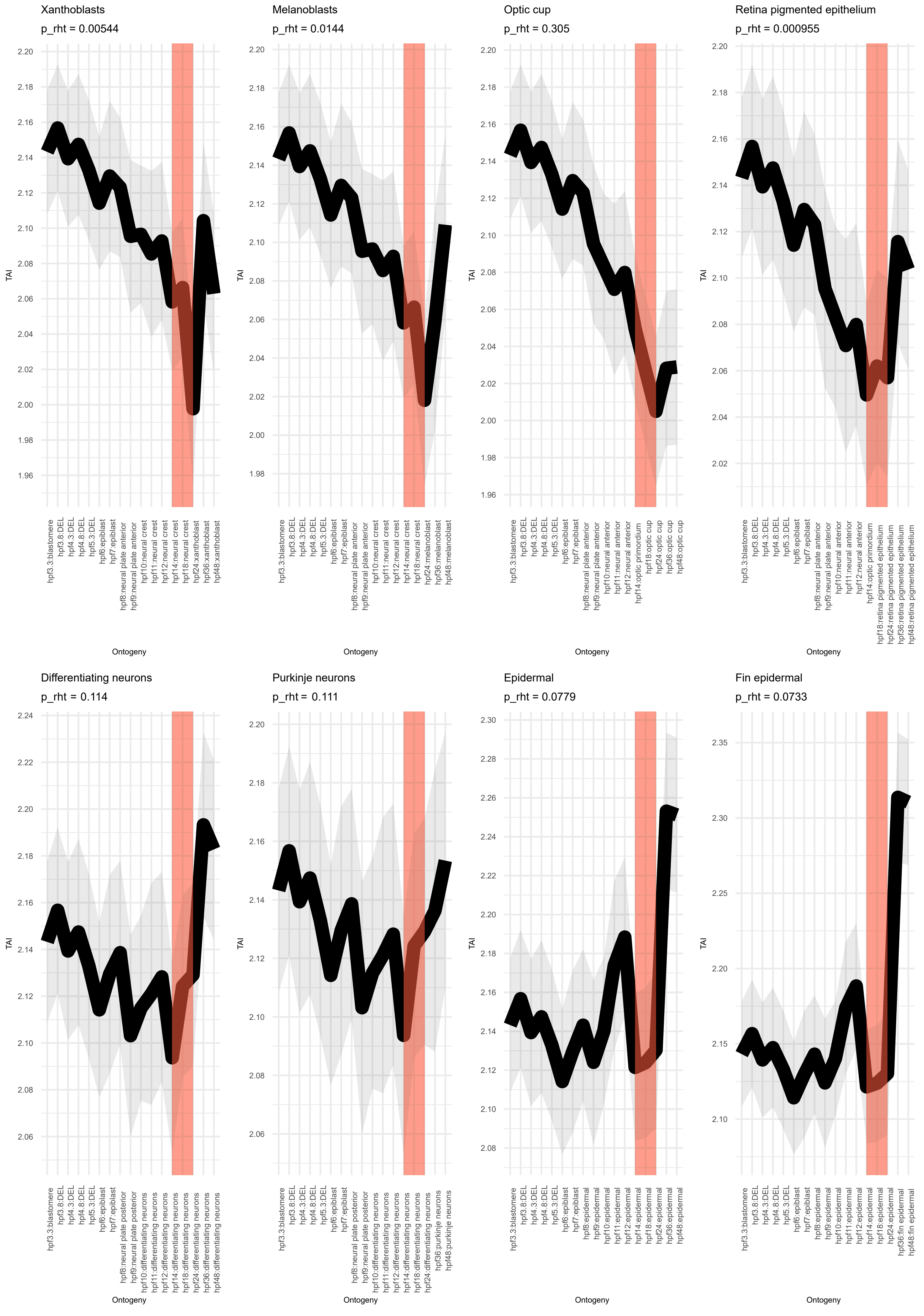
Xanthoblasts
p_rht = 0.00544
Melanoblasts
p_rht = 0.0144
Optic cup
p_rht = 0.305
Retina pigmented epithelium
p_rht = 0.000955
Differentiating neurons
p_rht = 0.114
Purkinje neurons
p_rht = 0.111
Epidermal
p_rht = 0.0779
Fin epidermal
p_rht = 0.0733
TAI
Ontogeny

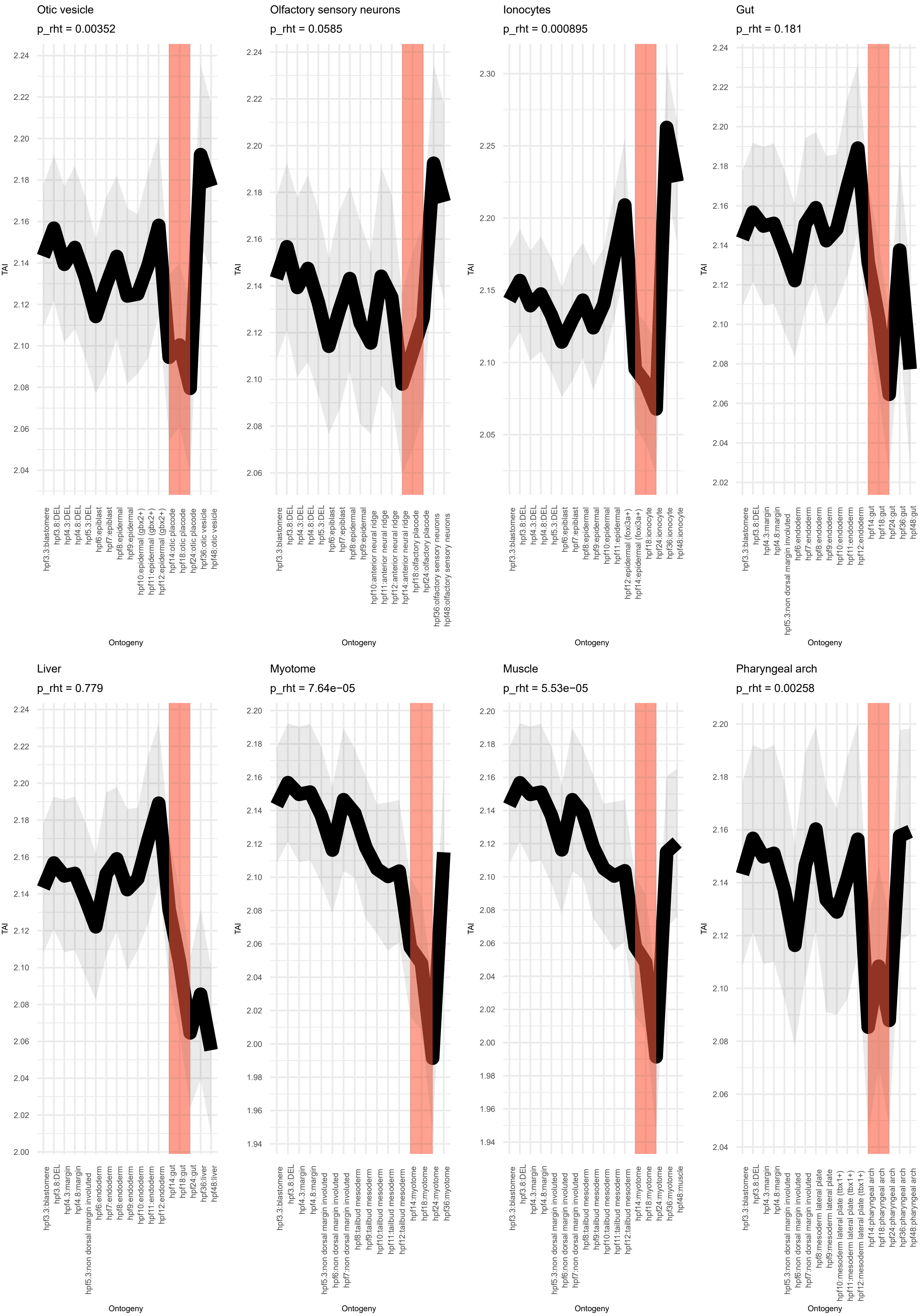

Otic vesicle
p_rht = 0.00352
Olfactory sensory neurons
p_rht = 0.0585
Ionocytes
p_rht = 0.000895
Gut
p_rht = 0.181
Liver
p_rht = 0.779
Myotome
p_rht = 7.64e−05
Muscle
p_rht = 5.53e−05
Pharyngeal arch
p_rht = 0.00258
TAI
Ontogeny

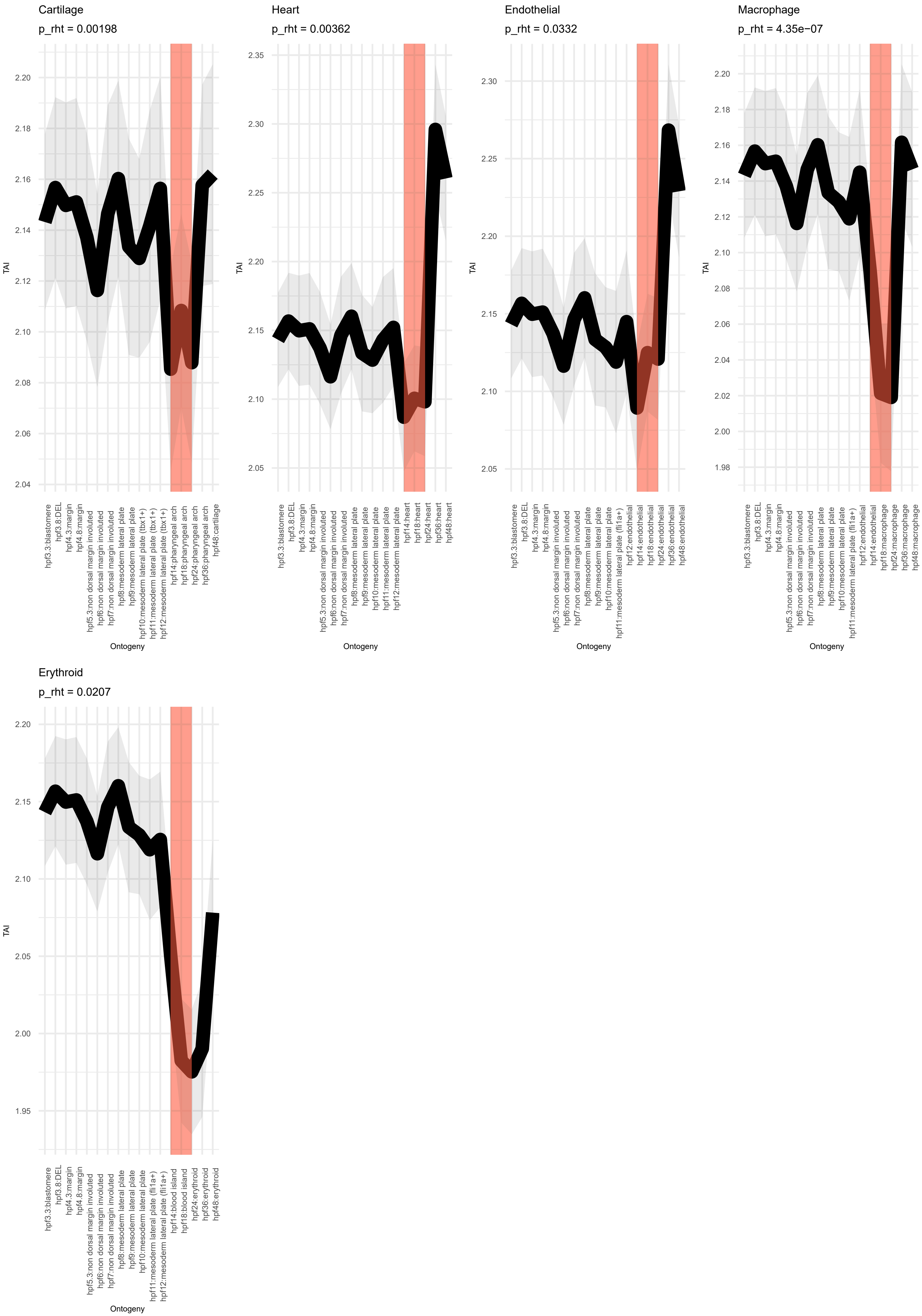
Cartilage
p_rht = 0.00198
TAI
2.20
2.18
2.16
2.14
2.12
2.10
2.08
2.06
2.04
hpf3.3:blastomere
hpf3.8:DEL
hpf4.3:margin
hpf4.8:margin
hpf5.3:non dorsal margin involuted
hpf6:non dorsal margin involuted
hpf7:non dorsal margin involuted
hpf8:mesoderm lateral plate
hpf9:mesoderm lateral plate
hpf10:mesoderm lateral plate (tbx1+)
hpf11:mesoderm lateral plate (tbx1+)
hpf12:mesoderm lateral plate (tbx1+)
hpf14:pharyngeal arch
hpf18:pharyngeal arch
hpf24:pharyngeal arch
hpf36:pharyngeal arch
hpf48:cartilage
Ontogeny
Heart
p_rht = 0.00362
TAI
2.35
2.30
2.25
2.20
2.15
2.10
2.05
hpf3.3:blastomere
hpf3.8:DEL
hpf4.3:margin
hpf4.8:margin
hpf5.3:non dorsal margin involuted
hpf6:non dorsal margin involuted
hpf7:non dorsal margin involuted
hpf8:mesoderm lateral plate
hpf9:mesoderm lateral plate
hpf10:mesoderm lateral plate
hpf11:mesoderm lateral plate
hpf12:mesoderm lateral plate
hpf14:heart
hpf18:heart
hpf24:heart
hpf36:heart
hpf48:heart
Ontogeny
Endothelial
p_rht = 0.0332
TAI
2.30
2.25
2.20
2.15
2.10
2.05
hpf3.3:blastomere
hpf3.8:DEL
hpf4.3:margin
hpf4.8:margin
hpf5.3:non dorsal margin involuted
hpf6:non dorsal margin involuted
hpf7:non dorsal margin involuted
hpf8:mesoderm lateral plate
hpf9:mesoderm lateral plate
hpf10:mesoderm lateral plate
hpf11:mesoderm lateral plate (fli1a+)
hpf12:endothelial
hpf14:endothelial
hpf18:endothelial
hpf24:endothelial
hpf36:endothelial
hpf48:endothelial
Ontogeny
Macrophage
p_rht = 4.35e−07
TAI
2.20
2.18
2.16
2.14
2.12
2.10
2.08
2.06
2.04
2.02
2.00
1.98
hpf3.3:blastomere
hpf3.8:DEL
hpf4.3:margin
hpf4.8:margin
hpf5.3:non dorsal margin involuted
hpf6:non dorsal margin involuted
hpf7:non dorsal margin involuted
hpf8:mesoderm lateral plate
hpf9:mesoderm lateral plate
hpf10:mesoderm lateral plate
hpf11:mesoderm lateral plate (fli1a+)
hpf12:endothelial
hpf14:endothelial
hpf18:macrophage
hpf24:macrophage
hpf36:macrophage
hpf48:macrophage
Ontogeny
Erythroid
p_rht = 0.0207
TAI
2.20
2.15
2.10
2.05
2.00
1.95
hpf3.3:blastomere
hpf3.8:DEL
hpf4.3:margin
hpf4.8:margin
hpf5.3:non dorsal margin involuted
hpf6:non dorsal margin involuted
hpf7:non dorsal margin involuted
hpf8:mesoderm lateral plate
hpf9:mesoderm lateral plate
hpf10:mesoderm lateral plate
hpf11:mesoderm lateral plate (fli1a+)
hpf12:mesoderm lateral plate (fli1a+)
hpf14:blood island
hpf18:blood island
hpf24:erythroid
hpf36:erythroid
hpf48:erythroid
Ontogeny

Supp. Fig. 6. Reductive hourglass tests for the TAI profile of individual zebrafish cellular trajectories. Significance was assessed using 10,000 permutations, with a p-value ≤ 0.05 considered significant. The hourglass pattern was tested by defining early stages as hpf3.3-hpf12, mid as hpf14-hpf24, and late as hpf36-hpf24.

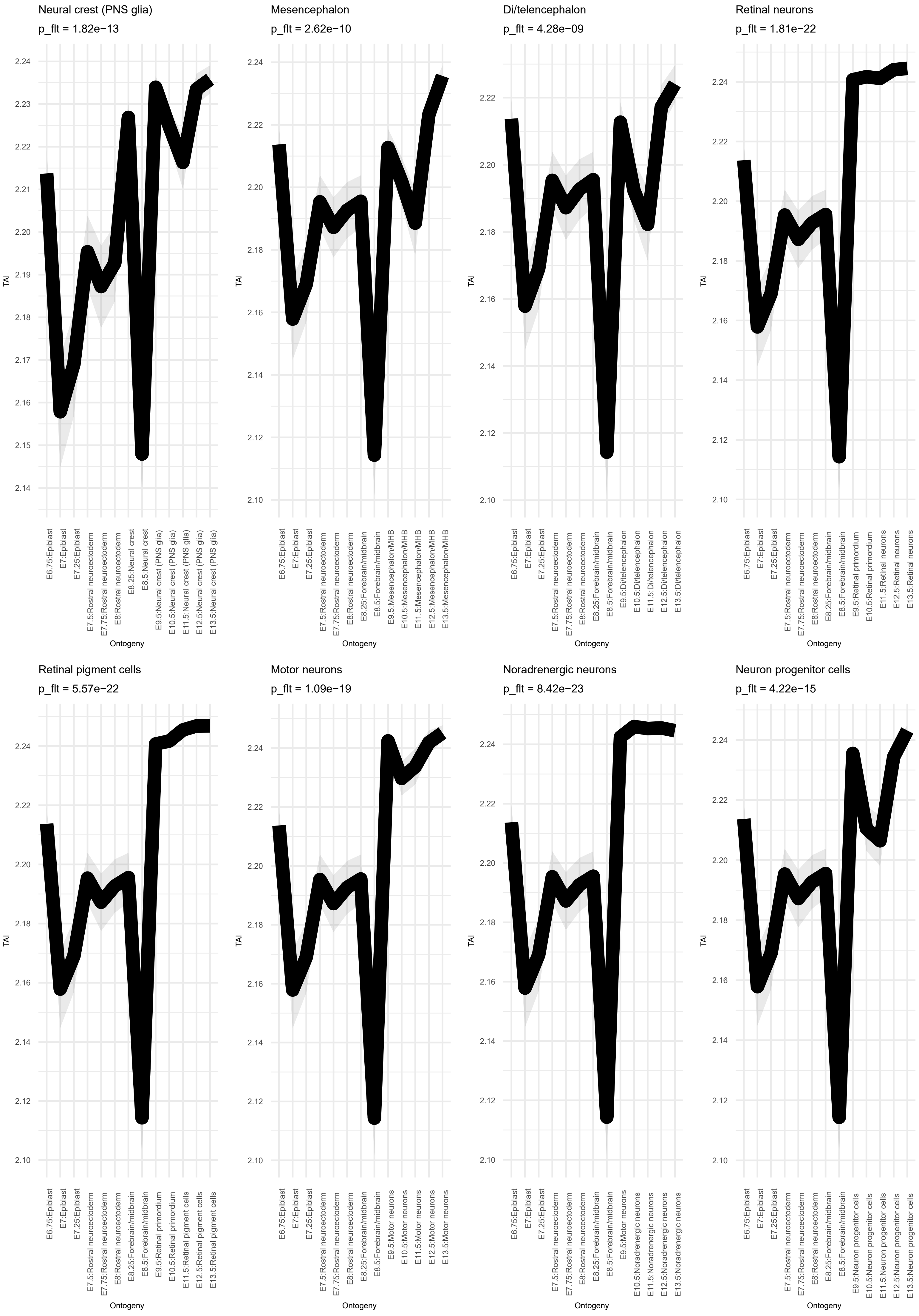

Neural crest (PNS glia)
p_flt = 1.82e−13
Mesencephalon
p_flt = 2.62e−10
Di/telencephalon
p_flt = 4.28e−09
Retinal neurons
p_flt = 1.81e−22
Retinal pigment cells
p_flt = 5.57e−22
Motor neurons
p_flt = 1.09e−19
Noradrenergic neurons
p_flt = 8.42e−23
Neuron progenitor cells
p_flt = 4.22e−15
TAI
Ontogeny

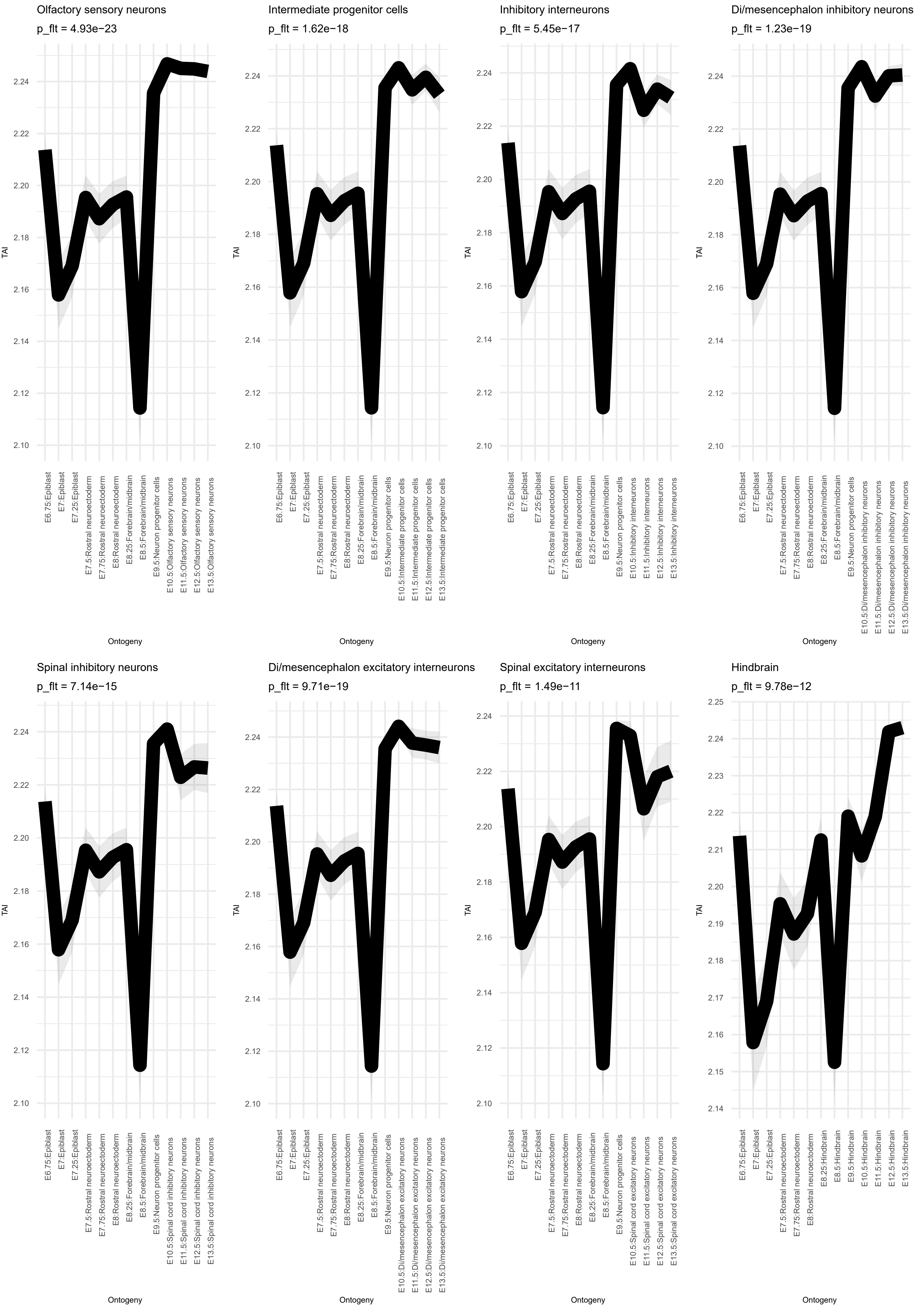

Olfactory sensory neurons
p_flt = 4.93e−23
Intermediate progenitor cells
p_flt = 1.62e−18
Inhibitory interneurons
p_flt = 5.45e−17
Di/mesencephalon inhibitory neurons
p_flt = 1.23e−19
Spinal inhibitory neurons
p_flt = 7.14e−15
Di/mesencephalon excitatory interneurons
p_flt = 9.71e−19
Spinal excitatory interneurons
p_flt = 1.49e−11
Hindbrain
p_flt = 9.78e−12
TAI
Ontogeny
E6.75:Epiblast
E7:Epiblast
E7.25:Epiblast
E7.5:Rostral neuroectoderm
E7.75:Rostral neuroectoderm
E8:Rostral neuroectoderm
E8.25:Forebrain/midbrain
E8.5:Forebrain/midbrain
E9.5:Neuron progenitor cells
E10.5:Olfactory sensory neurons
E11.5:Olfactory sensory neurons
E12.5:Olfactory sensory neurons
E13.5:Olfactory sensory neurons
E10.5:Intermediate progenitor cells
E11.5:Intermediate progenitor cells
E12.5:Intermediate progenitor cells
E13.5:Intermediate progenitor cells
E10.5:Inhibitory interneurons
E11.5:Inhibitory interneurons
E12.5:Inhibitory interneurons
E13.5:Inhibitory interneurons
E10.5:Di/mesencephalon inhibitory neurons
E11.5:Di/mesencephalon inhibitory neurons
E12.5:Di/mesencephalon inhibitory neurons
E13.5:Di/mesencephalon inhibitory neurons
E10.5:Spinal cord inhibitory neurons
E11.5:Spinal cord inhibitory neurons
E12.5:Spinal cord inhibitory neurons
E13.5:Spinal cord inhibitory neurons
E10.5:Di/mesencephalon excitatory neurons
E11.5:Di/mesencephalon excitatory neurons
E12.5:Di/mesencephalon excitatory neurons
E13.5:Di/mesencephalon excitatory neurons
E10.5:Spinal cord excitatory neurons
E11.5:Spinal cord excitatory neurons
E12.5:Spinal cord excitatory neurons
E13.5:Spinal cord excitatory neurons
E8.25:Hindbrain
E8.5:Hindbrain
E9.5:Hindbrain
E10.5:Hindbrain
E11.5:Hindbrain
E12.5:Hindbrain
E13.5:Hindbrain

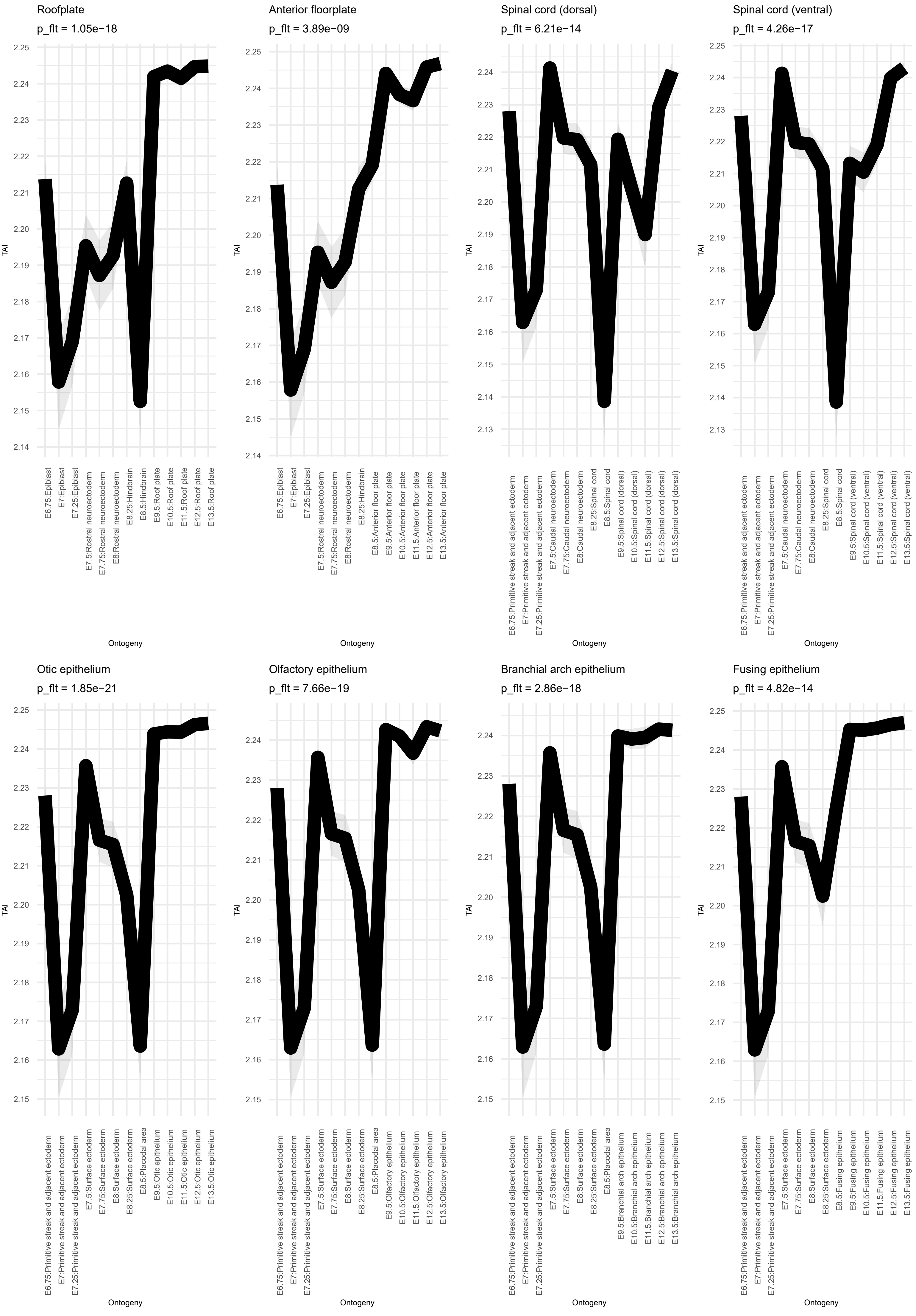

Roofplate
p_flt = 1.05e−18
TAI
Ontogeny
E6.75:Epiblast
E7:Epiblast
E7.25:Epiblast
E7.5:Rostral neuroectoderm
E7.75:Rostral neuroectoderm
E8:Rostral neuroectoderm
E8.25:Hindbrain
E8.5:Hindbrain
E9.5:Roof plate
E10.5:Roof plate
E11.5:Roof plate
E12.5:Roof plate
E13.5:Roof plate
Anterior floorplate
p_flt = 3.89e−09
TAI
Ontogeny
E6.75:Epiblast
E7:Epiblast
E7.25:Epiblast
E7.5:Rostral neuroectoderm
E7.75:Rostral neuroectoderm
E8:Rostral neuroectoderm
E8.25:Hindbrain
E8.5:Anterior floor plate
E9.5:Anterior floor plate
E10.5:Anterior floor plate
E11.5:Anterior floor plate
E12.5:Anterior floor plate
E13.5:Anterior floor plate
Spinal cord (dorsal)
p_flt = 6.21e−14
TAI
Ontogeny
E6.75:Primitive streak and adjacent ectoderm
E7:Primitive streak and adjacent ectoderm
E7.25:Primitive streak and adjacent ectoderm
E7.5:Caudal neuroectoderm
E7.75:Caudal neuroectoderm
E8:Caudal neuroectoderm
E8.25:Spinal cord
E8.5:Spinal cord
E9.5:Spinal cord (dorsal)
E10.5:Spinal cord (dorsal)
E11.5:Spinal cord (dorsal)
E12.5:Spinal cord (dorsal)
E13.5:Spinal cord (dorsal)
Spinal cord (ventral)
p_flt = 4.26e−17
TAI
Ontogeny
E6.75:Primitive streak and adjacent ectoderm
E7:Primitive streak and adjacent ectoderm
E7.25:Primitive streak and adjacent ectoderm
E7.5:Caudal neuroectoderm
E7.75:Caudal neuroectoderm
E8:Caudal neuroectoderm
E8.25:Spinal cord
E8.5:Spinal cord
E9.5:Spinal cord (ventral)
E10.5:Spinal cord (ventral)
E11.5:Spinal cord (ventral)
E12.5:Spinal cord (ventral)
E13.5:Spinal cord (ventral)
Otic epithelium
p_flt = 1.85e−21
TAI
Ontogeny
E6.75:Primitive streak and adjacent ectoderm
E7:Primitive streak and adjacent ectoderm
E7.25:Primitive streak and adjacent ectoderm
E7.5:Surface ectoderm
E7.75:Surface ectoderm
E8:Surface ectoderm
E8.25:Surface ectoderm
E8.5:Placodal area
E9.5:Otic epithelium
E10.5:Otic epithelium
E11.5:Otic epithelium
E12.5:Otic epithelium
E13.5:Otic epithelium
Olfactory epithelium
p_flt = 7.66e−19
TAI
Ontogeny
E6.75:Primitive streak and adjacent ectoderm
E7:Primitive streak and adjacent ectoderm
E7.25:Primitive streak and adjacent ectoderm
E7.5:Surface ectoderm
E7.75:Surface ectoderm
E8:Surface ectoderm
E8.25:Surface ectoderm
E8.5:Placodal area
E9.5:Olfactory epithelium
E10.5:Olfactory epithelium
E11.5:Olfactory epithelium
E12.5:Olfactory epithelium
E13.5:Olfactory epithelium
Branchial arch epithelium
p_flt = 2.86e−18
TAI
Ontogeny
E6.75:Primitive streak and adjacent ectoderm
E7:Primitive streak and adjacent ectoderm
E7.25:Primitive streak and adjacent ectoderm
E7.5:Surface ectoderm
E7.75:Surface ectoderm
E8:Surface ectoderm
E8.25:Surface ectoderm
E8.5:Placodal area
E9.5:Branchial arch epithelium
E10.5:Branchial arch epithelium
E11.5:Branchial arch epithelium
E12.5:Branchial arch epithelium
E13.5:Branchial arch epithelium
Fusing epithelium
p_flt = 4.82e−14
TAI
Ontogeny
E6.75:Primitive streak and adjacent ectoderm
E7:Primitive streak and adjacent ectoderm
E7.25:Primitive streak and adjacent ectoderm
E7.5:Surface ectoderm
E7.75:Surface ectoderm
E8:Surface ectoderm
E8.25:Surface ectoderm
E8.5:Fusing epithelium
E9.5:Fusing epithelium
E10.5:Fusing epithelium
E11.5:Fusing epithelium
E12.5:Fusing epithelium
E13.5:Fusing epithelium

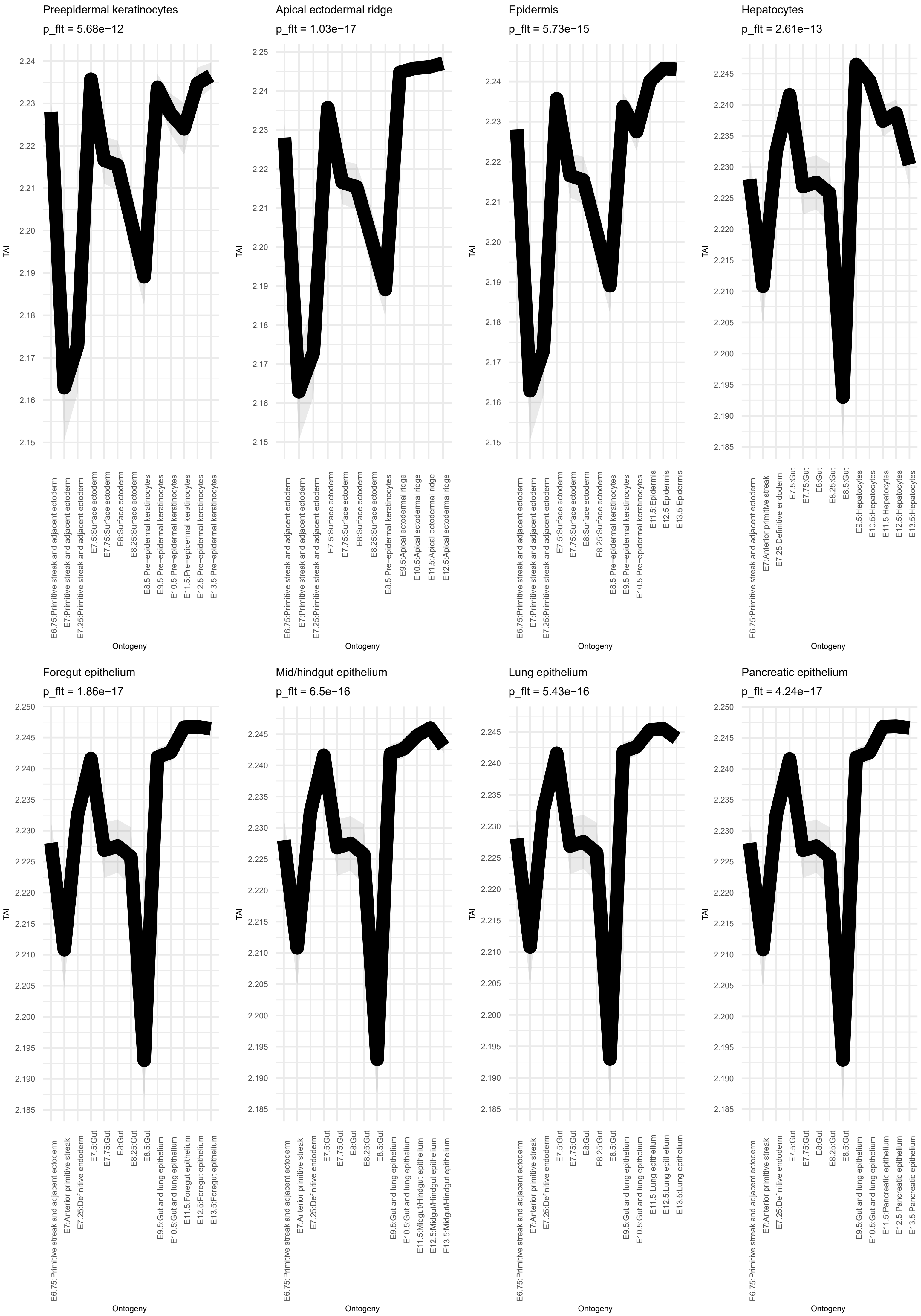

Preepidermal keratinocytes
p_flt = 5.68e−12
TAI
Ontogeny
E6.75:Primitive streak and adjacent ectoderm
E7:Primitive streak and adjacent ectoderm
E7.25:Primitive streak and adjacent ectoderm
E7.5:Surface ectoderm
E7.75:Surface ectoderm
E8:Surface ectoderm
E8.25:Surface ectoderm
E8.5:Pre−epidermal keratinocytes
E9.5:Pre−epidermal keratinocytes
E10.5:Pre−epidermal keratinocytes
E11.5:Pre−epidermal keratinocytes
E12.5:Pre−epidermal keratinocytes
E13.5:Pre−epidermal keratinocytes
Apical ectodermal ridge
p_flt = 1.03e−17
E9.5:Apical ectodermal ridge
E10.5:Apical ectodermal ridge
E11.5:Apical ectodermal ridge
E12.5:Apical ectodermal ridge
Epidermis
p_flt = 5.73e−15
E11.5:Epidermis
E12.5:Epidermis
E13.5:Epidermis
Hepatocytes
p_flt = 2.61e−13
E7:Anterior primitive streak
E7.25:Definitive endoderm
E7.5:Gut
E7.75:Gut
E8:Gut
E8.25:Gut
E8.5:Gut
E9.5:Hepatocytes
E10.5:Hepatocytes
E11.5:Hepatocytes
E12.5:Hepatocytes
E13.5:Hepatocytes
Foregut epithelium
p_flt = 1.86e−17
E9.5:Gut and lung epithelium
E10.5:Gut and lung epithelium
E11.5:Foregut epithelium
E12.5:Foregut epithelium
E13.5:Foregut epithelium
Mid/hindgut epithelium
p_flt = 6.5e−16
E11.5:Midgut/Hindgut epithelium
E12.5:Midgut/Hindgut epithelium
E13.5:Midgut/Hindgut epithelium
Lung epithelium
p_flt = 5.43e−16
E11.5:Lung epithelium
E12.5:Lung epithelium
E13.5:Lung epithelium
Pancreatic epithelium
p_flt = 4.24e−17
E11.5:Pancreatic epithelium
E12.5:Pancreatic epithelium
E13.5:Pancreatic epithelium

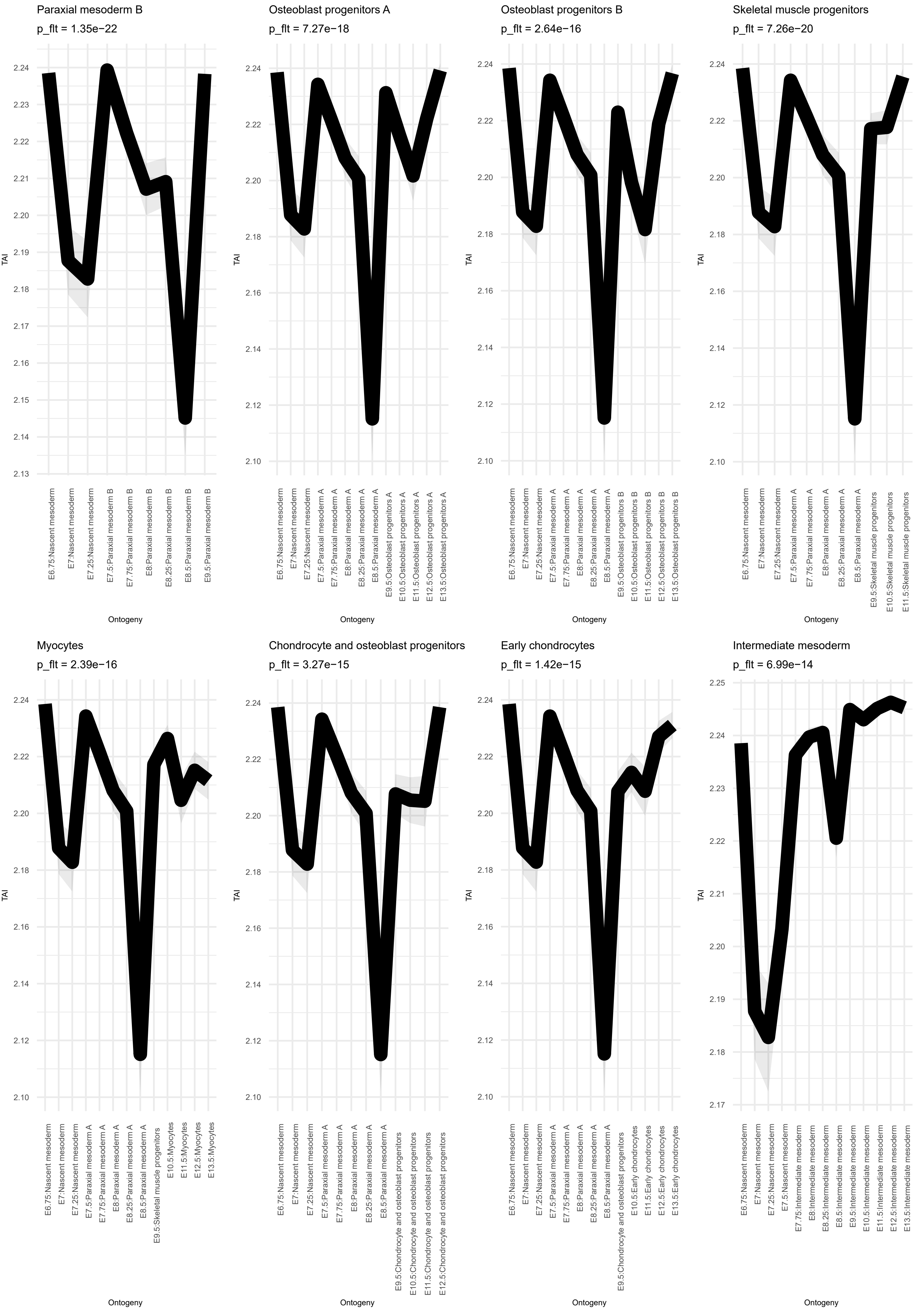
Paraxial mesoderm B
p_flt = 1.35e−22
Osteoblast progenitors A
p_flt = 7.27e−18
Osteoblast progenitors B
p_flt = 2.64e−16
Skeletal muscle progenitors
p_flt = 7.26e−20
Myocytes
p_flt = 2.39e−16
Chondrocyte and osteoblast progenitors
p_flt = 3.27e−15
Early chondrocytes
p_flt = 1.42e−15
Intermediate mesoderm
p_flt = 6.99e−14
TAI
Ontogeny
E6.75:Nascent mesoderm
E7:Nascent mesoderm
E7.25:Nascent mesoderm
E7.5:Paraxial mesoderm B
E7.75:Paraxial mesoderm B
E8:Paraxial mesoderm B
E8.25:Paraxial mesoderm B
E8.5:Paraxial mesoderm B
E9.5:Paraxial mesoderm B
E7.5:Paraxial mesoderm A
E7.75:Paraxial mesoderm A
E8:Paraxial mesoderm A
E8.25:Paraxial mesoderm A
E8.5:Paraxial mesoderm A
E9.5:Osteoblast progenitors A
E10.5:Osteoblast progenitors A
E11.5:Osteoblast progenitors A
E12.5:Osteoblast progenitors A
E13.5:Osteoblast progenitors A
E9.5:Osteoblast progenitors B
E10.5:Osteoblast progenitors B
E11.5:Osteoblast progenitors B
E12.5:Osteoblast progenitors B
E13.5:Osteoblast progenitors B
E9.5:Skeletal muscle progenitors
E10.5:Skeletal muscle progenitors
E11.5:Skeletal muscle progenitors
E10.5:Myocytes
E11.5:Myocytes
E12.5:Myocytes
E13.5:Myocytes
E9.5:Chondrocyte and osteoblast progenitors
E10.5:Chondrocyte and osteoblast progenitors
E11.5:Chondrocyte and osteoblast progenitors
E12.5:Chondrocyte and osteoblast progenitors
E10.5:Early chondrocytes
E11.5:Early chondrocytes
E12.5:Early chondrocytes
E13.5:Early chondrocytes
E7.5:Nascent mesoderm
E7.75:Intermediate mesoderm
E8:Intermediate mesoderm
E8.25:Intermediate mesoderm
E8.5:Intermediate mesoderm
E9.5:Intermediate mesoderm
E10.5:Intermediate mesoderm
E11.5:Intermediate mesoderm
E12.5:Intermediate mesoderm
E13.5:Intermediate mesoderm

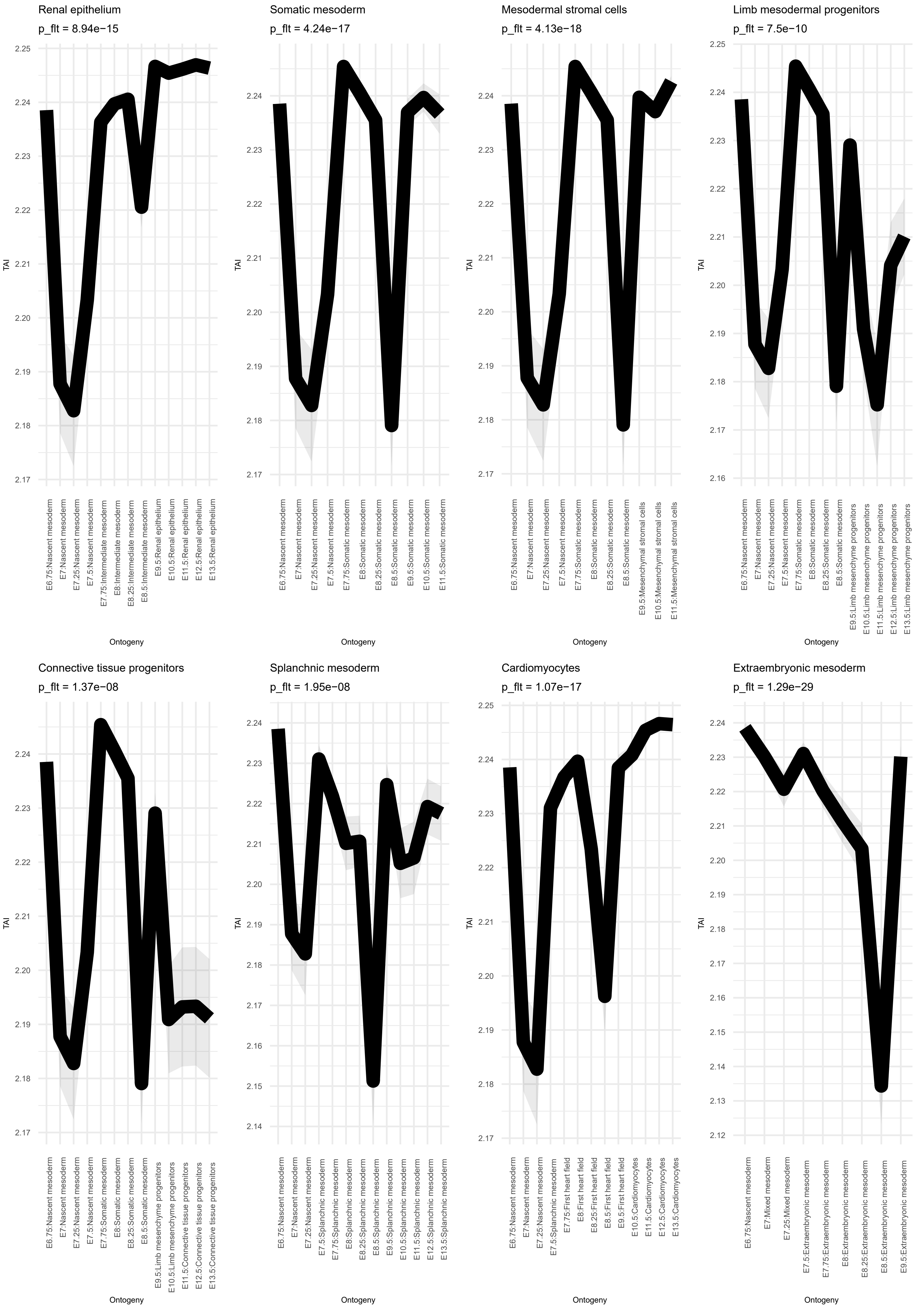
Renal epithelium
p_flt = 8.94e−15
Somatic mesoderm
p_flt = 4.24e−17
Mesodermal stromal cells
p_flt = 4.13e−18
Limb mesodermal progenitors
p_flt = 7.5e−10
Connective tissue progenitors
p_flt = 1.37e−08
Splanchnic mesoderm
p_flt = 1.95e−08
Cardiomyocytes
p_flt = 1.07e−17
Extraembryonic mesoderm
p_flt = 1.29e−29
TAI
Ontogeny

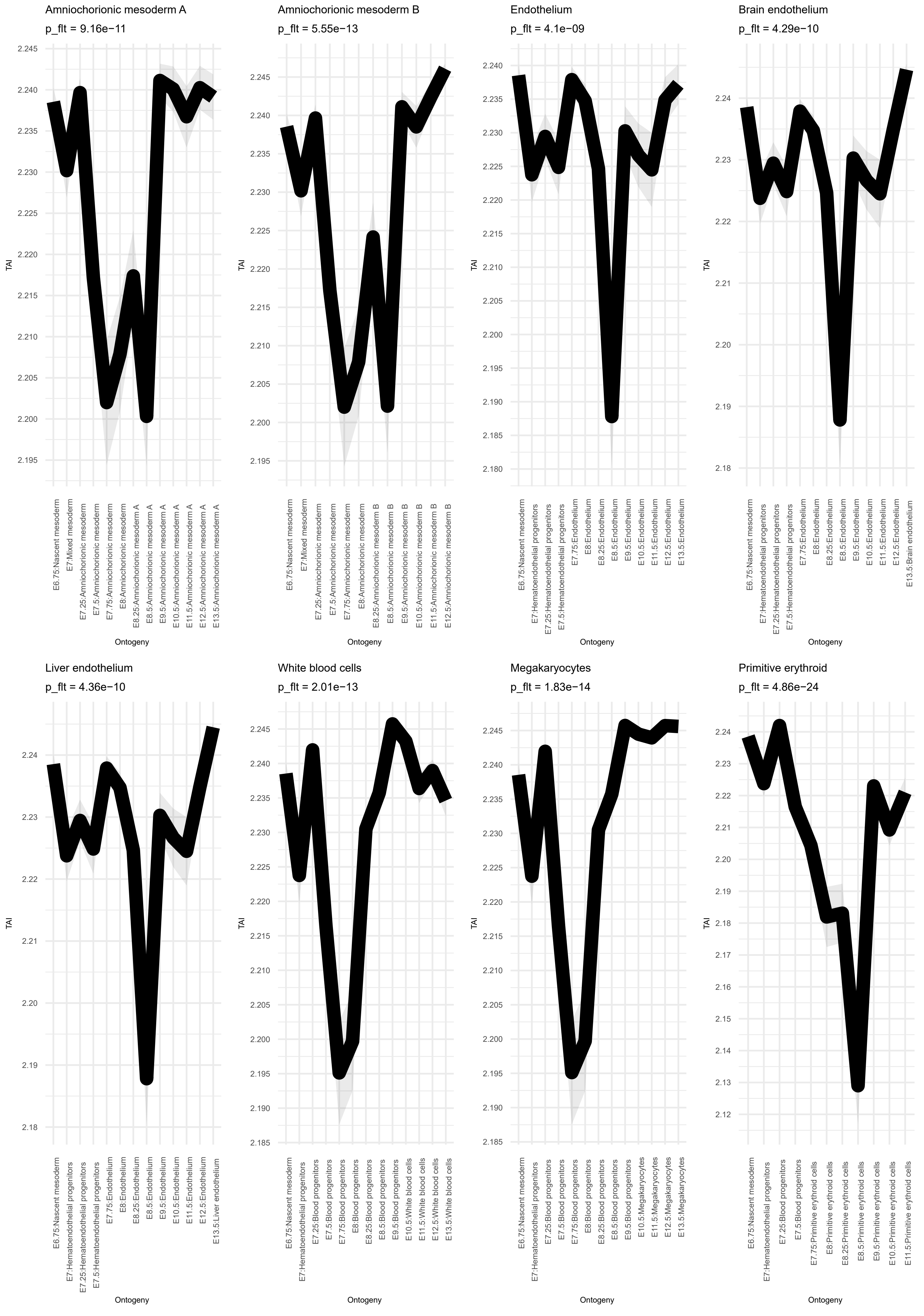
Amniochorionic mesoderm A
p_flt = 9.16e−11
TAI
2.195 2.200 2.205 2.210 2.215 2.220 2.225 2.230 2.235 2.240 2.245
E6.75:Nascent mesoderm
E7:Mixed mesoderm
E7.25:Amniochorionic mesoderm
E7.5:Amniochorionic mesoderm
E7.75:Amniochorionic mesoderm
E8:Amniochorionic mesoderm
E8.25:Amniochorionic mesoderm A
E8.5:Amniochorionic mesoderm A
E9.5:Amniochorionic mesoderm A
E10.5:Amniochorionic mesoderm A
E11.5:Amniochorionic mesoderm A
E12.5:Amniochorionic mesoderm A
E13.5:Amniochorionic mesoderm A
Ontogeny
Amniochorionic mesoderm B
p_flt = 5.55e−13
TAI
2.195 2.200 2.205 2.210 2.215 2.220 2.225 2.230 2.235 2.240 2.245
E6.75:Nascent mesoderm
E7:Mixed mesoderm
E7.25:Amniochorionic mesoderm
E7.5:Amniochorionic mesoderm
E7.75:Amniochorionic mesoderm
E8:Amniochorionic mesoderm
E8.25:Amniochorionic mesoderm B
E8.5:Amniochorionic mesoderm B
E9.5:Amniochorionic mesoderm B
E10.5:Amniochorionic mesoderm B
E11.5:Amniochorionic mesoderm B
E12.5:Amniochorionic mesoderm B
Ontogeny
Endothelium
p_flt = 4.1e−09
TAI
2.180 2.185 2.190 2.195 2.200 2.205 2.210 2.215 2.220 2.225 2.230 2.235 2.240
E6.75:Nascent mesoderm
E7:Hematoendothelial progenitors
E7.25:Hematoendothelial progenitors
E7.5:Hematoendothelial progenitors
E7.75:Endothelium
E8:Endothelium
E8.25:Endothelium
E8.5:Endothelium
E9.5:Endothelium
E10.5:Endothelium
E11.5:Endothelium
E12.5:Endothelium
E13.5:Endothelium
Ontogeny
Brain endothelium
p_flt = 4.29e−10
TAI
2.18 2.19 2.20 2.21 2.22 2.23 2.24
E6.75:Nascent mesoderm
E7:Hematoendothelial progenitors
E7.25:Hematoendothelial progenitors
E7.5:Hematoendothelial progenitors
E7.75:Endothelium
E8:Endothelium
E8.25:Endothelium
E8.5:Endothelium
E9.5:Endothelium
E10.5:Endothelium
E11.5:Endothelium
E12.5:Endothelium
E13.5:Brain endothelium
Ontogeny
Liver endothelium
p_flt = 4.36e−10
TAI
2.18 2.19 2.20 2.21 2.22 2.23 2.24
E6.75:Nascent mesoderm
E7:Hematoendothelial progenitors
E7.25:Hematoendothelial progenitors
E7.5:Hematoendothelial progenitors
E7.75:Endothelium
E8:Endothelium
E8.25:Endothelium
E8.5:Endothelium
E9.5:Endothelium
E10.5:Endothelium
E11.5:Endothelium
E12.5:Endothelium
E13.5:Liver endothelium
Ontogeny
White blood cells
p_flt = 2.01e−13
TAI
2.185 2.190 2.195 2.200 2.205 2.210 2.215 2.220 2.225 2.230 2.235 2.240 2.245
E6.75:Nascent mesoderm
E7:Hematoendothelial progenitors
E7.25:Blood progenitors
E7.5:Blood progenitors
E7.75:Blood progenitors
E8:Blood progenitors
E8.25:Blood progenitors
E8.5:Blood progenitors
E9.5:Blood progenitors
E10.5:White blood cells
E11.5:White blood cells
E12.5:White blood cells
E13.5:White blood cells
Ontogeny
Megakaryocytes
p_flt = 1.83e−14
TAI
2.185 2.190 2.195 2.200 2.205 2.210 2.215 2.220 2.225 2.230 2.235 2.240 2.245
E6.75:Nascent mesoderm
E7:Hematoendothelial progenitors
E7.25:Blood progenitors
E7.5:Blood progenitors
E7.75:Blood progenitors
E8:Blood progenitors
E8.25:Blood progenitors
E8.5:Blood progenitors
E9.5:Blood progenitors
E10.5:Megakaryocytes
E11.5:Megakaryocytes
E12.5:Megakaryocytes
E13.5:Megakaryocytes
Ontogeny
Primitive erythroid
p_flt = 4.86e−24
TAI
2.12 2.13 2.14 2.15 2.16 2.17 2.18 2.19 2.20 2.21 2.22 2.23 2.24
E6.75:Nascent mesoderm
E7:Hematoendothelial progenitors
E7.25:Blood progenitors
E7.5:Blood progenitors
E7.75:Primitive erythroid cells
E8:Primitive erythroid cells
E8.25:Primitive erythroid cells
E8.5:Primitive erythroid cells
E9.5:Primitive erythroid cells
E10.5:Primitive erythroid cells
E11.5:Primitive erythroid cells
Ontogeny

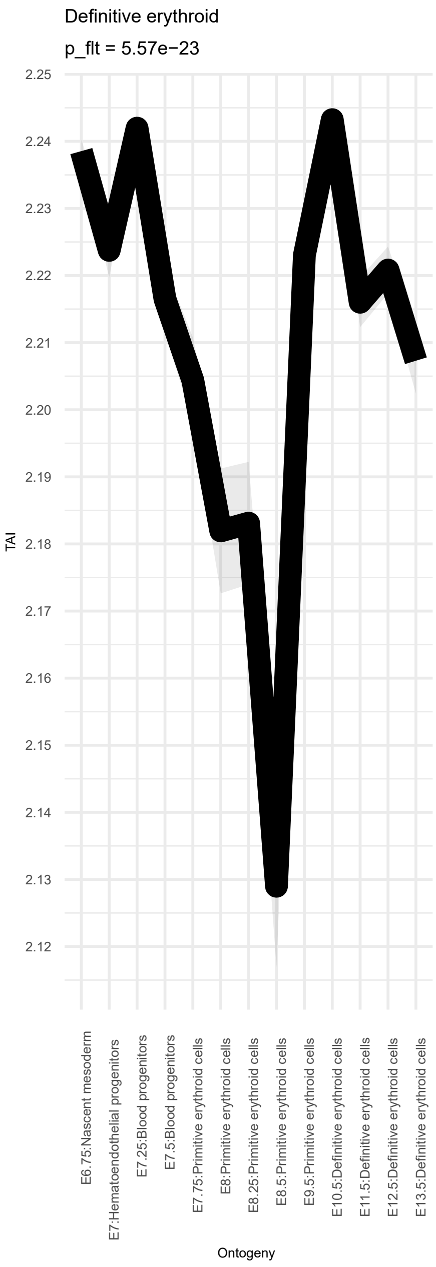

Supp. Fig. 7. Flatline tests for the TAI profile of individual mouse cellular trajectories using a denoised dataset. Significance was assessed using 10,000 permutations, with a p-value ≤ 0.05 considered significant.

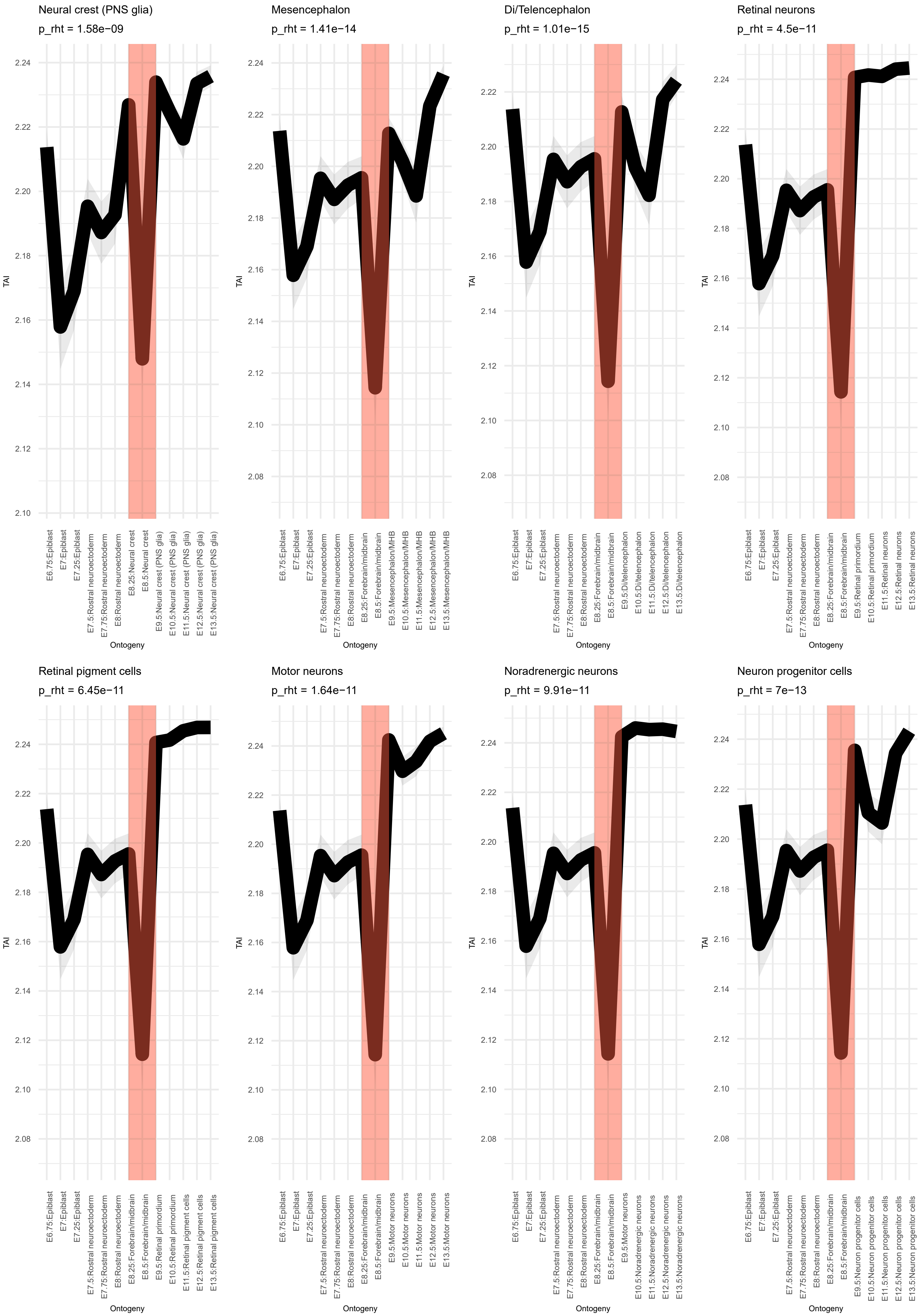

Neural crest (PNS glia)
p_rht = 1.58e−09
TAI
2.10 2.12 2.14 2.16 2.18 2.20 2.22 2.24
E6.75:Epiblast
E7:Epiblast
E7.25:Epiblast
E7.5:Rostral neuroectoderm
E7.75:Rostral neuroectoderm
E8:Rostral neuroectoderm
E8.25:Neural crest
E8.5:Neural crest
E9.5:Neural crest (PNS glia)
E10.5:Neural crest (PNS glia)
E11.5:Neural crest (PNS glia)
E12.5:Neural crest (PNS glia)
E13.5:Neural crest (PNS glia)
Ontogeny
Mesencephalon
p_rht = 1.41e−14
TAI
2.08 2.10 2.12 2.14 2.16 2.18 2.20 2.22 2.24
E6.75:Epiblast
E7:Epiblast
E7.25:Epiblast
E7.5:Rostral neuroectoderm
E7.75:Rostral neuroectoderm
E8:Rostral neuroectoderm
E8.25:Forebrain/midbrain
E8.5:Forebrain/midbrain
E9.5:Mesencephalon/MHB
E10.5:Mesencephalon/MHB
E11.5:Mesencephalon/MHB
E12.5:Mesencephalon/MHB
E13.5:Mesencephalon/MHB
Ontogeny
Di/Telencephalon
p_rht = 1.01e−15
TAI
2.08 2.10 2.12 2.14 2.16 2.18 2.20 2.22
E6.75:Epiblast
E7:Epiblast
E7.25:Epiblast
E7.5:Rostral neuroectoderm
E7.75:Rostral neuroectoderm
E8:Rostral neuroectoderm
E8.25:Forebrain/midbrain
E8.5:Forebrain/midbrain
E9.5:Di/telencephalon
E10.5:Di/telencephalon
E11.5:Di/telencephalon
E12.5:Di/telencephalon
E13.5:Di/telencephalon
Ontogeny
Retinal neurons
p_rht = 4.5e−11
TAI
2.08 2.10 2.12 2.14 2.16 2.18 2.20 2.22 2.24
E6.75:Epiblast
E7:Epiblast
E7.25:Epiblast
E7.5:Rostral neuroectoderm
E7.75:Rostral neuroectoderm
E8:Rostral neuroectoderm
E8.25:Forebrain/midbrain
E8.5:Forebrain/midbrain
E9.5:Retinal primordium
E10.5:Retinal primordium
E11.5:Retinal neurons
E12.5:Retinal neurons
E13.5:Retinal neurons
Ontogeny
Retinal pigment cells
p_rht = 6.45e−11
TAI
2.08 2.10 2.12 2.14 2.16 2.18 2.20 2.22 2.24
E6.75:Epiblast
E7:Epiblast
E7.25:Epiblast
E7.5:Rostral neuroectoderm
E7.75:Rostral neuroectoderm
E8:Rostral neuroectoderm
E8.25:Forebrain/midbrain
E8.5:Forebrain/midbrain
E9.5:Retinal primordium
E10.5:Retinal primordium
E11.5:Retinal pigment cells
E12.5:Retinal pigment cells
E13.5:Retinal pigment cells
Ontogeny
Motor neurons
p_rht = 1.64e−11
TAI
2.08 2.10 2.12 2.14 2.16 2.18 2.20 2.22 2.24
E6.75:Epiblast
E7:Epiblast
E7.25:Epiblast
E7.5:Rostral neuroectoderm
E7.75:Rostral neuroectoderm
E8:Rostral neuroectoderm
E8.25:Forebrain/midbrain
E8.5:Forebrain/midbrain
E9.5:Motor neurons
E10.5:Motor neurons
E11.5:Motor neurons
E12.5:Motor neurons
E13.5:Motor neurons
Ontogeny
Noradrenergic neurons
p_rht = 9.91e−11
TAI
2.08 2.10 2.12 2.14 2.16 2.18 2.20 2.22 2.24
E6.75:Epiblast
E7:Epiblast
E7.25:Epiblast
E7.5:Rostral neuroectoderm
E7.75:Rostral neuroectoderm
E8:Rostral neuroectoderm
E8.25:Forebrain/midbrain
E8.5:Forebrain/midbrain
E9.5:Motor neurons
E10.5:Noradrenergic neurons
E11.5:Noradrenergic neurons
E12.5:Noradrenergic neurons
E13.5:Noradrenergic neurons
Ontogeny
Neuron progenitor cells
p_rht = 7e−13
TAI
2.08 2.10 2.12 2.14 2.16 2.18 2.20 2.22 2.24
E6.75:Epiblast
E7:Epiblast
E7.25:Epiblast
E7.5:Rostral neuroectoderm
E7.75:Rostral neuroectoderm
E8:Rostral neuroectoderm
E8.25:Forebrain/midbrain
E8.5:Forebrain/midbrain
E9.5:Neuron progenitor cells
E10.5:Neuron progenitor cells
E11.5:Neuron progenitor cells
E12.5:Neuron progenitor cells
E13.5:Neuron progenitor cells
Ontogeny

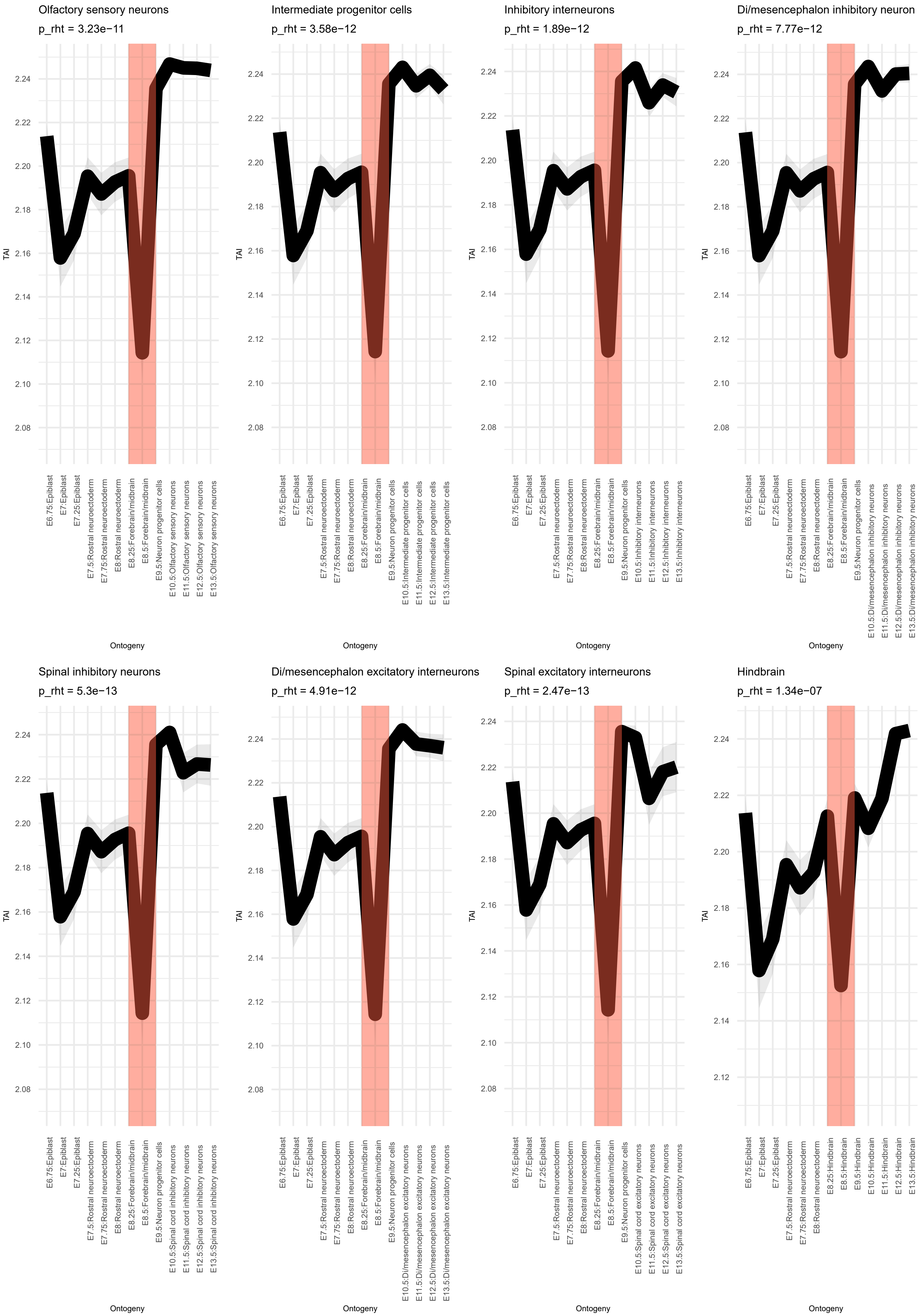

Olfactory sensory neurons
p_rht = 3.23e−11
Intermediate progenitor cells
p_rht = 3.58e−12
Inhibitory interneurons
p_rht = 1.89e−12
Di/mesencephalon inhibitory neuron
p_rht = 7.77e−12
Spinal inhibitory neurons
p_rht = 5.3e−13
Di/mesencephalon excitatory interneurons
p_rht = 4.91e−12
Spinal excitatory interneurons
p_rht = 2.47e−13
Hindbrain
p_rht = 1.34e−07
TAI
Ontogeny

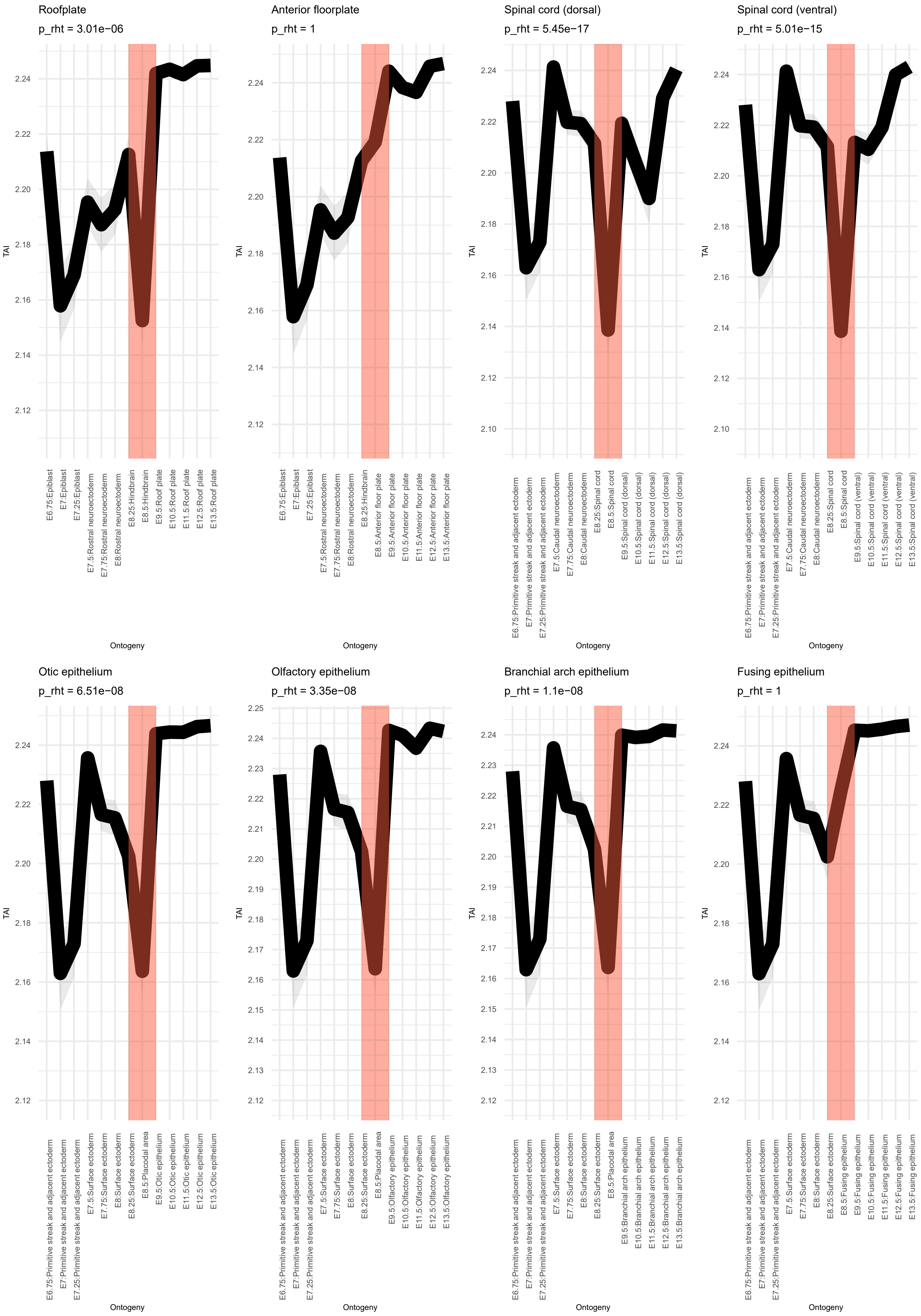

Roofplate
p_rht = 3.01e−06
TAI
2.12
2.14
2.16
2.18
2.20
2.22
2.24
E6.75:Epiblast
E7:Epiblast
E7.25:Epiblast
E7.5:Rostral neuroectoderm
E7.75:Rostral neuroectoderm
E8:Rostral neuroectoderm
E8.25:Hindbrain
E8.5:Hindbrain
E9.5:Roof plate
E10.5:Roof plate
E11.5:Roof plate
E12.5:Roof plate
E13.5:Roof plate
Ontogeny
Anterior floorplate
p_rht = 1
TAI
2.12
2.14
2.16
2.18
2.20
2.22
2.24
E6.75:Epiblast
E7:Epiblast
E7.25:Epiblast
E7.5:Rostral neuroectoderm
E7.75:Rostral neuroectoderm
E8:Rostral neuroectoderm
E8.25:Hindbrain
E8.5:Anterior floor plate
E9.5:Anterior floor plate
E10.5:Anterior floor plate
E11.5:Anterior floor plate
E12.5:Anterior floor plate
E13.5:Anterior floor plate
Ontogeny
Spinal cord (dorsal)
p_rht = 5.45e−17
TAI
2.10
2.12
2.14
2.16
2.18
2.20
2.22
2.24
E6.75:Primitive streak and adjacent ectoderm
E7:Primitive streak and adjacent ectoderm
E7.25:Primitive streak and adjacent ectoderm
E7.5:Caudal neuroectoderm
E7.75:Caudal neuroectoderm
E8:Caudal neuroectoderm
E8.25:Spinal cord
E8.5:Spinal cord
E9.5:Spinal cord (dorsal)
E10.5:Spinal cord (dorsal)
E11.5:Spinal cord (dorsal)
E12.5:Spinal cord (dorsal)
E13.5:Spinal cord (dorsal)
Ontogeny
Spinal cord (ventral)
p_rht = 5.01e−15
TAI
2.10
2.12
2.14
2.16
2.18
2.20
2.22
2.24
E6.75:Primitive streak and adjacent ectoderm
E7:Primitive streak and adjacent ectoderm
E7.25:Primitive streak and adjacent ectoderm
E7.5:Caudal neuroectoderm
E7.75:Caudal neuroectoderm
E8:Caudal neuroectoderm
E8.25:Spinal cord
E8.5:Spinal cord
E9.5:Spinal cord (ventral)
E10.5:Spinal cord (ventral)
E11.5:Spinal cord (ventral)
E12.5:Spinal cord (ventral)
E13.5:Spinal cord (ventral)
Ontogeny
Otic epithelium
p_rht = 6.51e−08
TAI
2.12
2.14
2.16
2.18
2.20
2.22
2.24
E6.75:Primitive streak and adjacent ectoderm
E7:Primitive streak and adjacent ectoderm
E7.25:Primitive streak and adjacent ectoderm
E7.5:Surface ectoderm
E7.75:Surface ectoderm
E8:Surface ectoderm
E8.25:Surface ectoderm
E8.5:Placodal area
E9.5:Otic epithelium
E10.5:Otic epithelium
E11.5:Otic epithelium
E12.5:Otic epithelium
E13.5:Otic epithelium
Ontogeny
Olfactory epithelium
p_rht = 3.35e−08
TAI
2.12
2.13
2.14
2.15
2.16
2.17
2.18
2.19
2.20
2.21
2.22
2.23
2.24
2.25
E6.75:Primitive streak and adjacent ectoderm
E7:Primitive streak and adjacent ectoderm
E7.25:Primitive streak and adjacent ectoderm
E7.5:Surface ectoderm
E7.75:Surface ectoderm
E8:Surface ectoderm
E8.25:Surface ectoderm
E8.5:Placodal area
E9.5:Olfactory epithelium
E10.5:Olfactory epithelium
E11.5:Olfactory epithelium
E12.5:Olfactory epithelium
E13.5:Olfactory epithelium
Ontogeny
Branchial arch epithelium
p_rht = 1.1e−08
TAI
2.12
2.13
2.14
2.15
2.16
2.17
2.18
2.19
2.20
2.21
2.22
2.23
2.24
E6.75:Primitive streak and adjacent ectoderm
E7:Primitive streak and adjacent ectoderm
E7.25:Primitive streak and adjacent ectoderm
E7.5:Surface ectoderm
E7.75:Surface ectoderm
E8:Surface ectoderm
E8.25:Surface ectoderm
E8.5:Placodal area
E9.5:Branchial arch epithelium
E10.5:Branchial arch epithelium
E11.5:Branchial arch epithelium
E12.5:Branchial arch epithelium
E13.5:Branchial arch epithelium
Ontogeny
Fusing epithelium
p_rht = 1
TAI
2.12
2.14
2.16
2.18
2.20
2.22
2.24
E6.75:Primitive streak and adjacent ectoderm
E7:Primitive streak and adjacent ectoderm
E7.25:Primitive streak and adjacent ectoderm
E7.5:Surface ectoderm
E7.75:Surface ectoderm
E8:Surface ectoderm
E8.25:Surface ectoderm
E8.5:Fusing epithelium
E9.5:Fusing epithelium
E10.5:Fusing epithelium
E11.5:Fusing epithelium
E12.5:Fusing epithelium
E13.5:Fusing epithelium
Ontogeny

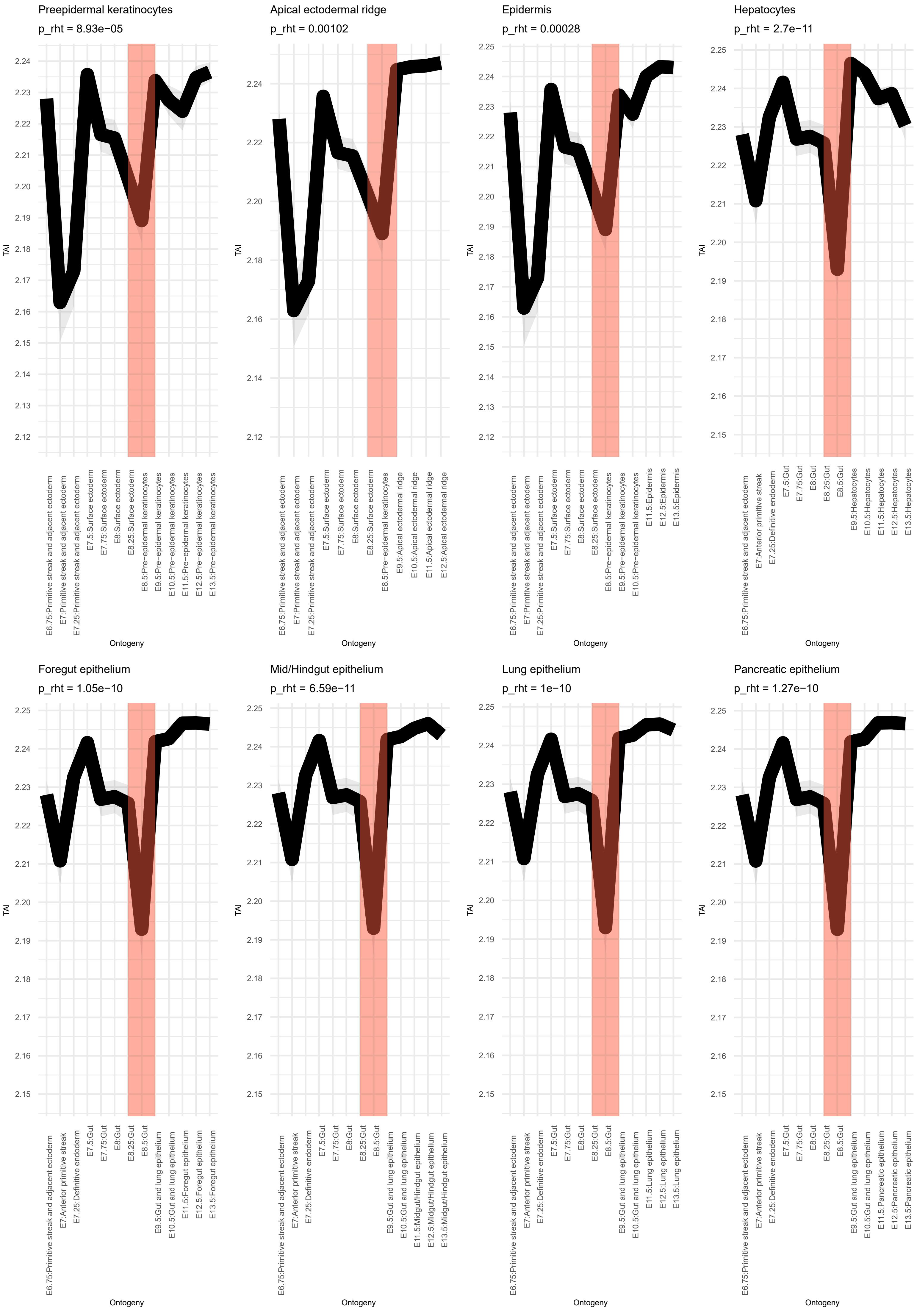
Preepidermal keratinocytes
p_rht = 8.93e−05
Apical ectodermal ridge
p_rht = 0.00102
Epidermis
p_rht = 0.00028
Hepatocytes
p_rht = 2.7e−11
Foregut epithelium
p_rht = 1.05e−10
Mid/Hindgut epithelium
p_rht = 6.59e−11
Lung epithelium
p_rht = 1e−10
Pancreatic epithelium
p_rht = 1.27e−10
TAI
Ontogeny

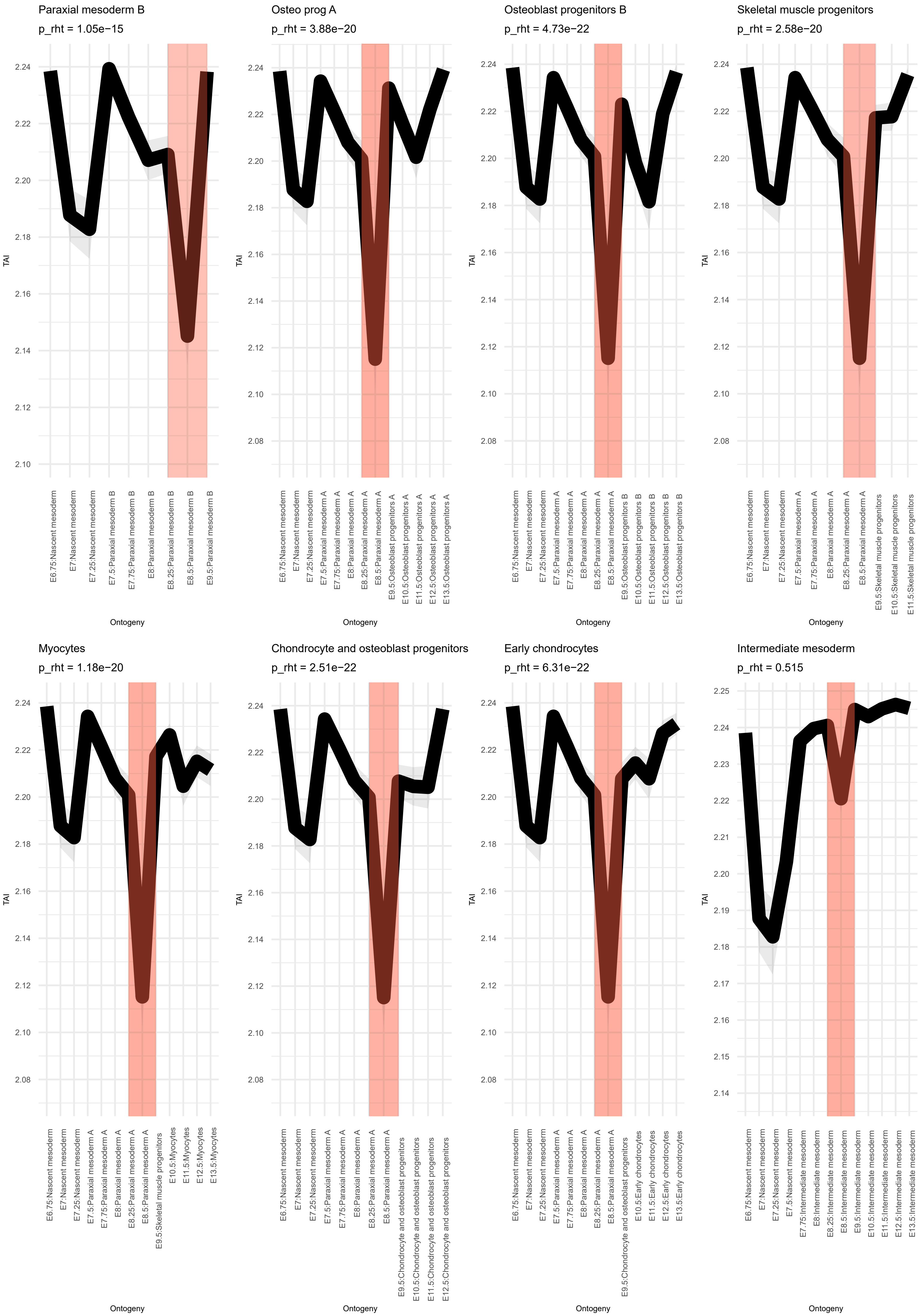
Paraxial mesoderm B
p_rht = 1.05e−15
Osteo prog A
p_rht = 3.88e−20
Osteoblast progenitors B
p_rht = 4.73e−22
Skeletal muscle progenitors
p_rht = 2.58e−20
Myocytes
p_rht = 1.18e−20
Chondrocyte and osteoblast progenitors
p_rht = 2.51e−22
Early chondrocytes
p_rht = 6.31e−22
Intermediate mesoderm
p_rht = 0.515
TAI
Ontogeny
E6.75:Nascent mesoderm
E7:Nascent mesoderm
E7.25:Nascent mesoderm
E7.5:Paraxial mesoderm B
E7.75:Paraxial mesoderm B
E8:Paraxial mesoderm B
E8.25:Paraxial mesoderm B
E8.5:Paraxial mesoderm B
E9.5:Paraxial mesoderm B
E7.5:Paraxial mesoderm A
E7.75:Paraxial mesoderm A
E8:Paraxial mesoderm A
E8.25:Paraxial mesoderm A
E8.5:Paraxial mesoderm A
E9.5:Osteoblast progenitors A
E10.5:Osteoblast progenitors A
E11.5:Osteoblast progenitors A
E12.5:Osteoblast progenitors A
E13.5:Osteoblast progenitors A
E9.5:Osteoblast progenitors B
E10.5:Osteoblast progenitors B
E11.5:Osteoblast progenitors B
E12.5:Osteoblast progenitors B
E13.5:Osteoblast progenitors B
E9.5:Skeletal muscle progenitors
E10.5:Skeletal muscle progenitors
E11.5:Skeletal muscle progenitors
E10.5:Myocytes
E11.5:Myocytes
E12.5:Myocytes
E13.5:Myocytes
E9.5:Chondrocyte and osteoblast progenitors
E10.5:Chondrocyte and osteoblast progenitors
E11.5:Chondrocyte and osteoblast progenitors
E12.5:Chondrocyte and osteoblast progenitors
E10.5:Early chondrocytes
E11.5:Early chondrocytes
E12.5:Early chondrocytes
E13.5:Early chondrocytes
E7.75:Intermediate mesoderm
E8:Intermediate mesoderm
E8.25:Intermediate mesoderm
E8.5:Intermediate mesoderm
E9.5:Intermediate mesoderm
E10.5:Intermediate mesoderm
E11.5:Intermediate mesoderm
E12.5:Intermediate mesoderm
E13.5:Intermediate mesoderm

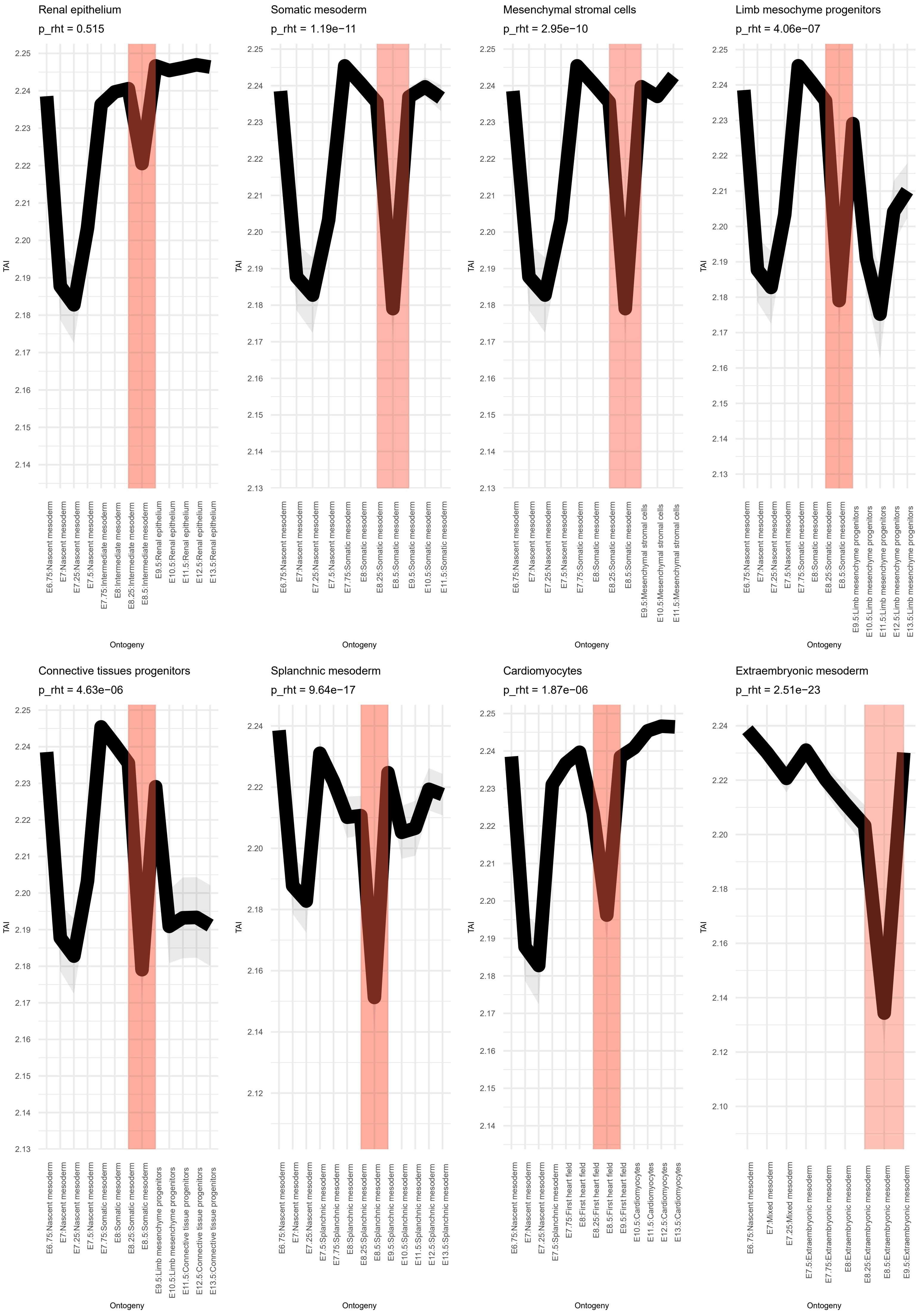
Renal epithelium
p_rht = 0.515
TAI
2.25
2.24
2.23
2.22
2.21
2.20
2.19
2.18
2.17
2.16
2.15
2.14
E6.75:Nascent mesoderm
E7:Nascent mesoderm
E7.25:Nascent mesoderm
E7.5:Nascent mesoderm
E7.75:Intermediate mesoderm
E8:Intermediate mesoderm
E8.25:Intermediate mesoderm
E8.5:Intermediate mesoderm
E9.5:Renal epithelium
E10.5:Renal epithelium
E11.5:Renal epithelium
E12.5:Renal epithelium
E13.5:Renal epithelium
Ontogeny
Somatic mesoderm
p_rht = 1.19e−11
TAI
2.25
2.24
2.23
2.22
2.21
2.20
2.19
2.18
2.17
2.16
2.15
2.14
2.13
E6.75:Nascent mesoderm
E7:Nascent mesoderm
E7.25:Nascent mesoderm
E7.5:Nascent mesoderm
E7.75:Somatic mesoderm
E8:Somatic mesoderm
E8.25:Somatic mesoderm
E8.5:Somatic mesoderm
E9.5:Somatic mesoderm
E10.5:Somatic mesoderm
E11.5:Somatic mesoderm
Ontogeny
Mesenchymal stromal cells
p_rht = 2.95e−10
TAI
2.25
2.24
2.23
2.22
2.21
2.20
2.19
2.18
2.17
2.16
2.15
2.14
2.13
E6.75:Nascent mesoderm
E7:Nascent mesoderm
E7.25:Nascent mesoderm
E7.5:Nascent mesoderm
E7.75:Somatic mesoderm
E8:Somatic mesoderm
E8.25:Somatic mesoderm
E8.5:Somatic mesoderm
E9.5:Mesenchymal stromal cells
E10.5:Mesenchymal stromal cells
E11.5:Mesenchymal stromal cells
Ontogeny
Limb mesochyme progenitors
p_rht = 4.06e−07
TAI
2.25
2.24
2.23
2.22
2.21
2.20
2.19
2.18
2.17
2.16
2.15
2.14
2.13
E6.75:Nascent mesoderm
E7:Nascent mesoderm
E7.25:Nascent mesoderm
E7.5:Nascent mesoderm
E7.75:Somatic mesoderm
E8:Somatic mesoderm
E8.25:Somatic mesoderm
E8.5:Somatic mesoderm
E9.5:Limb mesenchyme progenitors
E10.5:Limb mesenchyme progenitors
E11.5:Limb mesenchyme progenitors
E12.5:Limb mesenchyme progenitors
E13.5:Limb mesenchyme progenitors
Ontogeny
Connective tissues progenitors
p_rht = 4.63e−06
TAI
2.25
2.24
2.23
2.22
2.21
2.20
2.19
2.18
2.17
2.16
2.15
2.14
2.13
E6.75:Nascent mesoderm
E7:Nascent mesoderm
E7.25:Nascent mesoderm
E7.5:Nascent mesoderm
E7.75:Somatic mesoderm
E8:Somatic mesoderm
E8.25:Somatic mesoderm
E8.5:Somatic mesoderm
E9.5:Limb mesenchyme progenitors
E10.5:Limb mesenchyme progenitors
E11.5:Connective tissue progenitors
E12.5:Connective tissue progenitors
E13.5:Connective tissue progenitors
Ontogeny
Splanchnic mesoderm
p_rht = 9.64e−17
TAI
2.24
2.22
2.20
2.18
2.16
2.14
2.12
E6.75:Nascent mesoderm
E7:Nascent mesoderm
E7.25:Nascent mesoderm
E7.5:Splanchnic mesoderm
E7.75:Splanchnic mesoderm
E8:Splanchnic mesoderm
E8.25:Splanchnic mesoderm
E8.5:Splanchnic mesoderm
E9.5:Splanchnic mesoderm
E10.5:Splanchnic mesoderm
E11.5:Splanchnic mesoderm
E12.5:Splanchnic mesoderm
E13.5:Splanchnic mesoderm
Ontogeny
Cardiomyocytes
p_rht = 1.87e−06
TAI
2.25
2.24
2.23
2.22
2.21
2.20
2.19
2.18
2.17
2.16
2.15
2.14
E6.75:Nascent mesoderm
E7:Nascent mesoderm
E7.25:Nascent mesoderm
E7.5:Splanchnic mesoderm
E7.75:First heart field
E8:First heart field
E8.25:First heart field
E8.5:First heart field
E9.5:First heart field
E10.5:Cardiomyocytes
E11.5:Cardiomyocytes
E12.5:Cardiomyocytes
E13.5:Cardiomyocytes
Ontogeny
Extraembryonic mesoderm
p_rht = 2.51e−23
TAI
2.24
2.22
2.20
2.18
2.16
2.14
2.12
2.10
E6.75:Nascent mesoderm
E7:Mixed mesoderm
E7.25:Mixed mesoderm
E7.5:Extraembryonic mesoderm
E7.75:Extraembryonic mesoderm
E8:Extraembryonic mesoderm
E8.25:Extraembryonic mesoderm
E8.5:Extraembryonic mesoderm
E9.5:Extraembryonic mesoderm
Ontogeny

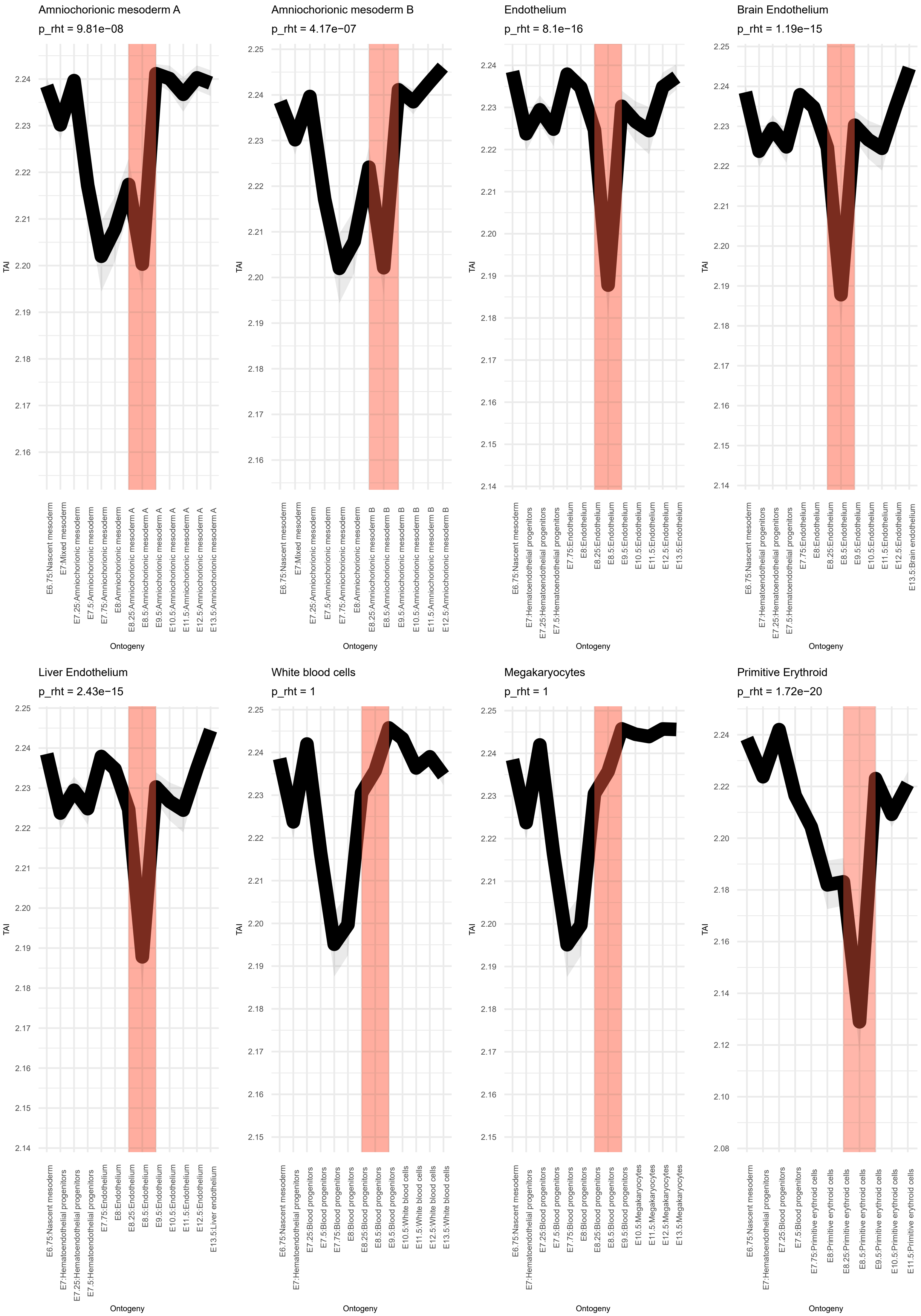
Amniochorionic mesoderm A
p_rht = 9.81e−08
TAI
2.24
2.23
2.22
2.21
2.20
2.19
2.18
2.17
2.16
E6.75:Nascent mesoderm
E7:Mixed mesoderm
E7.25:Amniochorionic mesoderm
E7.5:Amniochorionic mesoderm
E7.75:Amniochorionic mesoderm
E8:Amniochorionic mesoderm
E8.25:Amniochorionic mesoderm A
E8.5:Amniochorionic mesoderm A
E9.5:Amniochorionic mesoderm A
E10.5:Amniochorionic mesoderm A
E11.5:Amniochorionic mesoderm A
E12.5:Amniochorionic mesoderm A
E13.5:Amniochorionic mesoderm A
Ontogeny
Amniochorionic mesoderm B
p_rht = 4.17e−07
TAI
2.25
2.24
2.23
2.22
2.21
2.20
2.19
2.18
2.17
2.16
E6.75:Nascent mesoderm
E7:Mixed mesoderm
E7.25:Amniochorionic mesoderm
E7.5:Amniochorionic mesoderm
E7.75:Amniochorionic mesoderm
E8:Amniochorionic mesoderm
E8.25:Amniochorionic mesoderm B
E8.5:Amniochorionic mesoderm B
E9.5:Amniochorionic mesoderm B
E10.5:Amniochorionic mesoderm B
E11.5:Amniochorionic mesoderm B
E12.5:Amniochorionic mesoderm B
Ontogeny
Endothelium
p_rht = 8.1e−16
TAI
2.24
2.23
2.22
2.21
2.20
2.19
2.18
2.17
2.16
2.15
2.14
E6.75:Nascent mesoderm
E7:Hematoendothelial progenitors
E7.25:Hematoendothelial progenitors
E7.5:Hematoendothelial progenitors
E7.75:Endothelium
E8:Endothelium
E8.25:Endothelium
E8.5:Endothelium
E9.5:Endothelium
E10.5:Endothelium
E11.5:Endothelium
E12.5:Endothelium
E13.5:Endothelium
Ontogeny
Brain Endothelium
p_rht = 1.19e−15
TAI
2.25
2.24
2.23
2.22
2.21
2.20
2.19
2.18
2.17
2.16
2.15
2.14
E6.75:Nascent mesoderm
E7:Hematoendothelial progenitors
E7.25:Hematoendothelial progenitors
E7.5:Hematoendothelial progenitors
E7.75:Endothelium
E8:Endothelium
E8.25:Endothelium
E8.5:Endothelium
E9.5:Endothelium
E10.5:Endothelium
E11.5:Endothelium
E12.5:Endothelium
E13.5:Brain endothelium
Ontogeny
Liver Endothelium
p_rht = 2.43e−15
TAI
2.25
2.24
2.23
2.22
2.21
2.20
2.19
2.18
2.17
2.16
2.15
2.14
E6.75:Nascent mesoderm
E7:Hematoendothelial progenitors
E7.25:Hematoendothelial progenitors
E7.5:Hematoendothelial progenitors
E7.75:Endothelium
E8:Endothelium
E8.25:Endothelium
E8.5:Endothelium
E9.5:Endothelium
E10.5:Endothelium
E11.5:Endothelium
E12.5:Endothelium
E13.5:Liver endothelium
Ontogeny
White blood cells
p_rht = 1
TAI
2.25
2.24
2.23
2.22
2.21
2.20
2.19
2.18
2.17
2.16
2.15
E6.75:Nascent mesoderm
E7:Hematoendothelial progenitors
E7.25:Blood progenitors
E7.5:Blood progenitors
E7.75:Blood progenitors
E8:Blood progenitors
E8.25:Blood progenitors
E8.5:Blood progenitors
E9.5:Blood progenitors
E10.5:White blood cells
E11.5:White blood cells
E12.5:White blood cells
E13.5:White blood cells
Ontogeny
Megakaryocytes
p_rht = 1
TAI
2.25
2.24
2.23
2.22
2.21
2.20
2.19
2.18
2.17
2.16
2.15
E6.75:Nascent mesoderm
E7:Hematoendothelial progenitors
E7.25:Blood progenitors
E7.5:Blood progenitors
E7.75:Blood progenitors
E8:Blood progenitors
E8.25:Blood progenitors
E8.5:Blood progenitors
E9.5:Blood progenitors
E10.5:Megakaryocytes
E11.5:Megakaryocytes
E12.5:Megakaryocytes
E13.5:Megakaryocytes
Ontogeny
Primitive Erythroid
p_rht = 1.72e−20
TAI
2.24
2.22
2.20
2.18
2.16
2.14
2.12
2.10
2.08
E6.75:Nascent mesoderm
E7:Hematoendothelial progenitors
E7.25:Blood progenitors
E7.5:Blood progenitors
E7.75:Primitive erythroid cells
E8:Primitive erythroid cells
E8.25:Primitive erythroid cells
E8.5:Primitive erythroid cells
E9.5:Primitive erythroid cells
E10.5:Primitive erythroid cells
E11.5:Primitive erythroid cells
Ontogeny

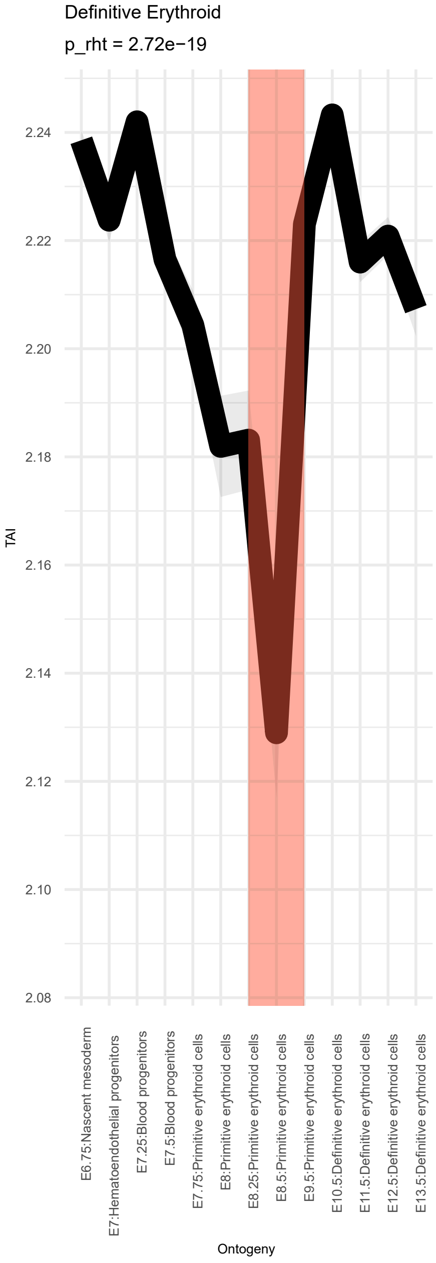

Supp. Fig. 8. Reductive hourglass tests for the TAI profile of individual mouse cellular trajectories using a denoised dataset. Significance was assessed using 10,000 permutations, with a p-value $\leq$ 0.05 considered significant. The hourglass pattern was tested by defining early stages as E6.75–E8.25, mid as E8.5, and late as E9.5–E13.5.

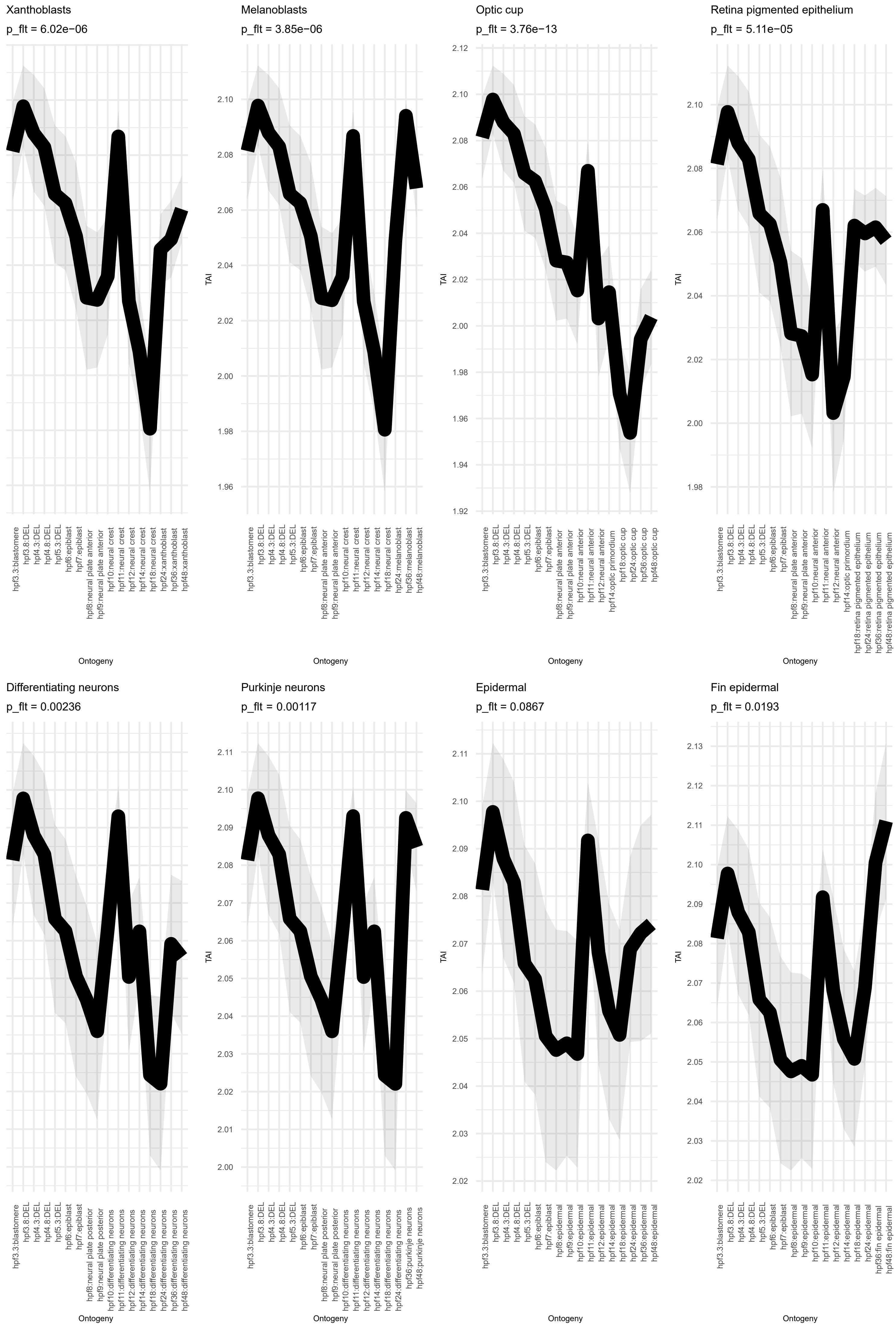
Xanthoblasts
p_flt = 6.02e−06
Melanoblasts
p_flt = 3.85e−06
Optic cup
p_flt = 3.76e−13
Retina pigmented epithelium
p_flt = 5.11e−05
Differentiating neurons
p_flt = 0.00236
Purkinje neurons
p_flt = 0.00117
Epidermal
p_flt = 0.0867
Fin epidermal
p_flt = 0.0193
TAI
Ontogeny
hpf3.3:blastomere
hpf3.8:DEL
hpf4.3:DEL
hpf4.8:DEL
hpf5.3:DEL
hpf6:epiblast
hpf7:epiblast
hpf8:neural plate anterior
hpf9:neural plate anterior
hpf10:neural crest
hpf11:neural crest
hpf12:neural crest
hpf14:neural crest
hpf18:neural crest
hpf24:xanthoblast
hpf36:xanthoblast
hpf48:xanthoblast
hpf24:melanoblast
hpf36:melanoblast
hpf48:melanoblast
hpf10:neural anterior
hpf11:neural anterior
hpf12:neural anterior
hpf14:optic primordium
hpf18:optic cup
hpf24:optic cup
hpf36:optic cup
hpf48:optic cup
hpf18:retina pigmented epithelium
hpf24:retina pigmented epithelium
hpf36:retina pigmented epithelium
hpf48:retina pigmented epithelium
hpf8:neural plate posterior
hpf9:neural plate posterior
hpf10:differentiating neurons
hpf11:differentiating neurons
hpf12:differentiating neurons
hpf14:differentiating neurons
hpf18:differentiating neurons
hpf24:differentiating neurons
hpf36:differentiating neurons
hpf48:differentiating neurons
hpf36:purkinje neurons
hpf48:purkinje neurons
hpf8:epidermal
hpf9:epidermal
hpf10:epidermal
hpf11:epidermal
hpf12:epidermal
hpf14:epidermal
hpf18:epidermal
hpf24:epidermal
hpf36:epidermal
hpf48:epidermal
hpf36:fin epidermal
hpf48:fin epidermal

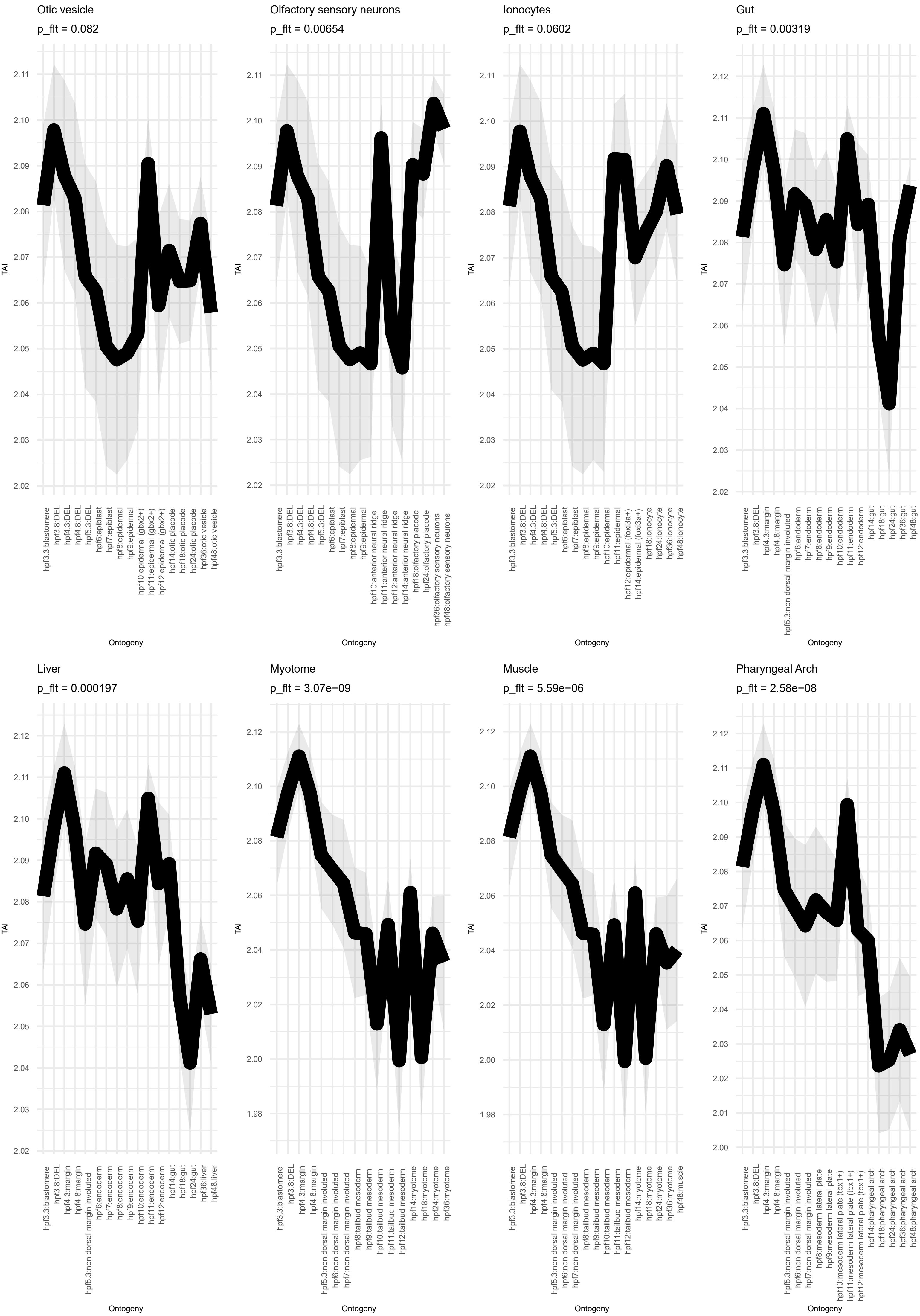
Otic vesicle
p_flt = 0.082
TAI
Ontogeny
hpf3.3:blastomere
hpf3.8:DEL
hpf4.3:DEL
hpf4.8:DEL
hpf5.3:DEL
hpf6:epiblast
hpf7:epiblast
hpf8:epidermal
hpf9:epidermal
hpf10:epidermal (gbx2+)
hpf11:epidermal (gbx2+)
hpf12:epidermal (gbx2+)
hpf14:otic placode
hpf18:otic placode
hpf24:otic placode
hpf36:otic vesicle
hpf48:otic vesicle
Olfactory sensory neurons
p_flt = 0.00654
hpf10:anterior neural ridge
hpf11:anterior neural ridge
hpf12:anterior neural ridge
hpf14:anterior neural ridge
hpf18:olfactory placode
hpf24:olfactory placode
hpf36:olfactory sensory neurons
hpf48:olfactory sensory neurons
Ionocytes
p_flt = 0.0602
hpf10:epidermal
hpf11:epidermal
hpf12:epidermal (foxi3a+)
hpf14:epidermal (foxi3a+)
hpf18:ionocyte
hpf24:ionocyte
hpf36:ionocyte
hpf48:ionocyte
Gut
p_flt = 0.00319
hpf4.3:margin
hpf4.8:margin
hpf5.3:non dorsal margin involuted
hpf6:endoderm
hpf7:endoderm
hpf8:endoderm
hpf9:endoderm
hpf10:endoderm
hpf11:endoderm
hpf12:endoderm
hpf14:gut
hpf18:gut
hpf24:gut
hpf36:gut
hpf48:gut
Liver
p_flt = 0.000197
hpf36:liver
hpf48:liver
Myotome
p_flt = 3.07e−09
hpf6:non dorsal margin involuted
hpf7:non dorsal margin involuted
hpf8:tailbud mesoderm
hpf9:tailbud mesoderm
hpf10:tailbud mesoderm
hpf11:tailbud mesoderm
hpf12:tailbud mesoderm
hpf14:myotome
hpf18:myotome
hpf24:myotome
hpf36:myotome
Muscle
p_flt = 5.59e−06
hpf48:muscle
Pharyngeal Arch
p_flt = 2.58e−08
hpf8:mesoderm lateral plate
hpf9:mesoderm lateral plate
hpf10:mesoderm lateral plate (tbx1+)
hpf11:mesoderm lateral plate (tbx1+)
hpf12:mesoderm lateral plate (tbx1+)
hpf14:pharyngeal arch
hpf18:pharyngeal arch
hpf24:pharyngeal arch
hpf36:pharyngeal arch
hpf48:pharyngeal arch

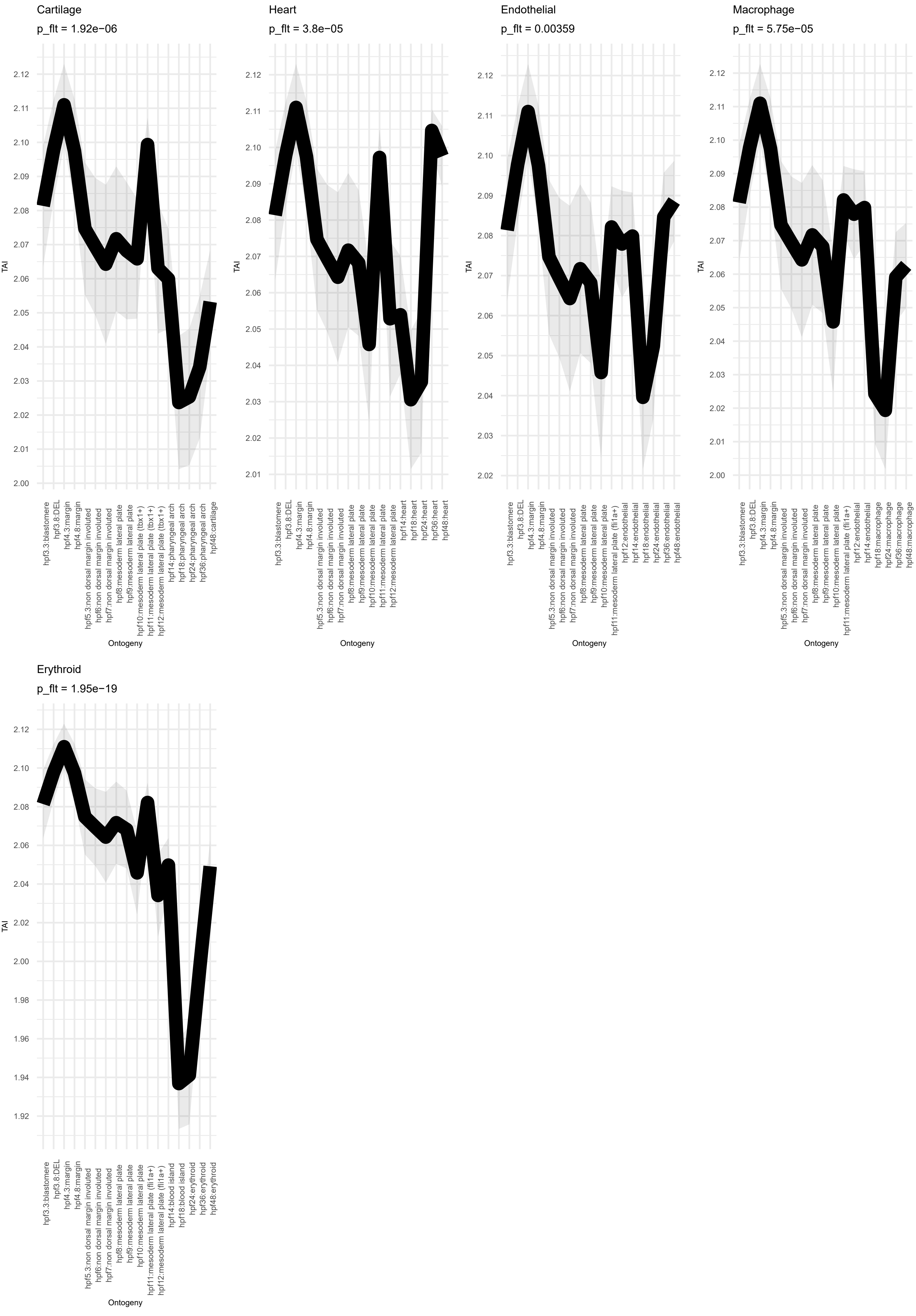

Cartilage
p_flt = 1.92e−06
Heart
p_flt = 3.8e−05
Endothelial
p_flt = 0.00359
Macrophage
p_flt = 5.75e−05
Erythroid
p_flt = 1.95e−19
TAI
Ontogeny
hpf3.3:blastomere
hpf3.8:DEL
hpf4.3:margin
hpf4.8:margin
hpf5.3:non dorsal margin involuted
hpf6:non dorsal margin involuted
hpf7:non dorsal margin involuted
hpf8:mesoderm lateral plate
hpf9:mesoderm lateral plate
hpf10:mesoderm lateral plate (tbx1+)
hpf11:mesoderm lateral plate (tbx1+)
hpf12:mesoderm lateral plate (tbx1+)
hpf14:pharyngeal arch
hpf18:pharyngeal arch
hpf24:pharyngeal arch
hpf36:pharyngeal arch
hpf48:cartilage
hpf10:mesoderm lateral plate
hpf11:mesoderm lateral plate
hpf12:mesoderm lateral plate
hpf14:heart
hpf18:heart
hpf24:heart
hpf36:heart
hpf48:heart
hpf11:mesoderm lateral plate (fli1a+)
hpf12:endothelial
hpf14:endothelial
hpf18:endothelial
hpf24:endothelial
hpf36:endothelial
hpf48:endothelial
hpf10:mesoderm lateral plate (fli1a+)
hpf14:macrophage
hpf18:macrophage
hpf24:macrophage
hpf36:macrophage
hpf48:macrophage
hpf12:mesoderm lateral plate (fli1a+)
hpf14:blood island
hpf18:blood island
hpf24:erythroid
hpf36:erythroid
hpf48:erythroid

Supp. Fig. 9. Flatline tests for the TAI profile of individual zebrafish cellular trajectories using a denoised dataset. Significance was assessed using 10,000 permutations, with a p-value ≤ 0.05 considered significant.

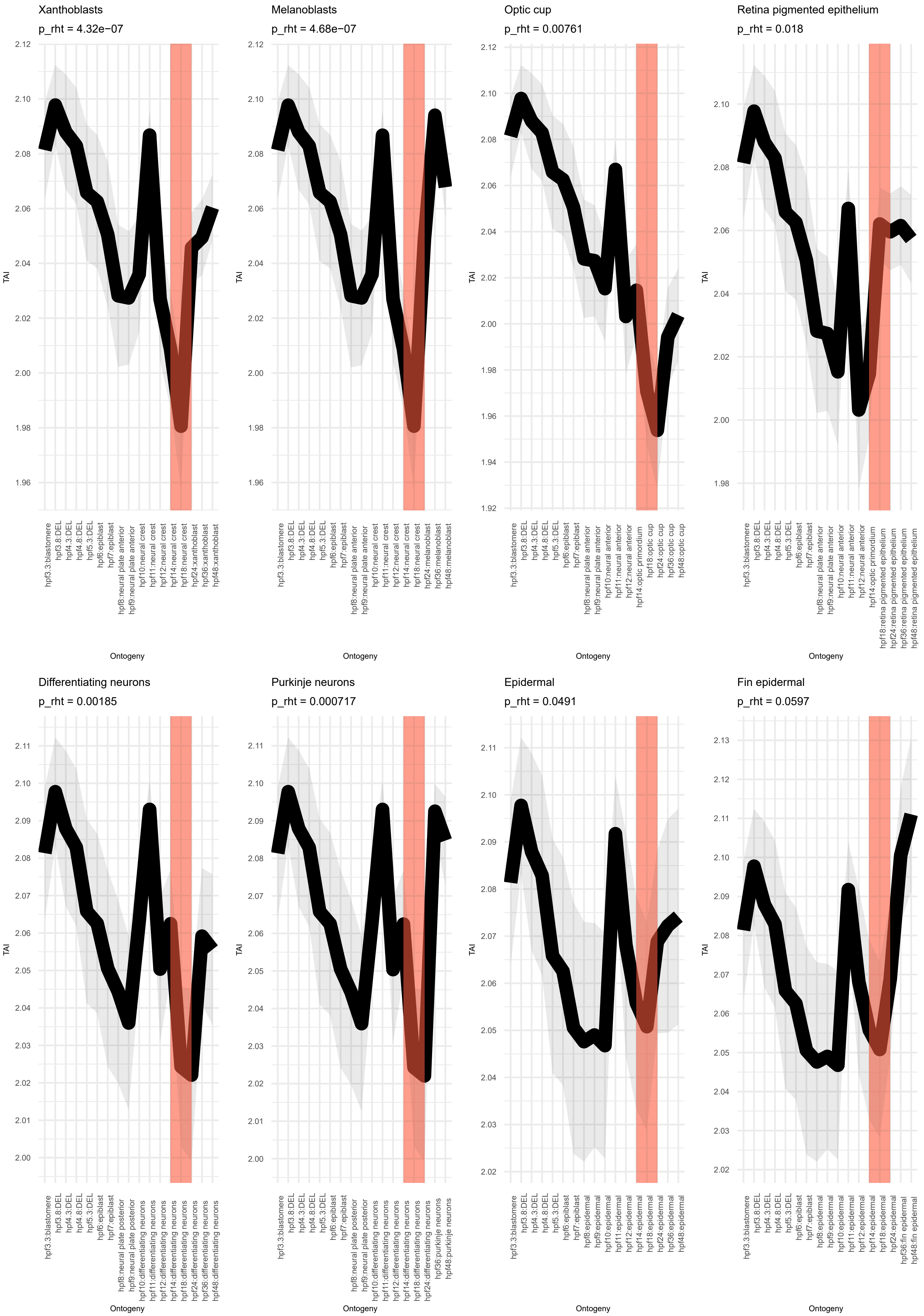
Xanthoblasts
p_rht = 4.32e−07
Melanoblasts
p_rht = 4.68e−07
Optic cup
p_rht = 0.00761
Retina pigmented epithelium
p_rht = 0.018
Differentiating neurons
p_rht = 0.00185
Purkinje neurons
p_rht = 0.000717
Epidermal
p_rht = 0.0491
Fin epidermal
p_rht = 0.0597
TAI
Ontogeny

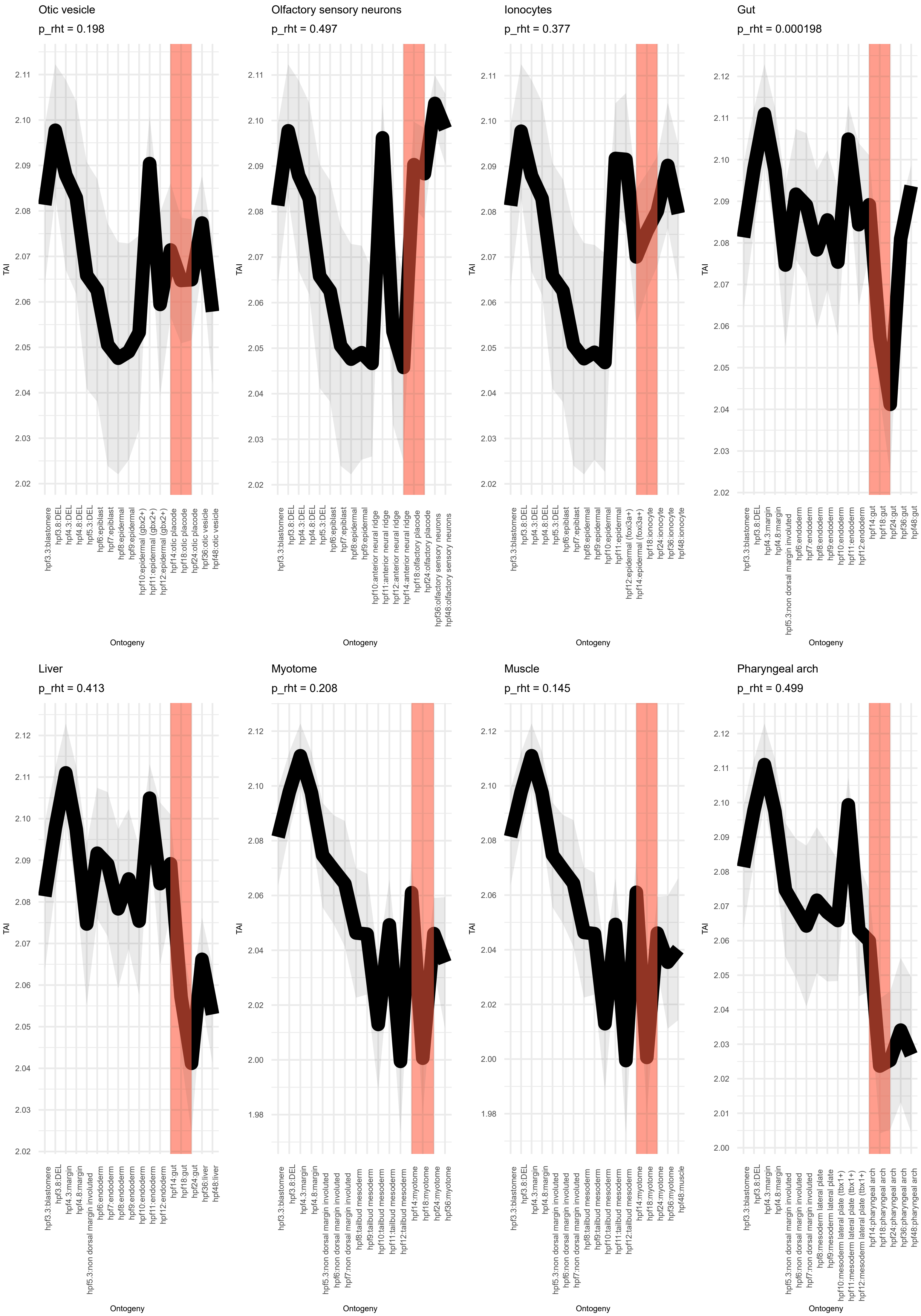

Otic vesicle
p_rht = 0.198
TAI
Ontogeny
hpf3.3:blastomere
hpf3.8:DEL
hpf4.3:DEL
hpf4.8:DEL
hpf5.3:DEL
hpf6:epiblast
hpf7:epiblast
hpf8:epidermal
hpf9:epidermal
hpf10:epidermal (gbx2+)
hpf11:epidermal (gbx2+)
hpf12:epidermal (gbx2+)
hpf14:otic placode
hpf18:otic placode
hpf24:otic placode
hpf36:otic vesicle
hpf48:otic vesicle
Olfactory sensory neurons
p_rht = 0.497
hpf10:anterior neural ridge
hpf11:anterior neural ridge
hpf12:anterior neural ridge
hpf14:anterior neural ridge
hpf18:olfactory placode
hpf24:olfactory placode
hpf36:olfactory sensory neurons
hpf48:olfactory sensory neurons
Ionocytes
p_rht = 0.377
hpf10:epidermal
hpf11:epidermal
hpf12:epidermal (foxi3a+)
hpf14:epidermal (foxi3a+)
hpf18:ionocyte
hpf24:ionocyte
hpf36:ionocyte
hpf48:ionocyte
Gut
p_rht = 0.000198
hpf4.3:margin
hpf4.8:margin
hpf5.3:non dorsal margin involuted
hpf6:endoderm
hpf7:endoderm
hpf8:endoderm
hpf9:endoderm
hpf10:endoderm
hpf11:endoderm
hpf12:endoderm
hpf14:gut
hpf18:gut
hpf24:gut
hpf36:gut
hpf48:gut
Liver
p_rht = 0.413
hpf36:liver
hpf48:liver
Myotome
p_rht = 0.208
hpf6:non dorsal margin involuted
hpf7:non dorsal margin involuted
hpf8:tailbud mesoderm
hpf9:tailbud mesoderm
hpf10:tailbud mesoderm
hpf11:tailbud mesoderm
hpf12:tailbud mesoderm
hpf14:myotome
hpf18:myotome
hpf24:myotome
hpf36:myotome
Muscle
p_rht = 0.145
hpf48:muscle
Pharyngeal arch
p_rht = 0.499
hpf8:mesoderm lateral plate
hpf9:mesoderm lateral plate
hpf10:mesoderm lateral plate (tbx1+)
hpf11:mesoderm lateral plate (tbx1+)
hpf12:mesoderm lateral plate (tbx1+)
hpf14:pharyngeal arch
hpf18:pharyngeal arch
hpf24:pharyngeal arch
hpf36:pharyngeal arch
hpf48:pharyngeal arch

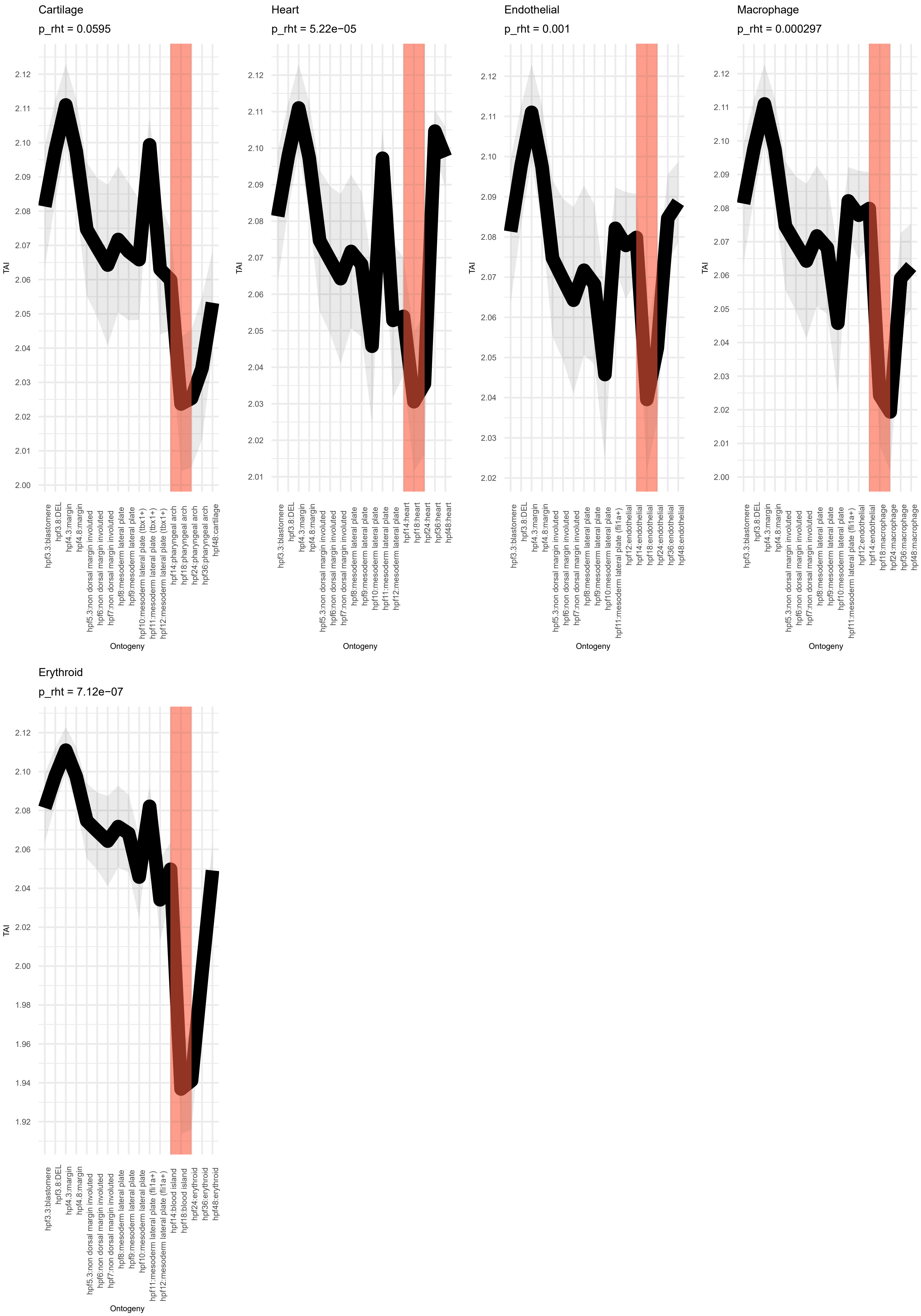
Cartilage
p_rht = 0.0595
Heart
p_rht = 5.22e−05
Endothelial
p_rht = 0.001
Macrophage
p_rht = 0.000297
Erythroid
p_rht = 7.12e−07
TAI
Ontogeny
hpf3.3:blastomere
hpf3.8:DEL
hpf4.3:margin
hpf4.8:margin
hpf5.3:non dorsal margin involuted
hpf6:non dorsal margin involuted
hpf7:non dorsal margin involuted
hpf8:mesoderm lateral plate
hpf9:mesoderm lateral plate
hpf10:mesoderm lateral plate (tbx1+)
hpf11:mesoderm lateral plate (tbx1+)
hpf12:mesoderm lateral plate (tbx1+)
hpf14:pharyngeal arch
hpf18:pharyngeal arch
hpf24:pharyngeal arch
hpf36:pharyngeal arch
hpf48:cartilage
hpf10:mesoderm lateral plate
hpf11:mesoderm lateral plate
hpf12:mesoderm lateral plate
hpf14:heart
hpf18:heart
hpf24:heart
hpf36:heart
hpf48:heart
hpf11:mesoderm lateral plate (fli1a+)
hpf12:endothelial
hpf14:endothelial
hpf18:endothelial
hpf24:endothelial
hpf36:endothelial
hpf48:endothelial
hpf10:mesoderm lateral plate (fli1a+)
hpf14:endothelial
hpf18:macrophage
hpf24:macrophage
hpf36:macrophage
hpf48:macrophage
hpf12:mesoderm lateral plate (fli1a+)
hpf14:blood island
hpf18:blood island
hpf24:erythroid
hpf36:erythroid
hpf48:erythroid

Supp. Fig. 10. Reductive hourglass tests for the TAI profile of individual zebrafish cellular trajectories using a denoised dataset. Significance was assessed using 10,000 permutations, with a p-value ≤ 0.05 considered significant. The hourglass pattern was tested by defining early stages as hpf3.3-hpf12, mid as hpf14-hpf24, and late as hpf36-hpf24.

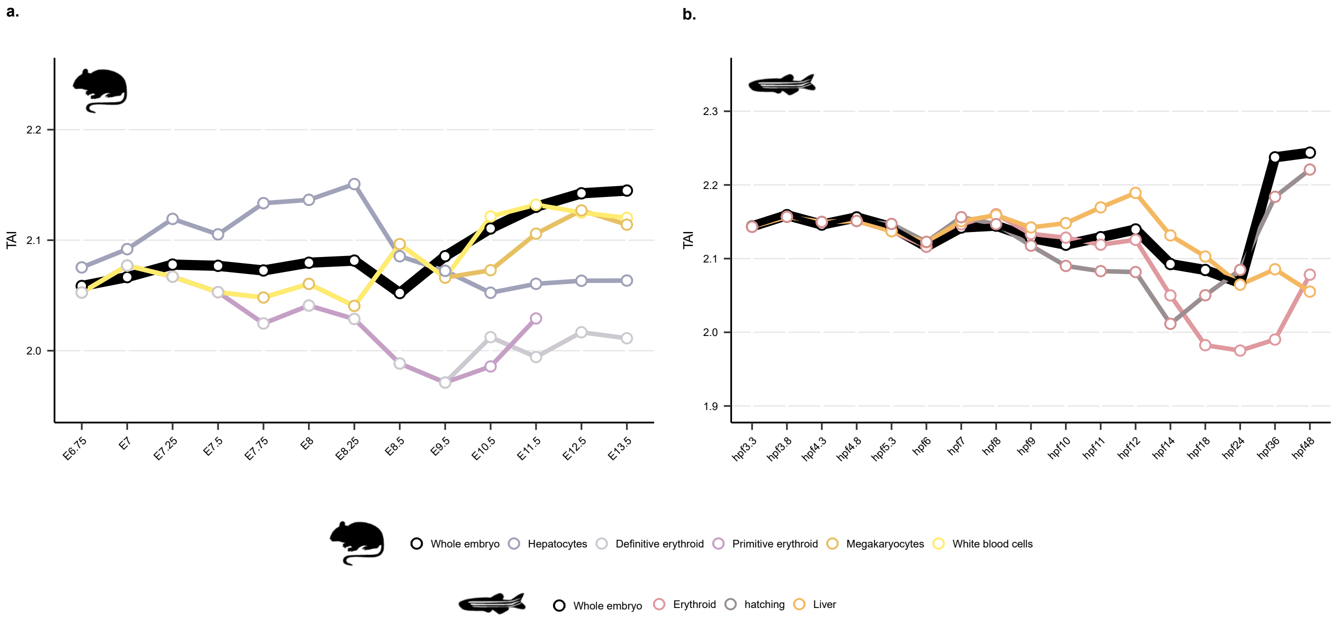

Supp. Fig. 11. TAI profiles for mouse (a) and zebrafish (b) cellular trajectories that depart from the hourglass signature. These lineages show significant deviations from the whole-embryo pattern (black line) and from the majority of cellular trajectories shown in Fig. 5c,d. Mouse and zebrafish silhouettes obtained from PhyloPic (https://www.phylopic.org); image credit Soledad Miranda-Rottmann and Ian Quigley, respectively (CC BY 3.0).

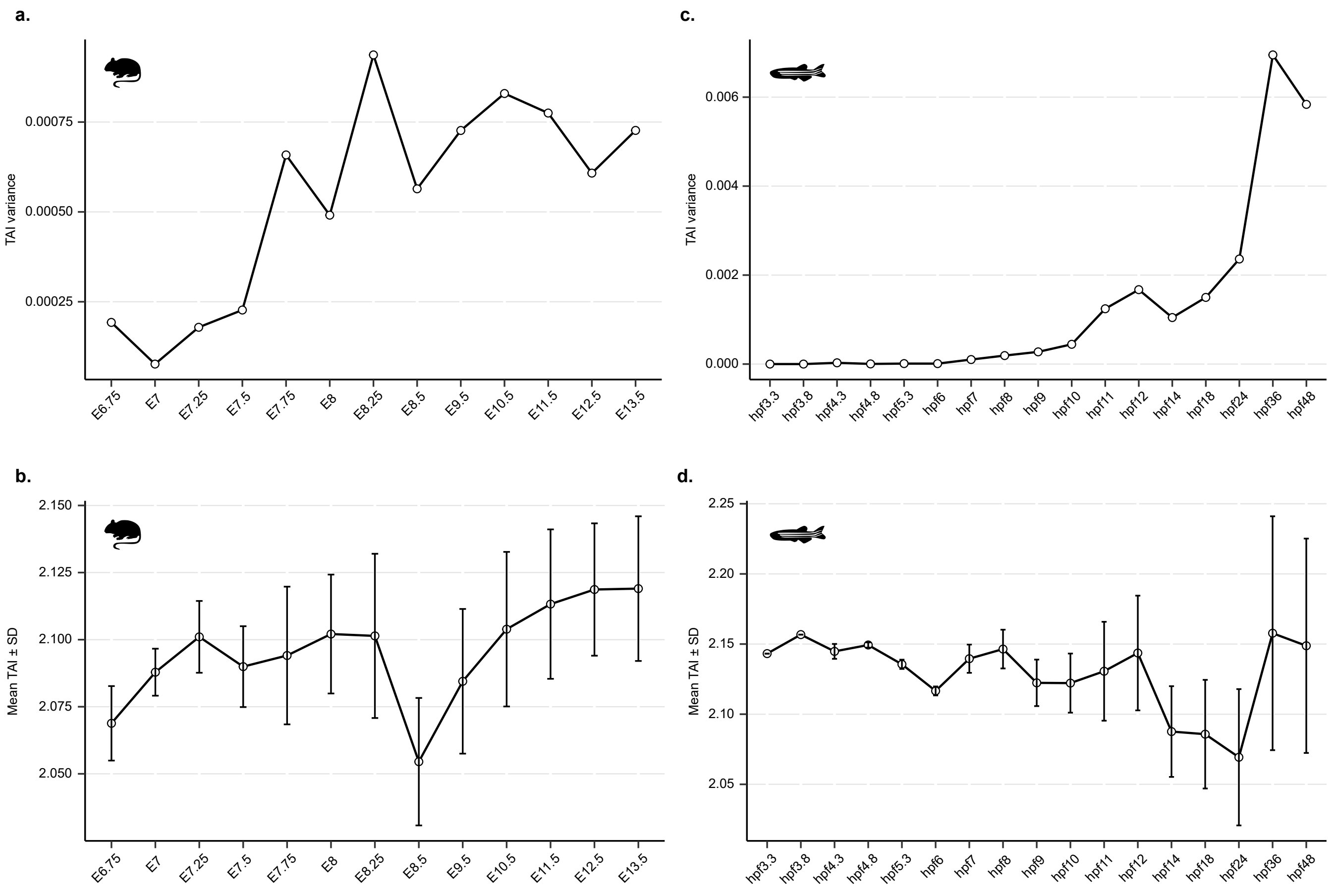

Supp. Fig. 12. Heterogeneity of transcriptome age across cell types during development. a, **b** Variance in TAI among unique cell types across developmental stages. **c**, **d** Mean± SD TAI of cell types across developmental stages. Note that heterogeneity increases as development proceeds. Mouse and zebrafish silhouettes obtained from PhyloPic (https://www.phylopic.org); image credit Soledad Miranda-Rottmann and Ian Quigley, respectively (CC BY 3.0).

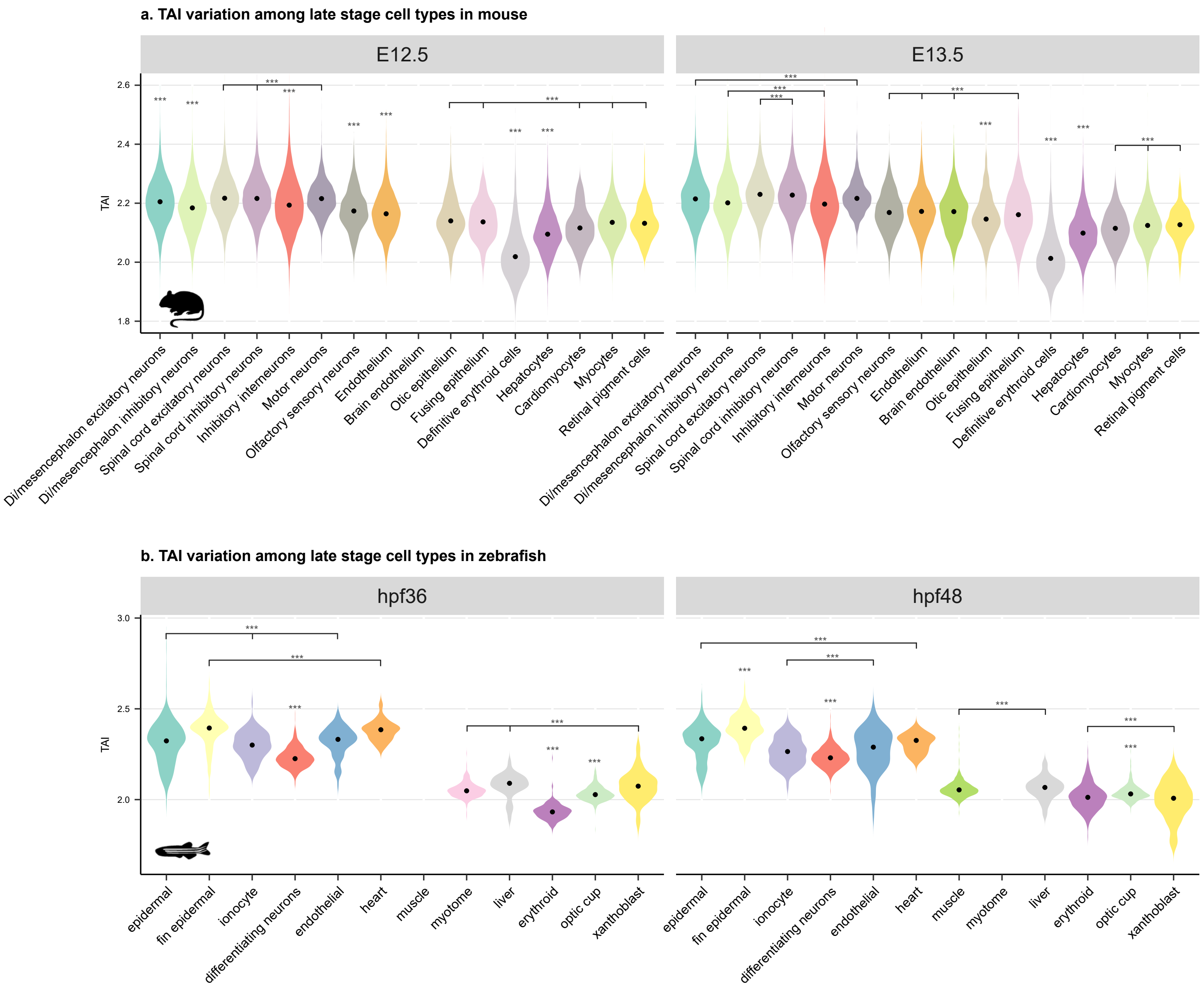

Supp. Fig. 13. Variation of transcriptome ages among late-stage cell types. a TAI values for cell types with the highest and lowest values at E12.5 and E13.5 in mouse. **b** TAI values for cell types with the highest and lowest values at hpf36 and hpf48 in zebrafish. Mouse and zebrafish silhouettes obtained from PhyloPic (https://www.phylopic.org); image credit Soledad Miranda-Rottmann and Ian Quigley, respectively (CC BY 3.0).

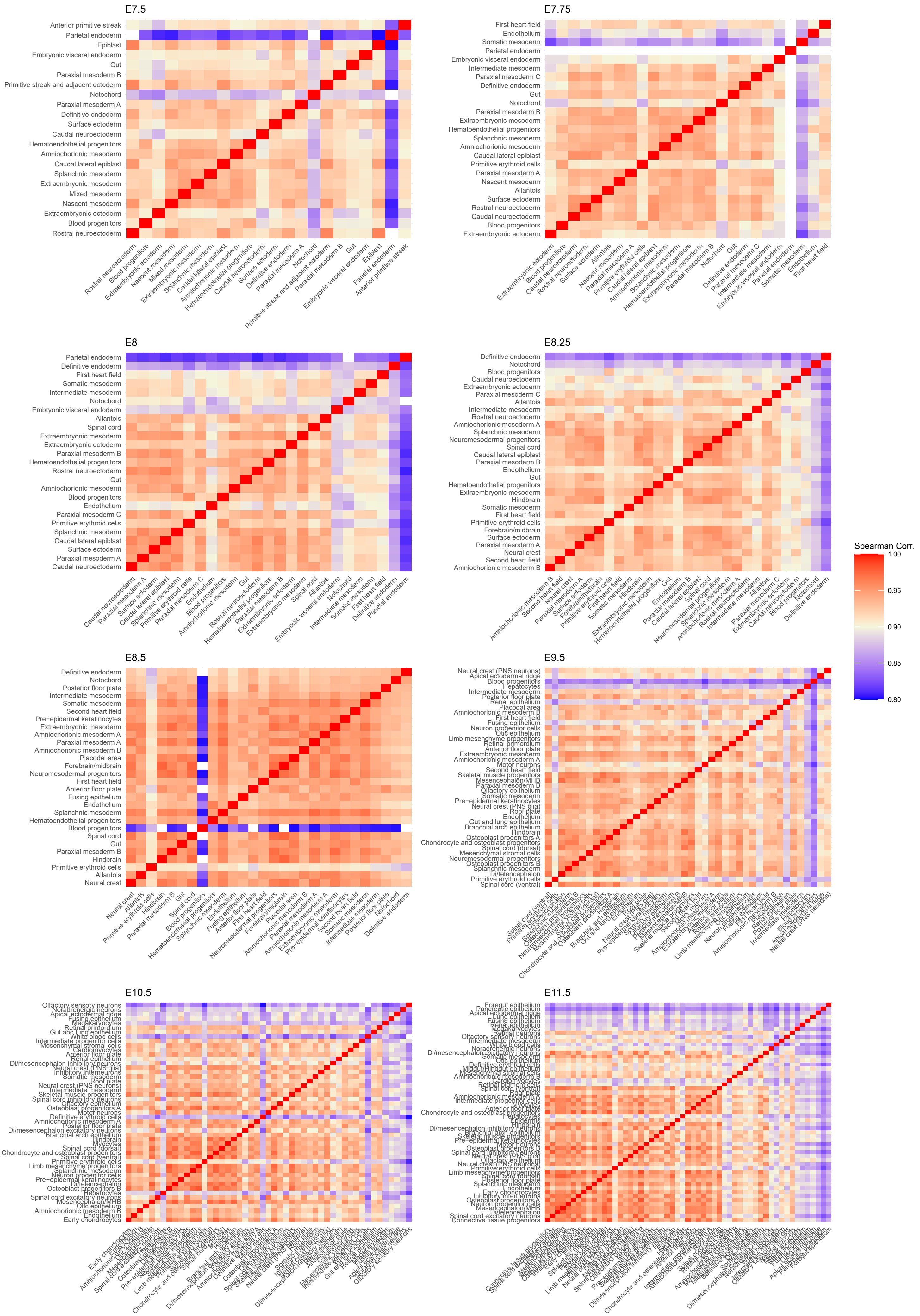
E7.5
Anterior primitive streak
Parietal endoderm
Epiblast
Embryonic visceral endoderm
Gut
Paraxial mesoderm B
Primitive streak and adjacent ectoderm
Notochord
Paraxial mesoderm A
Definitive endoderm
Surface ectoderm
Caudal neuroectoderm
Hematoendothelial progenitors
Amniochorionic mesoderm
Caudal lateral epiblast
Splanchnic mesoderm
Extraembryonic mesoderm
Mixed mesoderm
Nascent mesoderm
Extraembryonic ectoderm
Blood progenitors
Rostral neuroectoderm
E7.75
First heart field
Endothelium
Somatic mesoderm
Parietal endoderm
Embryonic visceral endoderm
Intermediate mesoderm
Paraxial mesoderm C
Definitive endoderm
Gut
Notochord
Paraxial mesoderm B
Extraembryonic mesoderm
Hematoendothelial progenitors
Splanchnic mesoderm
Amniochorionic mesoderm
Caudal lateral epiblast
Primitive erythroid cells
Paraxial mesoderm A
Nascent mesoderm
Allantois
Surface ectoderm
Rostral neuroectoderm
Caudal neuroectoderm
Blood progenitors
Extraembryonic ectoderm
E8
Parietal endoderm
Definitive endoderm
First heart field
Somatic mesoderm
Intermediate mesoderm
Notochord
Embryonic visceral endoderm
Allantois
Spinal cord
Extraembryonic mesoderm
Extraembryonic ectoderm
Paraxial mesoderm B
Hematoendothelial progenitors
Rostral neuroectoderm
Gut
Amniochorionic mesoderm
Blood progenitors
Endothelium
Paraxial mesoderm C
Primitive erythroid cells
Splanchnic mesoderm
Caudal lateral epiblast
Surface ectoderm
Paraxial mesoderm A
Caudal neuroectoderm
E8.25
Definitive endoderm
Notochord
Blood progenitors
Caudal neuroectoderm
Extraembryonic ectoderm
Paraxial mesoderm C
Allantois
Intermediate mesoderm
Rostral neuroectoderm
Amniochorionic mesoderm A
Splanchnic mesoderm
Neuromesodermal progenitors
Spinal cord
Caudal lateral epiblast
Paraxial mesoderm B
Endothelium
Gut
Hematoendothelial progenitors
Extraembryonic mesoderm
Hindbrain
Somatic mesoderm
First heart field
Primitive erythroid cells
Forebrain/midbrain
Surface ectoderm
Paraxial mesoderm A
Neural crest
Second heart field
Amniochorionic mesoderm B
Spearman Corr.
1.00
0.95
0.90
0.85
0.80
E8.5
Definitive endoderm
Notochord
Posterior floor plate
Intermediate mesoderm
Somatic mesoderm
Second heart field
Pre−epidermal keratinocytes
Extraembryonic mesoderm
Amniochorionic mesoderm A
Paraxial mesoderm A
Amniochorionic mesoderm B
Placodal area
Forebrain/midbrain
Neuromesodermal progenitors
First heart field
Anterior floor plate
Fusing epithelium
Endothelium
Splanchnic mesoderm
Hematoendothelial progenitors
Blood progenitors
Spinal cord
Gut
Paraxial mesoderm B
Hindbrain
Primitive erythroid cells
Allantois
Neural crest
E9.5
E10.5
E11.5

Supp. Fig. 14. Heatmap of pairwise transcriptome similarity across cell states within each mouse stage. Transcriptome similarity was quantified using Spearman's correlation coefficient (ρ).

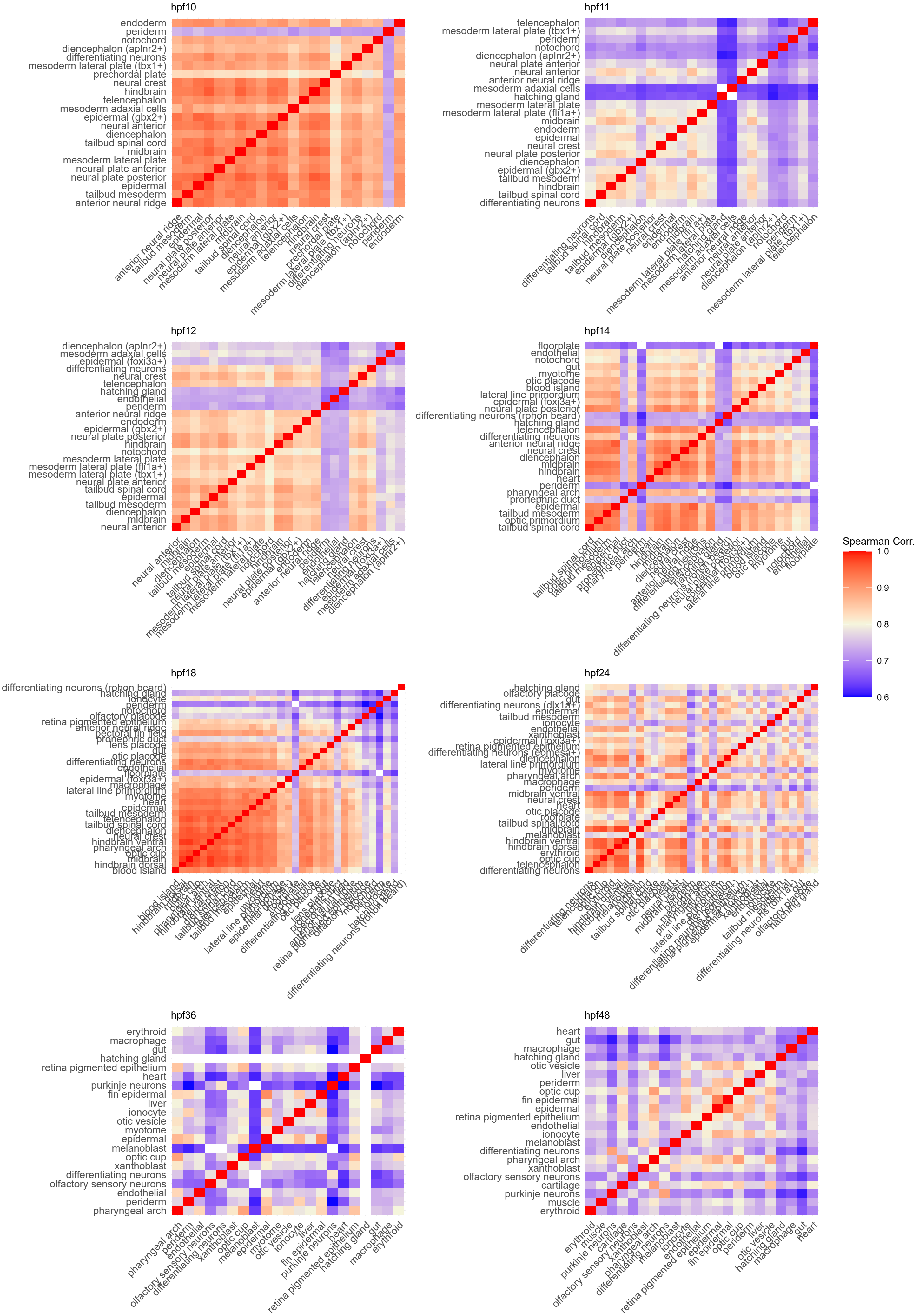

hpf10
endoderm
periderm
notochord
diencephalon (aplnr2+)
differentiating neurons
mesoderm lateral plate (tbx1+)
prechordal plate
neural crest
hindbrain
telencephalon
mesoderm adaxial cells
epidermal (gbx2+)
neural anterior
diencephalon
tailbud spinal cord
midbrain
mesoderm lateral plate
neural plate anterior
neural plate posterior
epidermal
tailbud mesoderm
anterior neural ridge
hpf11
telencephalon
mesoderm lateral plate (tbx1+)
periderm
notochord
diencephalon (aplnr2+)
neural plate anterior
neural anterior
anterior neural ridge
mesoderm adaxial cells
hatching gland
mesoderm lateral plate
mesoderm lateral plate (fli1a+)
midbrain
endoderm
epidermal
neural crest
neural plate posterior
diencephalon
epidermal (gbx2+)
tailbud mesoderm
hindbrain
tailbud spinal cord
differentiating neurons
hpf12
diencephalon (aplnr2+)
mesoderm adaxial cells
epidermal (foxi3a+)
differentiating neurons
neural crest
telencephalon
hatching gland
endothelial
periderm
anterior neural ridge
endoderm
epidermal (gbx2+)
neural plate posterior
hindbrain
notochord
mesoderm lateral plate
mesoderm lateral plate (fli1a+)
mesoderm lateral plate (tbx1+)
neural plate anterior
tailbud spinal cord
epidermal
tailbud mesoderm
diencephalon
midbrain
neural anterior
hpf14
floorplate
endothelial
notochord
gut
myotome
otic placode
blood island
lateral line primordium
epidermal (foxi3a+)
neural plate posterior
differentiating neurons (rohon beard)
hatching gland
telencephalon
differentiating neurons
anterior neural ridge
neural crest
diencephalon
midbrain
hindbrain
heart
periderm
pharyngeal arch
pronephric duct
epidermal
tailbud mesoderm
optic primordium
tailbud spinal cord
Spearman Corr.
1.0
0.9
0.8
0.7
0.6
hpf18
differentiating neurons (rohon beard)
hatching gland
ionocyte
periderm
notochord
olfactory placode
retina pigmented epithelium
anterior neural ridge
pectoral fin field
pronephric duct
lens placode
gut
otic placode
differentiating neurons
endothelial
floorplate
epidermal (foxi3a+)
macrophage
lateral line primordium
myotome
heart
epidermal
tailbud mesoderm
telencephalon
tailbud spinal cord
diencephalon
neural crest
hindbrain ventral
pharyngeal arch
optic cup
midbrain
hindbrain dorsal
blood island
hpf24
hatching gland
olfactory placode
gut
differentiating neurons (dlx1a+)
epidermal
tailbud mesoderm
ionocyte
endothelial
xanthoblast
epidermal (foxi3a+)
retina pigmented epithelium
differentiating neurons (eomesa+)
diencephalon
lateral line primordium
myotome
pharyngeal arch
macrophage
periderm
midbrain ventral
neural crest
heart
otic placode
floorplate
tailbud spinal cord
midbrain
melanoblast
hindbrain ventral
hindbrain dorsal
erythroid
optic cup
telencephalon
differentiating neurons
hpf36
erythroid
macrophage
gut
hatching gland
retina pigmented epithelium
heart
purkinje neurons
fin epidermal
liver
ionocyte
otic vesicle
myotome
epidermal
melanoblast
optic cup
xanthoblast
differentiating neurons
olfactory sensory neurons
endothelial
periderm
pharyngeal arch
hpf48
heart
gut
macrophage
hatching gland
otic vesicle
liver
periderm
optic cup
fin epidermal
epidermal
retina pigmented epithelium
endothelial
ionocyte
melanoblast
differentiating neurons
pharyngeal arch
xanthoblast
olfactory sensory neurons
cartilage
purkinje neurons
muscle
erythroid

Supp. Fig. 15. Heatmap of pairwise transcriptome similarity across cell states within each zebrafish stage. Transcriptome similarity was quantified using Spearman's correlation coefficient (ρ).

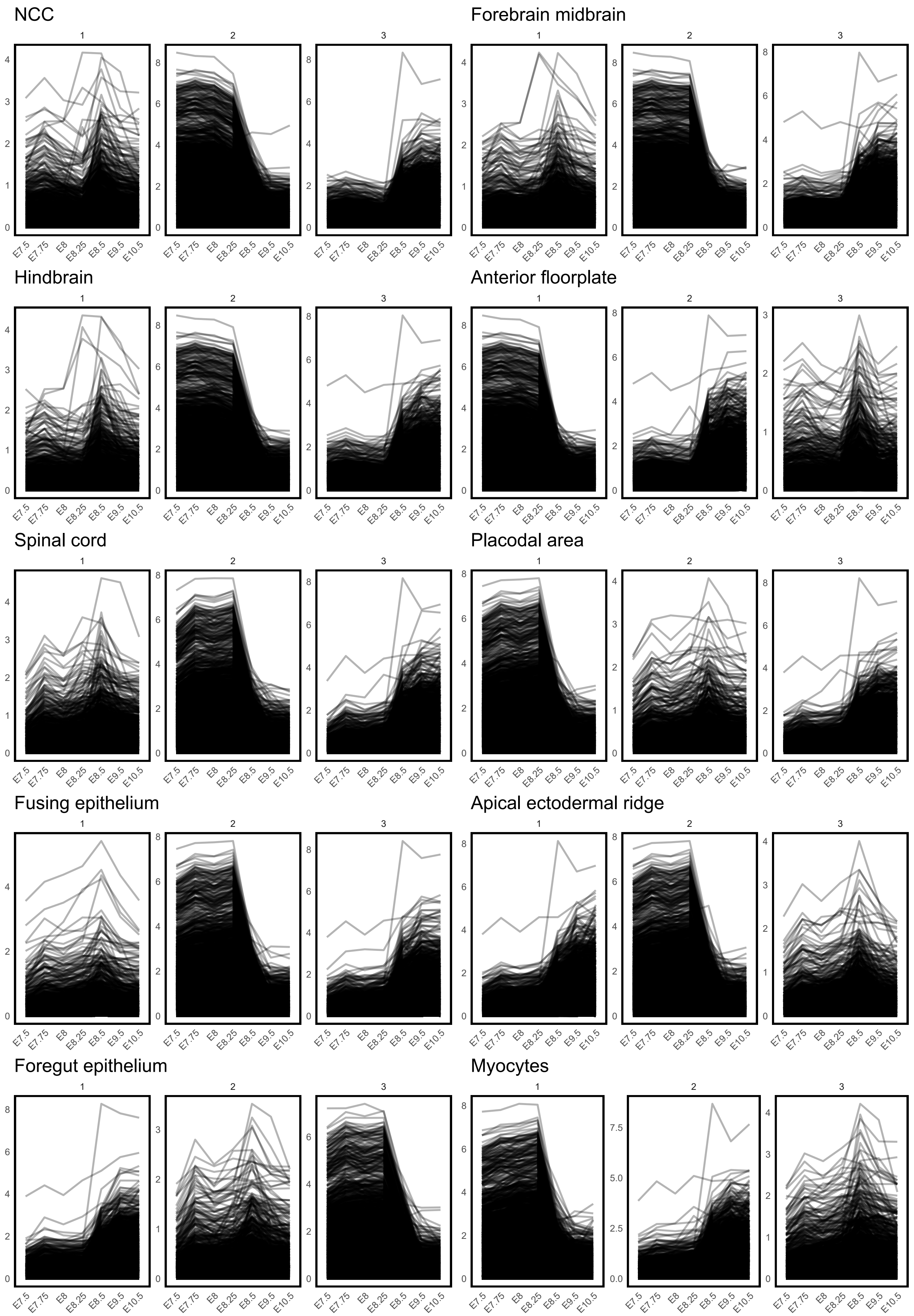
NCC
Forebrain midbrain
Hindbrain
Anterior floorplate
Spinal cord
Placodal area
Fusing epithelium
Apical ectodermal ridge
Foregut epithelium
Myocytes
1
2
3
E7.5
E7.75
E8
E8.25
E8.5
E9.5
E10.5

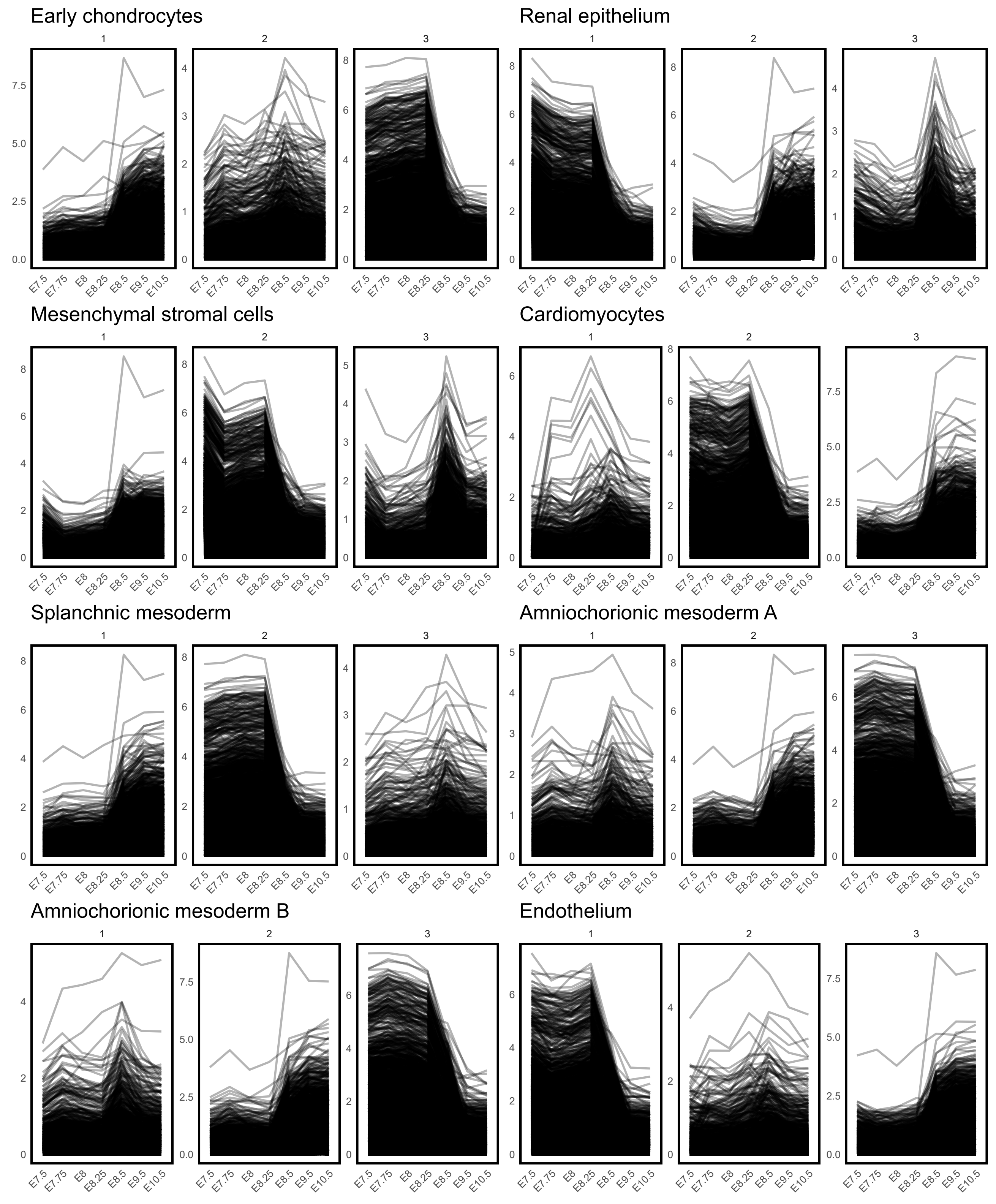

Supp. Fig. 16. Clustering of genes based on expression patterns along selected mouse cellular trajectories using k-means clustering.

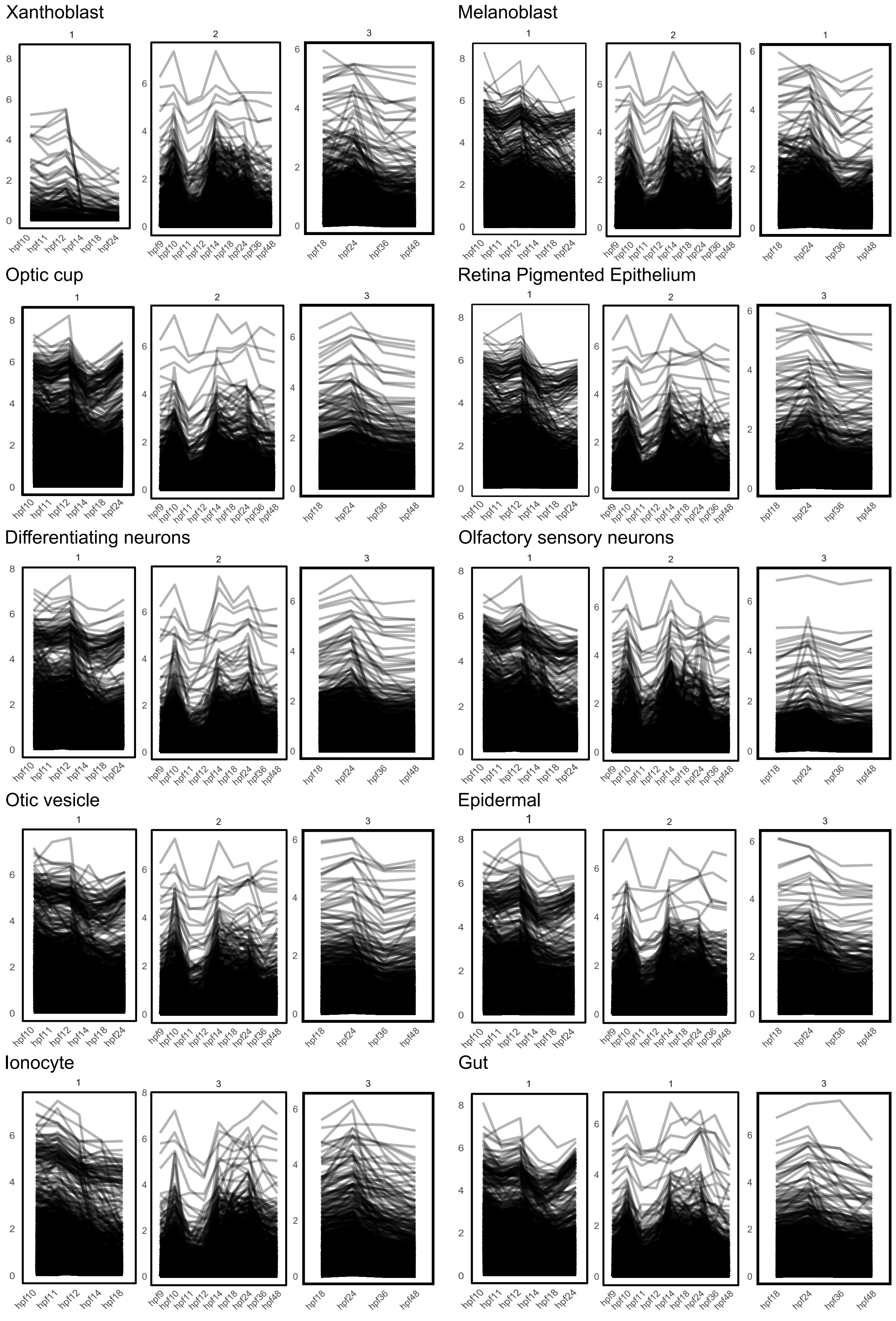
Xanthoblast
Melanoblast
Optic cup
Retina Pigmented Epithelium
Differentiating neurons
Olfactory sensory neurons
Otic vesicle
Epidermal
Ionocyte
Gut

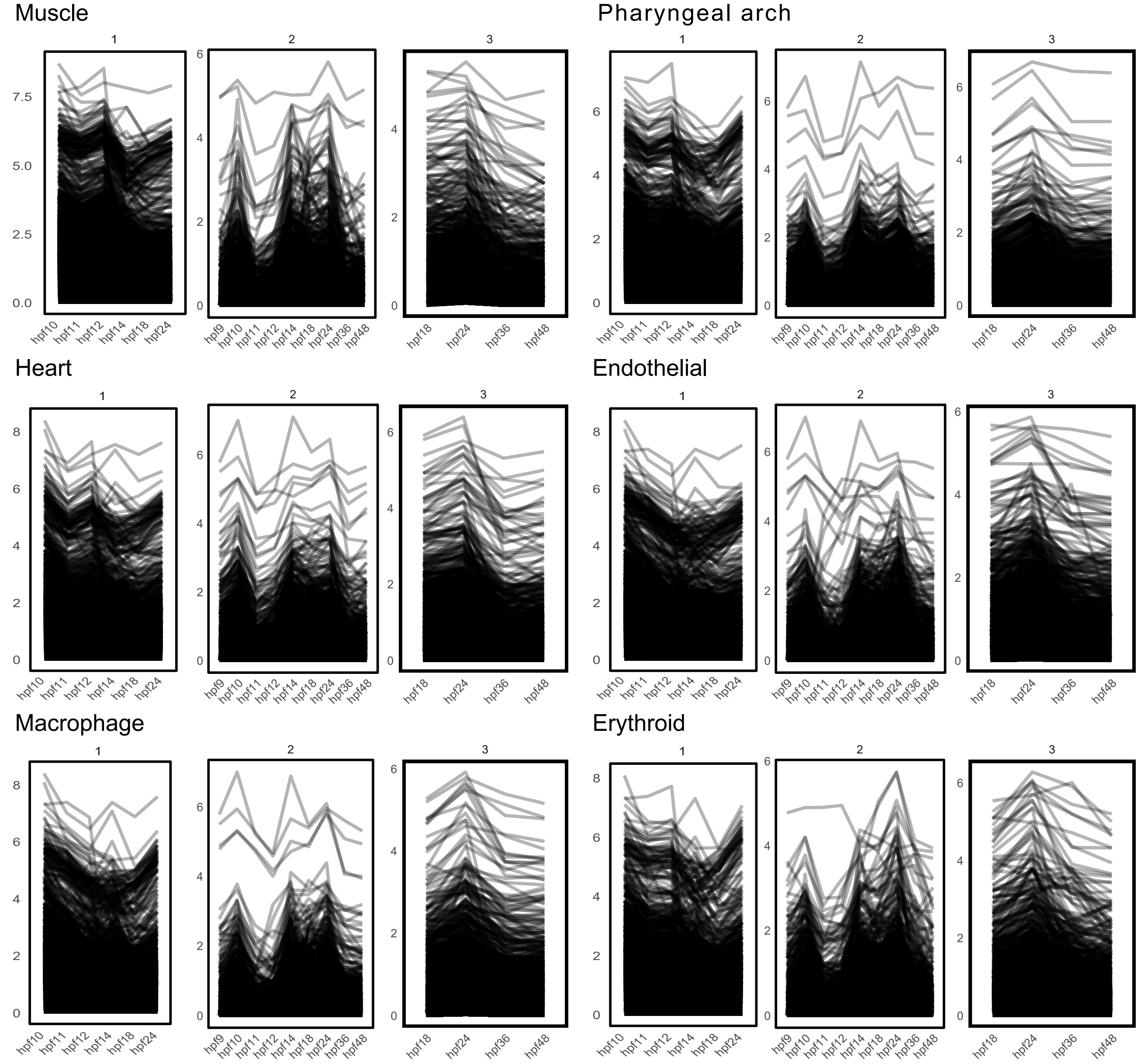

Supp. Fig. 17. Clustering of genes based on expression patterns along selected zebrafish cellular trajectories using k-means clustering.
