# Supplementary material for: A cellular basis for the hourglass pattern in vertebrate embryogenesis
Source: Nat Commun. 2026 Mar 10;17:2404. doi: 10.1038/s41467-026-69828-9 (PMC12982620; doi:10.1038/s41467-026-69828-9)
Supplement: Supplementary file 2 — Description of Additional Supplementary Files [file 41467_2026_69828_MOESM2_ESM.pdf]

File Name: Supplementary Data 1

Description: Gene age assignments

Phylostrata assignments of mouse and zebrafish protein-coding genes used to calculate the Transcriptome Age Index (Supp. Note 6).
